# Clinical course and outcomes of corneal blood staining in open-globe injury: Eye Injury Vitrectomy Study

**DOI:** 10.1371/journal.pone.0354967

**Published:** 2026-08-04

**Authors:** Bingjie Wang, Kang Feng, Yao Lu, Huijin Chen, Liang Han, Aihua Ding, Haikun Wang, Shaofeng Gu, Zhizhong Ma

**Affiliations:** 1 Department of Ophthalmology, Peking University Third Hospital, Beijing, China; 2 Eye Center, Beijing Tsinghua Changgung Hospital, School of Clinical Medicine, Tsinghua University, Beijing, China; Federal Medical Centre, Asaba, NIGERIA

## Abstract

**Purpose:**

To describe the clinical course and outcomes of corneal blood staining in open-globe injuries (OGIs) after vitrectomy without routine immediate allograft penetrating keratoplasty (PKP).

**Methods:**

This study included 42 eyes of 42 patients with corneal blood staining secondary to OGIs. Clinical data were retrospectively collected from the Eye Injury Vitrectomy Study (EIVS). All eyes underwent anterior chamber washout at the start of pars plana vitrectomy (PPV). When visualization remained inadequate, temporary keratoprosthesis (TKP)-assisted PPV was performed, followed by replacement of the trephined autologous cornea (TKP + PPV + RTC). Postoperative outcomes, including corneal transparency, best-corrected visual acuity (BCVA), intraocular pressure (IOP), and need for subsequent PKP, were assessed.

**Results:**

Median age was 36.5 years; globe rupture accounted for 95.2%. Preoperative hypotony (IOP ≤ 10 mmHg) was present in 90.5%, with severe posterior segment injury in all eyes. Eight eyes achieved sufficient corneal clarity after washout alone; the remaining 34 required TKP + PPV + RTC. During follow-up, varying degrees of spontaneous clearance occurred in most eyes, forming a peripheral “window” that enabled fundus assessment. At the final follow-up, BCVA improved from median 4.00 logMAR preoperatively to 2.80 logMAR (p = 0.002), and median IOP rose from 5.00 to 10.00 mmHg (p = 0.002). Five eyes developed phthisis bulbi, four eyes achieved complete silicone oil removal, and only 1 eye underwent allograft PKP.

**Conclusions:**

The strategy of anterior chamber washout first, followed by TKP + PPV + RTC when necessary, provides timely posterior segment access while preserving the globe and conserving corneal tissue in severe OGIs complicated by corneal blood staining. These findings support selective, rather than routine, PKP during follow-up.

## Introduction

Open-globe injuries (OGIs) are ocular injuries associated with the worst visual outcomes and may result in monocular or bilateral blindness, particularly in eyes with globe rupture [1,2]. Adequate corneal transparency is essential for visualization of posterior segment pathology. In severe ocular trauma, however, corneal transparency may be compromised, limiting clinical assessment and impeding surgical repair.

Among trauma-related corneal opacities, corneal blood staining is one of the most challenging conditions. It is characterized by deposition of hemoglobin and its breakdown products within the corneal stroma [3], most commonly following prolonged hyphema and elevation of intraocular pressure (IOP) [4,5], but it has also been reported in eyes with low IOP [6,7]. Disruption of endothelial function, stromal impregnation by erythrocyte derivatives, and impaired metabolic clearance contribute to its development [8].

In eyes with corneal blood staining requiring pars plana vitrectomy (PPV), a temporary keratoprosthesis (TKP) is commonly used to facilitate posterior segment visualization [9]. This approach, however, necessitates donor tissue for subsequent allograft penetrating keratoplasty (PKP) and is associated with risks of graft rejection and infection related to long-term immunosuppression [10]. Furthermore, given the poor prognosis in eyes with extensive posterior segment damage, the indication for immediate PKP may be uncertain. Although endoscopy-assisted vitrectomy has been proposed as an alternative, its adoption is limited by low image resolution, restricted field of view, specialized equipment requirements, and a steep learning curve [11–13].

In this study, we evaluated a modified corneal conservative surgical strategy for OGIs complicated by corneal blood staining, and analyzed anatomical and functional outcomes, as well as the evolution of corneal blood staining.

## Methods

### Human ethics and consent to participate declarations

This study was approved by the Institutional Review Board of Peking University Third Hospital Medical Science Research Ethics Committee (Ethics No. IRB00006761-2012060) and was conducted in accordance with the Declaration of Helsinki. Clinical data were collected from the Eye Injury Vitrectomy Study (EIVS). The trial was registered with the Chinese Clinical Trial Registry (ChiCTR; Number: ChiCTR-OCH-08000199) on November 28, 2008. Written informed consent was obtained from all participants at the time of enrollment in the EIVS, including consent for the use of non-personally identifiable medical records for future research. For participants younger than 18 years of age, written informed consent was obtained from their parents or legal guardians. The present study represents a retrospective analysis of EIVS data from Peking University Third Hospital between June 2012 and June 2025. As defined by the EIVS database, the general inclusion criteria were patients who suffered from severe eye injury and were treated with vitreoretinal surgery, enucleation, or evisceration [14]. Throughout data collection and analysis, the investigators had access only to fully anonymized data and no personal identifiable information. Given the retrospective nature of this study and the use of anonymized data, the ethics committee waived the requirement for additional informed consent.

### Participants and clinical evaluation

Patients with OGIs complicated by corneal blood staining were included. Exclusion criteria were endophthalmitis, pre-existing ocular pathology, incomplete clinical or photographic records, follow-up < 12 months, or enucleation/evisceration within 12 months (S1 File).

Preoperative data collected included age, sex, ocular history, best-corrected visual acuity (BCVA), IOP, slit-lamp photographs, and anterior segment optical coherence tomography (AS-OCT). BCVA was measured using a standard vision chart and converted into logarithm of the minimum angle of resolution (logMAR). For statistical consistency, counting fingers, hand motion, light perception, and no light perception (NLP) were converted to 1.87, 2.30, 2.80, and 4.00 logMAR, respectively. IOP was measured using a rebound tonometer (iCare TA01i, Icare Finland Oy, Helsinki, Finland). AS-OCT (Wavelength nanometer, Tomey CASIA2, Nagoya, Japan) was performed by trained clinicians (AD, HW, and SG), with serial scans were obtained from same corneal regions whenever possible, using anatomical landmarks and previous scan locations as references.

All patients underwent anterior chamber washout at the start of the initial PPV. Sufficient clarity for PPV after washout was defined as intraoperative corneal clarity that allowed the surgeon to identify major posterior segment structures and proceed safely without TKP. When safe visualization could not be achieved, TKP-assisted PPV was performed, followed by replacement of the trephined autologous cornea (TKP + PPV + RTC). The surgical procedure for TKP + PPV + RTC was described previously [15]. Follow-up duration varied among patients. BCVA, IOP, and slit-lamp photographs were recorded at each visit. Additional treatments were provided as clinically indicated.

Postoperative corneal outcomes were categorized as complete clearing, partial clearing, or persistent opacity. Complete clearing was defined as disappearance of opacity attributable to corneal blood staining during follow-up, although other corneal changes such as scarring or band keratopathy could still be present. Partial clearing was defined as a recognizable decrease in corneal blood staining compared with baseline, with formation of a clear corneal area sufficient for clinical posterior segment assessment. Persistent opacity was defined as residual opacity attributable to corneal blood staining with little or no regression compared with baseline, continuing to limit corneal transparency or fundus visualization. Serial slit-lamp photographs, fundus photographs when available, and clinical records were independently reviewed by two ophthalmologists (KF and LH) to classify the corneal outcomes. Disagreements were resolved by a senior ophthalmologist (ZM).

### Statistical analyses

Data were analyzed using SPSS software (version 26.0, IBM Corp., Armonk, NY, USA) and GraphPad Prism 8 (GraphPad Software, San Diego, CA, USA). Descriptive statistics were reported as median (interquartile range, IQR) for non-normally distributed variables or mean ± standard deviation for normally distributed variables. Preoperative and postoperative parameters were compared using the Wilcoxon matched-pairs signed rank test. A p-value of < 0.05 was considered statistically significant.

## Result

A total of 3,152 eyes in the EIVS database were screened, including 1,920 eyes with open-globe injuries. Among them, 157 eyes had documented corneal blood staining. After excluding 115 eyes because of endophthalmitis, pre-existing ocular diseases, incomplete clinical or photographic records, follow-up shorter than 12 months, or enucleation/evisceration within 12 months; 42 eyes (42 patients, 37 patients [88.1%] were male; 16 right eyes) were included in the final analysis (S1 File). The median age was 36.5 years (range, 3–61 years). The mechanism of injury was globe rupture in 40 eyes (95.2%), penetrating injury in 1 eye (2.4%), and intraocular foreign body (IOFB) in 1 eye (2.4%). The median interval from trauma to surgery was 21.5 days (IQR, 15.0–28.5 days).

Preoperative ocular hypotony (intraocular pressure [IOP] ≤10 mmHg) was present in 38 eyes (90.5%). Severe posterior segment injury was common: choroidal detachment occurred in 40 eyes, retinal detachment in 37 eyes, and no identifiable retinal tissue was noted in 5 eyes. All eyes received intraocular tamponade with silicone oil (n = 40) or long-acting gas (n = 2). The median follow-up duration was 36 months (IQR, 23–42 months; range, 12–60 months). During follow-up, most eyes required additional silicone oil procedures (e.g., oil refill, partial removal, or removal with re-tamponade), and complete silicone oil removal was achieved in only 4 eyes by the final visit.

**Fig 1:**
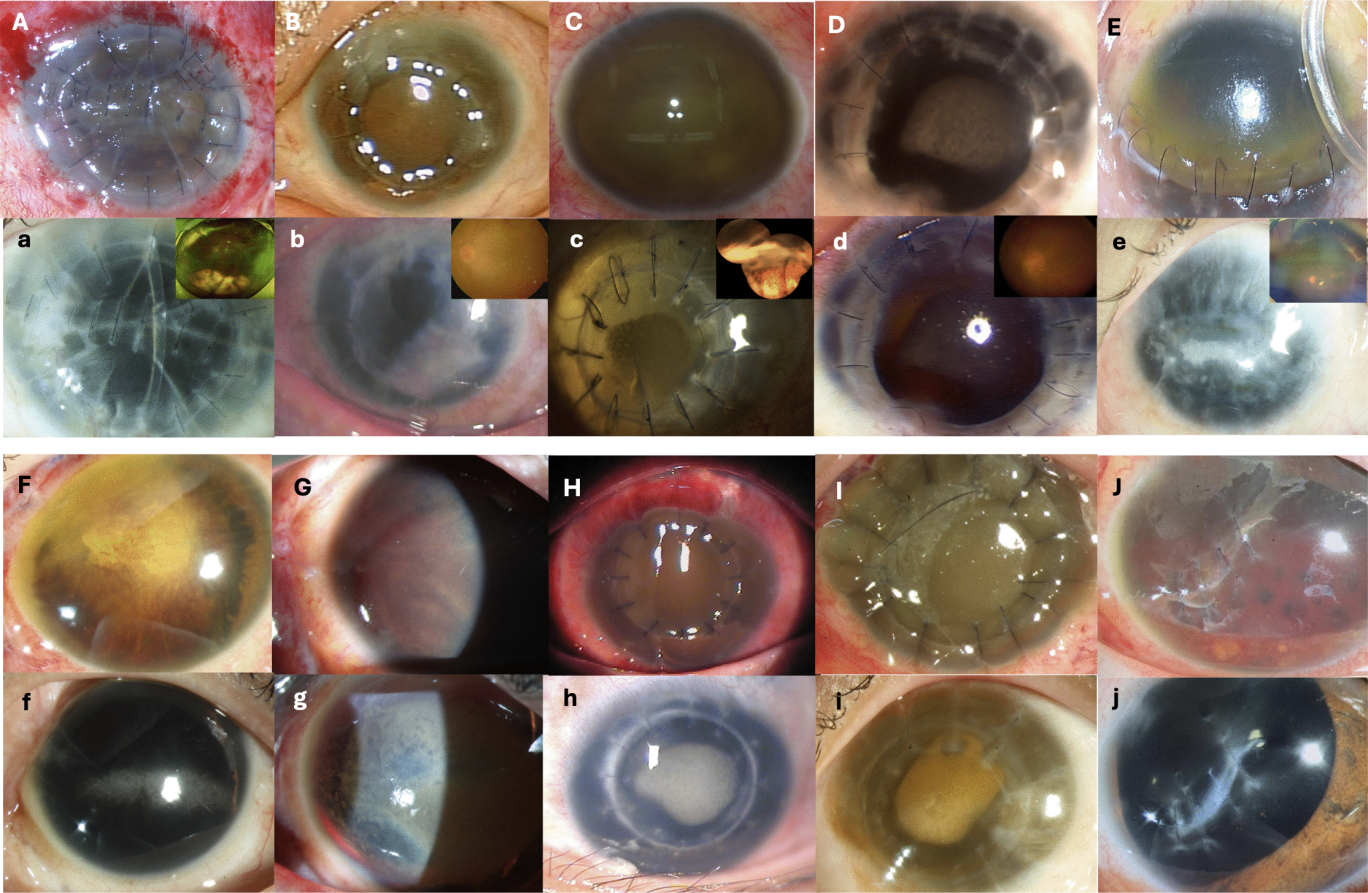
Anterior segment photographs of corneal blood staining before and after clearing, with corresponding fundus views. This series focuses on corneal changes and the possibility for fundus assessment; concomitant intraocular comorbidities were not considered. Each case is presented as paired panels, with the corresponding fundus image displayed in the upper right corner (a–e). (A, a) Immediate postoperative and postoperative month 6 (history of radial keratotomy). Vascularization at periphery cornea can be seen. (B, b) Postoperative months 2 and 17. (C, c) Intraoperative and postoperative month 12. (D, d) Postoperative months 6 and 24. (E, e) Intraoperative and postoperative month 48. (F, f) Preoperative and postoperative month 7, showing band keratopathy. (G, g) Preoperative and postoperative day 1. (H, h) Immediate postoperative and postoperative month 24, showing central corneal opacity. (I, i) Postoperative day 1 and postoperative month 15. (J, j) Preoperative and postoperative month 4.

**Fig 2:**
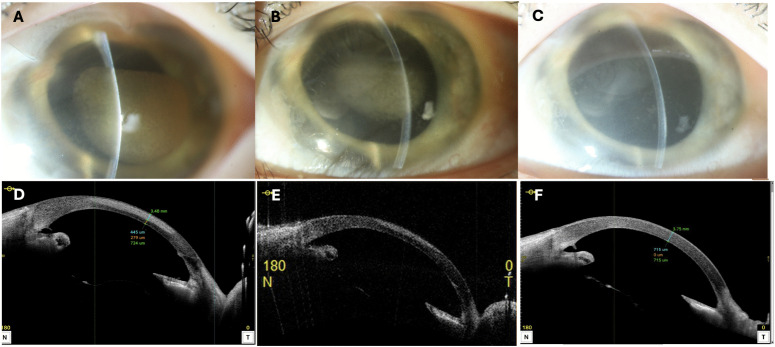
Slit-lamp photographs and AS-OCT demonstrating complete resolution of corneal blood staining. (A, D) Postoperative month 4; (B, E) postoperative month 10; (C, F) postoperative month 16.

After anterior chamber washout, adequate corneal clarity to proceed with PPV was achieved in 8 eyes, whereas the remaining 34 eyes required TKP + PPV + RTC. During follow-up, clearing of cornea blood staining was observed 35/42 eyes. In the anterior chamber washout only group, complete clearing occurred in 4 eyes and partial clearing in 4 eyes. In the TKP + PPV + RTC group, complete clearing occurred in 9 eyes, partial clearing in 18 eyes, and persistent opacity in 7 eyes. The first documented timing of corneal clearing is summarized in S2 File. Complete or partial clearing formed a peripheral “window” that permitted fundus assessment (Fig 1a–1e). At the final follow-up, persistent central opacity or scarring was observed in 17 of 42 eyes, including band keratopathy (9/42), central corneal scarring, and residual blood staining (Fig 1). Fig 2 presents serial AS-OCT images of a representative case from the complete clearing group managed with anterior chamber washout only, showing gradual clearing of corneal blood staining in this eye.

Preoperatively, 28 eyes (66.7%) had NLP, which decreased to 12 eyes (28.6%) after surgery. At the final follow-up, phthisis bulbi developed in 5 eyes. Baseline characteristics and perioperative outcomes are summarized in Table 1. Only one eye underwent delayed allograft PKP. This was a gas-tamponaded eye in a 46-year-old man with traumatic aniridia, aphakia after prior lens extraction in local hospital, vitreous hemorrhage, and post-traumatic secondary glaucoma. Although preoperative B-scan suggested choroidal detachment, this finding were not confirmed intraoperatively, and laser photocoagulation was applied around the retinal break after retinal attachment. The RTC gradually clearing, and BCVA reached 0.3 logMAR at 14 months after the initial surgery, with well-controlled IOP, indicating favorable visual potential and a relatively stable posterior segment. Allograft PKP combined with scleral-fixated IOL implantation was therefore performed for optical rehabilitation and aphakia correction. Early post-PKP follow-up was available, but the patient was subsequently lost to follow-up. When he returned 3 years after PKP, the eye had progressed to NLP due to glaucoma, with epithelial edema but relatively preserved stromal clarity in corneal graft.

**Table 1:** Patient demographics, preoperative/postoperative ocular findings.

	Patients Enrolled (n = 42)
Age, median (IQR) (years)	36.5 (25.8–49.3)
Right eye, n (%)	16 (38.1)
Male, n (%)	37 (88.1)
Surgery time interval, median (IQR) (days)	21.5 (15.0–28.5)
Open globe injury, n (%)	42 (100)
Rupture	40 (95.2)
Penetrating	1 (2.4)
Intraocular foreign bodies (IOFB)	1 (2.4)
Hypotony, n (%)	38 (90.5)
Retinal and choroidal condition, n (%)	
Choroidal detachment	40 (95.2)
Retinal detachment	37 (88.1)
No retina detected	5 (11.9)
Intraocular tamponade, n (%)	42 (100)
Silicone oil	40 (95.2)
Long-acting gas	2 (4.8)
Balanced salt solution	0 (0)
Cornea management, n (%)	
TKP + PPV + RTC	34 (81.0)
After anterior chamber washout, corneal transparency was sufficient for PPV	8 (19.0)
Follow-up duration, median (IQR) (months)	36 (23–42)
NLP preoperatively, n (%)	28 (66.7)
NLP postoperatively, n (%)	12 (28.6)
Phthisis bulbi at the final follow-up, n (%)	5 (11.9)
SOR at the final follow-up, n (%)	4/40 (10)
Received PKP at the final follow-up, n (%)	1 (2.4), tamponade with gas
Cornea outcome, n (%)	
TKP + PPV + RTC	
Complete clearing	9/34 (26.5)
Partial clearing	18/34 (52.9)
Persistent opacity	7/34 (20.6)
Anterior chamber washout only	
Complete clearing	4/8 (50)
Partial clearing	4/8 (50)
Persistent opacity	0/8 (0)

**Fig 3:**
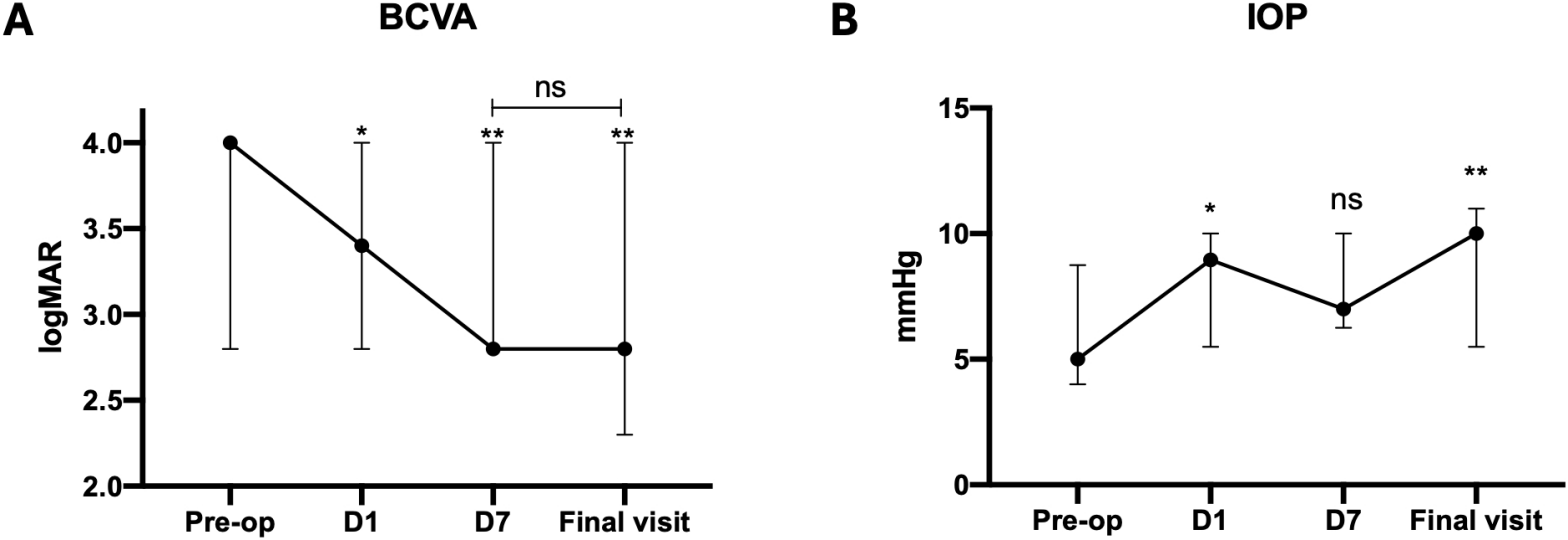
Changes in BCVA and IOP preoperative/postoperative. (A) BCVA demonstrated significant improvement at D1, D7 and the final visit comparing with preoperative. No significant improvement was observed at final visit when compared with D7 (n = 42). (B) IOP significantly increased at D1, and dropped at D7 to preoperative level. At final visit, IOP showed significant increase compared with pre-op, this process includes multiple silicone oil refilling in some cases. Median with IQR, Wilcoxon matched-pairs signed-rank test; *p < 0.05, **p < 0.01. Abbreviations: Pre-op, preoperative; D1, postoperative day 1; D7, postoperative day 7.

Median BCVA improved from 4.00 logMAR preoperatively (IQR, 2.80–4.00) to 3.40 logMAR on day 1 (IQR, 2.80–4.00; p = 0.016) and 2.80 logMAR on day 7 (IQR, 2.80–4.00; p = 0.002), as well as at the final visit (median, 2.80 logMAR; IQR, 2.30–4.00; p = 0.002). BCVA showed no significant difference between day 7 and the final visit (p = 0.344) (Fig 3A).

Median IOP increased from 5.00 mmHg preoperatively (IQR, 4.00–8.75) to 8.95 mmHg on day 1 (IQR, 5.50–10.00; p = 0.019), measured 7.00 mmHg on day 7 (IQR, 6.25–10.00, p = 0.401), and reached 10.00 mmHg at the final visit (IQR, 5.50–11.00, p = 0.002) (Fig 3B).

## Discussion

Management of corneal blood staining in the setting of complex ocular trauma remains challenging. In this series, we applied a modified approach (anterior chamber washout first, then TKP + PPV + RTC if needed) instead of performing immediate allogeneic PKP. A key observation was that corneal blood staining frequently showed spontaneous partial-to-complete resolution after surgery, often providing a peripheral “window” that permitted postoperative fundus assessment (Fig 1a–1e). For eyes with corneal opacity that limited direct visualization, including during the early postoperative period (e.g., at 1 week), posterior segment assessment was performed using B-scan ultrasonography, visual function examinations, and IOP measurement. The decision to perform subsequent PKP is strictly based on a comprehensive evaluation of both visual function and the anatomical viability of the injured eye. Only one eye ultimately underwent PKP. These findings support a conservative corneal strategy in eyes with limited visual potential and high graft-risk profiles. Conventionally, surgery has relied on immediate allogeneic PKP following TKP-assisted PPV. However, this strategy exposes the corneal graft to pathophysiologic stressors that are common in severely injured eyes, and may increase exposure to prolonged postoperative corticosteroid or immunosuppressive therapy as well as the risk of graft rejection in eyes with limited visual potential.

OGIs often leads to persistent hypotony and silicone-oil sustained [15,16]. In OGIs, hypotony can arise from direct ciliary body injury, cyclodialysis, ischemia and/or membrane formation of ciliary body, leading to reduced aqueous humor production and/or increased outflow through the suprachoroidal space [17–19]. Under these conditions, impaired aqueous circulation compromises corneal metabolic support, while sustained contact between silicone oil and the corneal endothelium accelerates endothelial dysfunction [20]. Together, these factors increase the risk of graft decompensation and failure after PKP [21].

Inflammation is another problem. Eyes with OGIs are commonly in an active inflammatory state at the time of PPV, which is a well-established independent risk factor for graft failure [22]. Inflammation induces upregulation of major histocompatibility complex (MHC) class I and class II antigen expression [23] and promotes vascular endothelial growth factor production, thereby facilitating graft rejection and corneal neovascularization [24]. Consistent with these mechanisms, Williams et al. showed that past ocular inflammation increased the risk of graft failure by 4.40-fold, while active inflammation increased the risk to 9.6-fold [25]. Accordingly, delaying PKP until inflammation has stabilized may reduce the risk of failure. The clinical importance of PKP timing has been supported by prior studies; for example, Roters et al. reported a significantly lower rate of graft failure when PKP was performed after 8 weeks of trauma [21]. Although delaying surgery may allow neovascularization to develop, as shown in Fig 1a, resulting in a vascularized graft bed. This trade-off may be acceptable, as the associated risk (odds ratio, 2.74 [25]) is substantially lower than that posed by active inflammation.

Importantly, the overall prognosis of OGIs is often poor [15]. All eyes in our series had severe vision-threatening pathology, including choroidal detachment, retinal detachment, or retinal tissue loss. Twenty-eight eyes presented with no light perception (NLP), and 12 remained NLP after surgery. During follow-up, 11.9% progressed to phthisis, indicating irreversible anatomical failure. In addition, silicone oil removal, which is critical for graft survival, was achieved in only 10% of our cases. Under these conditions, corneal transplantation may offer limited functional benefit in many eyes. Other global assessment of ocular viability—such as IOP trends, residual light perception, and B scan findings—may be more informative in guiding management decisions [26–28]. Consistent with this reality, only one eye underwent PKP in our study, suggesting that most patients were poor candidates for primary keratoplasty; for these individuals, TKP + PPV + RTC represents a more cost-effective and practical strategy. This selective approach is also vital when considering the critical global shortage of donor tissue, where only one cornea is available for every 70 patients in need [29].

The natural course of corneal blood staining further supports a conservative surgical strategy. Several case reports have described spontaneous resolution of corneal blood staining [30,31]. Clearance typically progresses from the peripheral cornea toward the center and from posterior to anterior layers [31], consistent with our observations. This pattern has been attributed to limbal circulation and shedding of iron-containing epithelial cells [8]. Conjunctival flap coverage may further accelerate this process [32], possibly by increasing local blood supply. In our series, most eyes showed varying degrees of spontaneous clearance during follow-up, which enabled posterior segment assessment through this window. Fig 2 illustrates one case with complete clearance, the AS-OCT result showed a progressive reduction and eventual disappearance of hyperreflective stromal opacity.

Although corneal blood staining may clear spontaneously, this process can take months to years [5,30–32]. In contrast, the timing of PPV after severe ocular trauma is critical, as delayed intervention may increase the risk of proliferative vitreoretinopathy and lead to irreversible anatomical or functional loss [2,33,34]. Therefore, timely posterior segment access while avoiding unnecessary high-risk allograft PKP in the acute traumatic setting is essential. Our strategy addresses this dilemma by performing anterior chamber washout first, followed by TKP + PPV + RTC when visualization remains inadequate. Compared with conventional TKP-assisted PPV followed by immediate allograft PKP, this approach avoids placing donor tissue into an acutely inflamed and often silicone-oil-filled eye. Compared with endoscopy-assisted vitrectomy, it preserves a conventional wide-field microscopic surgical view and does not require specialized endoscopic equipment or a separate technical learning. Thus, this approach may provide a practical alternative for managing severe OGIs with corneal blood staining, particularly in centers where endoscopic vitreoretinal surgery is not routinely available.

### Limitation

This study has several limitations. First, it was a retrospective analysis of a small cohort with highly heterogeneous severe ocular trauma, including variability in injury mechanism, posterior segment pathology, tamponade status, and follow-up duration. These factors, together with differences in the severity of corneal blood staining and corneal tissue damage, may have influenced the timing and extent of corneal clearing. Second, follow-up visits and AS-OCT imaging were not performed at standardized intervals. Therefore, the exact onset of corneal clearing could not be determined for every eye, and quantitative cohort-level AS-OCT analyses were not feasible. The analysis provided in the supplementary materials should be interpreted cautiously because of the limited sample size and substantial baseline imbalance. Finally, in this cohort of severe OGIs, final visual and anatomical outcomes were largely determined by posterior segment damage and overall ocular viability. Thus, the independent contribution of corneal blood staining to final BCVA, phthisis bulbi, or complete silicone oil removal could not be reliably isolated. Larger, prospective studies with standardized imaging, clinical grading, and follow-up protocols are needed to better define predictors of spontaneous clearance and to refine timing and selection of subsequent keratoplasty.

## Conclusion

This conservative corneal strategy provides timely posterior segment access in severe OGIs complicated by corneal blood staining while preserving the globe and conserving corneal tissue. By avoiding routine immediate allograft PKP in the acute traumatic setting, it allows subsequent keratoplasty decisions to be guided by ocular stability, corneal status, and visual potential. These findings support selective, rather than routine, allograft PKP in such severely injured eyes.

**Key messages**

**What is known**

- Corneal blood staining in open-globe injuries (OGIs) traditionally requires immediate allograft penetrating keratoplasty (PKP) to enable posterior segment access.
- Immediate PKP in the acute phase is high-risk due to intense inflammation, hypotony, and long-term silicone oil tamponade.

**What is new**

- This study highlights a globe-preserving and cornea-conserving strategy for severe OGIs complicated by corneal blood staining, using anterior chamber washout first, and TKP-assisted PPV with replacement of the trephined autologous cornea when necessary.
- By avoiding routine immediate allograft PKP in the acute traumatic setting, this approach preserves the opportunity for later keratoplasty to be selected after ocular stabilization, based on overall ocular status, corneal condition, and visual potential, while reducing graft-related risks.

## Supporting information

S1 File Flow diagram of the study population enrollment from the EIVS database. (PDF)

S2 File The first documented timing for corneal clearing. The timing for corneal clearing varies widely among individual patients, as it depends on the severity and extent of the pre-operative opacity, as well as the severity of the ocular trauma. Given the retrospective nature of this cohort, follow-up visits were not scheduled at uniform intervals; thus the exact onset of corneal clearing could not be precisely determined for every eye, and only the timing of actual follow-up visits could be verified. These data should be interpreted cautiously.
(PDF)
