# Supplementary material for: Clinical course and outcomes of corneal blood staining in open-globe injury: Eye Injury Vitrectomy Study
Source: PLoS One. 2026 Aug 4;21(8):e0354967. doi: 10.1371/journal.pone.0354967 (PMC13436741; doi:10.1371/journal.pone.0354967)
Supplement: Supplementary file 2 [file pone.0354967.s002.pdf]

S1 Table The first documented timing for corneal clearing

		Months	N
**Overall**			
	Partial clearing, median (IQR)	3 (3- 7.5)	22
	Complete clearing, median (IQR)	24 (10- 30)	13
**Anterior chamber washout only**			
	Partial clearing, median (IQR)	3 (2- 4)	4
	Complete clearing, median (IQR)	13 (8-18)	4
**TKP + PPV + RTC**			
	Partial clearing, median (IQR)	4 (3- 9)	18
	Complete clearing, median (IQR)	26 (18- 32)	9

The timing for corneal clearing varies widely among individual patients, as it depends on the severity and extent of the pre-operative opacity, as well as the severity of the ocular trauma. Given the retrospective nature of this cohort, follow-up visits were not scheduled at uniform intervals; thus the exact onset of corneal clearing could not be precisely determined for every eye, and only the timing of actual follow-up visits could be verified. These data should be interpreted cautiously.
