# Supplementary material for: Clinical course and outcomes of corneal blood staining in open-globe injury: Eye Injury Vitrectomy Study
Source: PLoS One. 2026 Aug 4;21(8):e0354967. doi: 10.1371/journal.pone.0354967 (PMC13436741; doi:10.1371/journal.pone.0354967)
Supplement: Supplementary file 5 [file pone.0354967.s006.docx]

Reviewer #1: This article is generally well written and only requires minor revision. Some suggestions
Line 67 severe could be replaced by "the worst visual outcomes'
Line 113 , define what is an experience physician

Thank you very much for the positive evaluation of our manuscript and for these helpful suggestions.

For Line 67, we have revised the sentence accordingly. **(Line 49, Page 4)**

*“Open-globe injuries (OGIs) are ocular injuries associated with the worst visual outcomes and may result in monocular or bilateral blindness, particularly in eyes with globe rupture ”*

For the definition of “experience physician”. We agree that the term “experienced physician” was not sufficiently specific and could be misleading. We intended to indicate trained clinicians who were able to align serial AS-OCT scans by using anatomical landmarks and previous scan locations as references. We have revised the Methods to clarify this point. **(Line 96- 99, Page 6)**

*“…AS-OCT (Wavelength nanometer, Tomey CASIA2, Nagoya, Japan) was performed by trained clinicians (AD, HW, and SG), with serial scans were obtained from same corneal regions whenever possible, using anatomical landmarks and previous scan locations as references.”*

Please find the revisions **highlighted in red**.

Reviewer #2: Reviewer Comments
This manuscript addresses an uncommon but clinically relevant problem: the management of corneal blood staining in severe open-globe injury requiring posterior segment surgery. The authors present a conservative strategy in which anterior chamber washout is performed first, followed by TKP-assisted PPV with replacement of the trephined autologous cornea when visualization remains inadequate, while avoiding routine immediate allograft PKP. The topic is important, the case series is valuable for a relatively rare clinical scenario, and the photographic documentation is a strength. However, the manuscript requires major revision before it can be considered for publication. The main issues relate to incomplete characterization of cohort selection and follow-up, lack of a clearly defined corneal clearance outcome, limited analysis, underdeveloped AS-OCT reporting, and conclusions that are somewhat stronger than the data currently support.

Thank you for your comments.

Major comments
1. Study design and cohort selection need clearer reporting.
The manuscript states that this is a retrospective analysis of EIVS data, but it is unclear whether the 42 eyes represent a consecutive case series, a selected registry subset, or all eligible cases during a defined period (which was not stated). The authors should state the period during which the cases were selected, whether patients were consecutive, the number screened or excluded, and reasons for exclusion. A simple flow diagram would improve transparency and help readers assess selection bias and generalizability.

Thanks for this valuable suggestion.

This was a retrospective analysis of cases retrieved from the EIVS database between June 2012 and June 2025. As defined by the EIVS database, the general inclusion criteria were patients who suffered from severe eye injury and were treated with vitreoretinal surgery, enucleation, or evisceration [1].

A total of 3,152 eyes in the EIVS database were screened, including 1,920 eyes with open-globe injuries. Among them, 157 eyes had documented corneal blood staining. After excluding 115 eyes because of endophthalmitis, pre-existing ocular diseases, incomplete clinical or photographic records, follow-up shorter than 12 months, or enucleation/evisceration within 12 months; 42 eyes were included in the final analysis. We have added these details to the Methods and Results, and provided a flow diagram as **S1 Figure.**

Please find them highlighted in red **(Line 81-83, Page 5; Line 89- 90, Page 6; Line 124- 128, Page 7**)

**S1 Figure Flow diagram of the study population enrollment from the EIVS database.**

2. The central corneal outcome is not operationally defined.
The objective of this paper was to describe clinical course and outcome of corneal blood staining following the interventions. Yet, the paper’s main conclusion rests on “spontaneous clearance,” “corneal clarity,” and formation of a peripheral “window,” yet these outcomes are described narratively rather than systematically. The authors should define what constitutes sufficient clarity for PPV after washout, and how corneal status was graded clinically, photographically, or by AS-OCT, and whether grading was performed by one or more observers. A formal grading system or at least reproducible categories would make the conclusions more credible.

We appreciate this comment.

In the revised manuscript, we defined “sufficient clarity for PPV after anterior chamber washout” as intraoperative corneal clarity that allowed the surgeon to safely identify major posterior segment structures and proceed with PPV without the use of TKP. We acknowledge that this criterion inevitably contains some degree of clinical judgment, because the feasibility of PPV depends not only on the size and location of the clear corneal area, but also on the extent of posterior segment pathology, the presence of iris or blood clot obstruction, and the surgeon’s experience. In this study, PPV without TKP was attempted only when the operating surgeon (including KF, YL, HC, LH and ZZM) considered that a continuous, unobstructed corneal viewing area was sufficient to permit safe posterior segment visualization and surgical manipulation. Otherwise, TKP-assisted PPV was performed. **(Line 107- 113, Page 6 and 7, highlighted in red**)

We also added reproducible postoperative corneal outcome categories, including complete clearing, partial clearing, and persistent opacity. “Complete clearing” was defined as complete disappearance of opacity attributable to corneal blood staining during follow-up, although other non-blood-staining corneal changes, such as scarring or band keratopathy, could still be present. “Partial clearing” was defined as recognizable decrease of corneal blood staining compared with the preoperative status, with formation of a clear corneal area sufficient for clinical posterior segment assessment, including retinal status and intraocular tamponade status. “Persistent opacity” was defined as residual corneal opacity due to blood staining that showed little or no regression compared with the preoperative status and continued to limit corneal transparency or fundus visualization.

Serial slit-lamp photographs, fundus photographs when available, and clinical records were independently reviewed by two ophthalmologists (KF and LH) to classify the corneal outcomes. Disagreements were resolved by a senior ophthalmologist (ZZM). These definitions have been added to the Methods (**Line 114- 116, Page 7**), and the corresponding subgroup results have been summarized in the Results (**Line 140- 143, Page 8**) and Table 1.

3. Follow-up duration must be reported more adequately.
Since corneal blood staining clearance is inherently time-dependent, the actual follow-up duration of the cohort is essential. The manuscript should provide the median follow-up with IQR or range, and ideally also report time to partial clearance, time to complete clearance where achieved, and time to eventual PKP in the one transplanted eye.

Thank you for highlighting this issue. We agree that follow-up duration is essential for interpreting the natural course of corneal blood staining. Previous reports have shown substantial variability in the speed of spontaneous clearing. In traumatic hyphema-related corneal blood staining, partial clearing has been observed within weeks to months, whereas complete clearing may require 10- 15 months or even up to 2- 3 years in some cases [2-5]. This variability suggests that the course of clearance may be influenced by the severity of corneal blood staining, as well as the degree of damage to the corneal endothelium and stroma.

In our series, all eyes had trauma-related corneal blood staining and underwent anterior chamber washout during surgery; however, the degree of hyphema, preoperative IOP, interval from injury to surgery, and tamponade / anterior segment status varied among patients. These factors may have contributed to heterogeneity in the timing and extent of corneal clearing.

Accordingly, we have added the median follow-up duration of 36 months (IQR, 23- 42 months; range, 12- 60 months) to the Results (**Line 135- 136, Page 8**) and Table 1. We also reviewed the available clinical records and serial photographs and reported the first documented visit at which partial or complete clearing was observed (**S1 Table**). The timing for corneal clearing varies widely among individual patients, as it depends on the severity and extent of the pre-operative opacity , as well as the severity of the ocular trauma. Specifically, the median time to partial clearing was 3 months (IQR, 3- 7.5 months), and the median time to complete clearing was 24 months (IQR, 10- 30 months). Given the retrospective nature of this cohort, follow-up visits were not scheduled at uniform intervals; thus the exact onset of corneal clearing could not be precisely determined for every eye, and only the timing of actual follow-up visits could be verified. We have clarified this limitation in the revised manuscript. (**Page 15 and 16**)

For the single eye that underwent allograft PKP, we now state that PKP was performed 14 months after the initial surgery, together with the ocular status at that time (**Line 173- 183, Page 9 and 10**).

4. The results should more clearly describe what happened to each subgroup.
The manuscript reports that 8 eyes achieved adequate clarity after anterior chamber washout alone, while 34 required TKP + PPV + RTC, but the later corneal course of each subgroup is not fully summarized. The authors should state:

•how many eyes achieved complete versus partial clearing

•how many retained persistent central opacity or scarring

•how many developed band keratopathy

•whether all eyes eventually achieved a clinically useful fundus view,

•and whether corneal outcomes differed between the washout-only and TKP groups.
This has a tendency to further strengthen the clinical message.

Thank you for this suggestion. In the supplementary materials, we have added a subgroup analysis comparing the washout-only group and the TKP + PPV + RTC group. The numbers of eyes with complete clearing, partial clearing, and persistent opacity have been added to the Results (**Line 140- 144, Page 8**) and Table 1.

Persistent central opacity or scarring was observed in 17/42 eyes. In this cohort of severe OGIs, residual central opacity may have resulted from multiple factors in addition to corneal blood staining, including the original corneal wound, suture-related scarring, and band keratopathy. Final visual function was also strongly affected by lens loss and severe posterior segment injuries, such as retinal detachment, choroidal detachment, and retinal tissue loss. Therefore, the independent functional impact of central opacity could not be reliably isolated in this retrospective study.

Band keratopathy was observed in 9 of 42 eyes, all of which were tamponaded with silicone oil. Because band keratopathy has been associated with long-term silicone oil tamponade, aphakia (which may facilitate direct contact between silicone oil and the corneal endothelium), hypotony, repeated intraocular surgery, and chronic inflammation [6, 7], we did not interpret it as a direct consequence of corneal blood staining. Instead, it is reported as a postoperative corneal complication in the context of severe ocular trauma.

We further clarified whether a clinically useful fundus view was achieved in each subgroup, including eyes with complete and partial clearing. In anterior chamber washout group (n = 8), 4/8 achieved complete clearing, while 4/8 showed partial clearing. In the TKP + PPV + RTC group (n = 34), 9/34 exhibited complete clearing, 18/34 presented partial clearing, and 7/34 suffered from persistent opacity.

For eyes with corneal opacity that limited direct visualization, including during the early postoperative period (e.g., at 1 week), posterior segment assessment was performed using B-scan ultrasonography, visual function examinations, and IOP measurement. The decision to perform subsequent PKP is strictly based on a comprehensive evaluation of both visual function and the anatomical viability of the injured eye; otherwise, allogeneic PKP is not recommended. (**Line 209- 213, Page 12**)

The data suggested that eyes in the washout-only group generally demonstrated faster and more complete clearing of corneal blood staining (please find it in **S1 Table**). Given the limited sample size and baseline imbalance, these subgroup findings are presented descriptively.

5. AS-OCT is underdeveloped and should either be analyzed or toned down.
AS-OCT is included in the methods and highlighted in a representative figure, yet no cohort-level imaging findings are presented. If AS-OCT was routinely performed, the authors should summarize findings such as stromal hyperreflectivity, corneal thickness, and correlation with final corneal status. If it was not analyzable in a systematic way, then the discussion of AS-OCT’s prognostic value should be more cautious.

We agree with the reviewer. In the original manuscript, AS-OCT was overemphasized relative to the available cohort-level imaging data. Although AS-OCT was useful for documenting representative anatomical changes, imaging was not performed using a standardized quantitative protocol at fixed postoperative intervals in all eyes. Therefore, reliable cohort-level analysis of stromal hyperreflectivity, corneal thickness, or correlations with final corneal status was not feasible. In the revised manuscript, we have toned down the AS-OCT-related statements, retained AS-OCT only as representative images, and added the lack of standardized serial AS-OCT analysis as a limitation. (**Page 15 and 16**)

6. Visual outcome interpretation should be more cautious.
The manuscript reports statistically significant improvement in BCVA, but the cohort remains profoundly visually compromised overall, with many eyes presenting with NLP, many remaining NLP, and a proportion progressing to phthisis. The discussion and conclusion should avoid implying a strong functional benefit and should instead emphasize that this may be a globe-preserving and cornea-conserving strategy in eyes with otherwise poor prognosis.

We appreciate this comment. We agree that although BCVA showed statistical improvement, the cohort remained severely visually compromised overall, and the visual outcome was mainly limited by the severity of posterior segment injury. We have deleted this sentence in Result and revised the Discussion and Conclusion to avoid implying a strong functional benefit. The revised manuscript now emphasizes that this approach should be interpreted primarily as a globe-preserving and cornea-conserving strategy that may reduce exposure to prolonged postoperative corticosteroid or immunosuppressive therapy and lower the risk of graft rejection in eyes with limited visual potential. (**Page 13, 15, 16**)

7. Silicone oil status and IOP outcomes need deeper contextualization.
The paper notes that many eyes required additional silicone oil procedures and that only a few achieved complete oil removal. This is highly relevant because final IOP and eventual candidacy for corneal transplantation are likely influenced by recurrent posterior pathology, persistent hypotony, and the long-term graft-host environment. The manuscript should clarify how many eyes had secondary procedures, how many had oil refill, partial removal, or re-tamponade, whether final IOP was measured under oil in most eyes, and whether phthisical eyes were included in the IOP analysis.

Thank you for this suggestion. We agree that silicone oil status, IOP, and secondary procedures are important for interpreting final ocular status and later PKP candidacy.

In this cohort, all eyes were followed for at least 12 months. Among the 40 eyes initially tamponaded with silicone oil, only 4 achieved complete silicone oil removal by the final visit. Therefore, final IOP in most eyes was measured in the presence of silicone oil or partial silicone oil tamponade. The remaining eyes required one or more silicone oil-related secondary procedures during follow-up, including oil refill, partial oil removal, or oil removal with re-tamponade, mainly because of silicone oil emulsification, persistent hypotony, or recurrent posterior segment instability. Some eyes underwent more than one such procedure.

Silicone oil emulsification is a well-recognized complication of long-term tamponade, with previous studies reporting an increased risk when silicone oil remains in the eye for more than 6 months [8], an average onset of approximately 13 months [9], and very high rates after 12 months of tamponade [10]. Thus, in this cohort with follow-up of at least 12 months, repeated silicone oil-related procedures were not unexpected.

We also clarified that phthisis bulbi (n=5) were included in the overall anatomical outcome analysis. These findings further support our view that corneal transplantation candidacy in this cohort were strongly influenced by posterior segment status, tamponade condition, and overall ocular viability, rather than by corneal blood staining alone.

These findings suggest that many eyes remained in an unstable ocular condition, with persistent hypotony, long-term tamponade, or repeated posterior segment procedures, which may be unfavorable for graft survival after PKP.

8. The indication and context for the single allograft PKP should be stated explicitly.
Because only one eye underwent PKP, that case is clinically informative. The manuscript should state the indication, timing, tamponade status, ocular condition, and visual/anatomical rationale for proceeding with PKP. This would help define the authors’ threshold for delayed transplantation.

Thank you for this suggestion. We have revised the Results to provide additional details of the single case that underwent delayed allograft PKP. (**Line 173- 183, Page 9 and 10**)

*“Only one eye underwent delayed allograft PKP. This was a gas-tamponaded eye in a 46-year-old man with traumatic aniridia, aphakia after prior lens extraction in local hospital, vitreous hemorrhage, and post-traumatic secondary glaucoma. Although preoperative B-scan suggested choroidal detachment, this finding were not confirmed intraoperatively, and laser photocoagulation was applied around the retinal break after retinal attachment. The RTC gradually clearing, and BCVA reached 0.5 at 14 months after the initial surgery, with well-controlled IOP, indicating favorable visual potential and a relatively stable posterior segment. Allograft PKP combined with scleral-fixated IOL implantation was therefore performed for optical rehabilitation and aphakia correction. Early post-PKP follow-up was available, but the patient was subsequently lost to follow-up. When he returned 3 years after PKP, the eye had progressed to NLP due to glaucoma, with epithelial edema but relatively preserved stromal clarity in corneal graft.”*

9. The manuscript would benefit from association or predictor analyses.
The current analysis is mainly descriptive. Even in a modest sample, simple univariable or subgroup analyses would be useful. Potential comparisons include: washout-only versus TKP + PPV + RTC, complete versus incomplete corneal clearing, phthisis versus non-phthisis, final NLP versus retained light perception, eyes with and without useful fundus assessment, and associations with age, injury type (especially among the majority who had globe rupture), hypotony, retinal detachment, choroidal detachment, tamponade type, or no retina detected. This would strengthen the manuscript beyond a purely descriptive case series.

We appreciate this thoughtful suggestion. Given the small sample size and the highly imbalanced distribution of injury severity, including choroidal detachment, retinal detachment, retinal tissue loss, hypotony, and tamponade type, multivariable prediction modeling would be statistically underpowered and could lead to potentially misleading conclusions.

In this cohort of severe open-globe injuries, final visual and anatomical outcomes were largely determined by posterior segment damage and overall ocular viability. Therefore, the independent contribution of corneal blood staining to global outcomes, such as final BCVA, phthisis bulbi, or complete silicone oil removal, could not be reliably isolated in this retrospective dataset.

To provide additional clinical information while avoiding overinterpretation, we added an exploratory descriptive comparison between the washout-only group and the TKP + PPV + RTC group in **S1 Table and S2 Table**. The parameters included final corneal status, final BCVA, final IOP, and complete silicone oil removal. Eyes in the washout-only group tended to show faster and more complete clearance of corneal blood staining; however, this subgroup included only 8 eyes and likely represented eyes with less severe initial corneal opacity. Therefore, these findings are presented descriptively rather than as formal comparative or predictive analyses.

10. The strategy should be positioned more clearly relative to alternative approaches.
The discussion would be improved by explicitly comparing this approach with TKP plus immediate PKP, endoscopic vitrectomy, staged surgery, and observation before PPV where feasible. This would help readers understand when the proposed conservative approach is most appropriate in real-world practice.

Thank you for this comment. We respectfully emphasize that, in severe open-globe injuries with posterior segment pathology requiring PPV, observation for spontaneous corneal clearing should not be regarded as a clinically appropriate alternative.

Corneal blood staining may clear spontaneously, but this process can take months to years. In contrast, the timing of PPV after severe ocular trauma is critical. Delaying PPV increase the risk of proliferative vitreoretinopathy, which lead to irreversible anatomical or functional loss [11-13]. Therefore, the clinical question in these eyes is not whether to wait for corneal clearing before PPV, but how to obtain adequate posterior segment access while avoiding unnecessary high-risk allograft PKP in the acute traumatic setting.

Our strategy addresses this problem by performing anterior chamber washout first, followed by TKP-assisted PPV with replacement of the trephined autologous cornea when visualization remains inadequate. Compared with TKP followed by immediate allograft PKP, this approach avoids placing donor tissue into an acutely inflamed and often silicone-oil-filled eye. Although endoscopic vitrectomy can bypass corneal opacity, it is not yet a widely adopted standard approach in many centers and remains limited by a narrower field of view, lower image resolution, specialized equipment requirements, and a steep learning curve. These were discussed in the Introduction of original manuscript and we have revised them in Discussion to clarify this clinical positioning further. (**Page 15**)

Minor comments
1. Ethics approval inconsistency.
The ethics number differs between the submission material and the manuscript body and should be corrected.

Thank you for pointing this out. We apologize for the typo in the submission materials. The ethics approval number has been corrected.

2. Grammar and wording issues.
Several language issues need correction, including awkward or ungrammatical phrases.

Thank you for pointing this out. These have been corrected.

3. Table 1.
Table 1 would be more informative if it included follow-up duration, number of reoperations, timing of silicone oil removal/refill, corneal outcome categories, and final transparency status.

Thank you for pointing this out. We have expanded Table 1.

4. Discussion wording and internal consistency.
Some phrases in the discussion are awkward and should be revised for clarity. There also appears to be an incorrect figure reference when discussing the case with complete clearance (check inserted comments).

Thank you for pointing this out. We have revised them accordingly. Please find them highlighted in red.

5. References.
The reference list should be reviewed carefully for journal formatting and typographic consistency.

Sorry for the formatting and typographic inconsistency in the reference list. The references were now formatted using the 'Vancouver' style via EndNote, as recommended in the journal's submission guidelines. We also have manually checked and corrected the reference formatting for consistency and accuracy. Please let us know if any further changes are necessary. Thank you for pointing this out.

Overall recommendation
This is a clinically relevant manuscript with useful photographic documentation and an important practical message, especially in eyes with poor prognosis and limited donor-tissue justification. However, the paper currently needs substantial revision in outcome definition, follow-up reporting, and discussion balance before it is ready for publication.

1.	Feng, K., Y.T. Hu, and Z. Ma, *Prognostic indicators for no light perception after open-globe injury: eye injury vitrectomy study.* Am J Ophthalmol, 2011. **152**(4): p. 654-662.e2.

2.	Brodrick, J.D., *Corneal blood staining after hyphaema.* Br J Ophthalmol, 1972. **56**(8): p. 589-93.

3.	Yang, X., Q. Zhou, and S. Du, *Conjunctival flap covering in the treatment of corneal blood staining.* Can J Ophthalmol, 2011. **46**(5): p. 442-3.

4.	Lin, H.Y. and K.L. Peng, *Gradual spontaneous resolution of corneal bloodstaining after anterior chamber irrigation for rebleeding secondary to traumatic hyphema.* Int Med Case Rep J, 2018. **11**: p. 239-242.

5.	Hwang, B.E., et al., *Serial Photographic Monitoring of Spontaneous Clearance of Corneal Blood Stain in a Child with Traumatic Hyphema.* Korean J Ophthalmol, 2017. **31**(3): p. 281-282.

6.	He, K., et al., *Risk Factors for Band Keratopathy in Aphakic Eyes With Silicone Oil Tamponade for Open-Globe Injuries: A Multicenter Case-Control Study.* Front Med (Lausanne), 2021. **8**: p. 713599.

7.	Bennett, S.R. and G.W. Abrams, *Band keratopathy from emulsified silicone oil.* Arch Ophthalmol, 1990. **108**(10): p. 1387.

8.	Ni, Y., et al., *Analysis of the causative factors related to earlier emulsification of silicone oil.* Int J Ophthalmol, 2019. **12**(3): p. 517-519.

9.	Toklu, Y., et al., *Time course of silicone oil emulsification.* Retina, 2012. **32**(10): p. 2039-44.

10.	Federman, J.L. and H.D. Schubert, *Complications associated with the use of silicone oil in 150 eyes after retina-vitreous surgery.* Ophthalmology, 1988. **95**(7): p. 870-6.

11.	Ozdek, S., M. Hasanreisoglu, and E. Yuksel, *Chorioretinectomy for perforating eye injuries.* Eye, 2013. **27**(6): p. 722-727.

12.	Chauhan, M.Z., et al., *Outcomes of pars plana vitrectomy following ocular trauma at varying surgical time points.* International Journal of Retina and Vitreous, 2022. **8**(1): p. 49.

13.	Miller, S.C., et al., *Global Current Practice Patterns for the Management of Open Globe Injuries.* American Journal of Ophthalmology, 2022. **234**: p. 259-273.
