# Supplementary material for: Clinical course and outcomes of corneal blood staining in open-globe injury: Eye Injury Vitrectomy Study
Source: PLoS One. 2026 Aug 4;21(8):e0354967. doi: 10.1371/journal.pone.0354967 (PMC13436741; doi:10.1371/journal.pone.0354967)
Supplement: Supplementary file 6 [file pone.0354967.s003.docx]

PLOS One

**Clinical Course and Outcomes of Corneal Blood Staining in Open-Globe Injury: Eye Injury Vitrectomy Study**

--Manuscript Draft--

| **Manuscript Number:** | PONE-D-26-00677 |
| --- | --- |
| **Article Type:** | Research Article |
| **Full Title:** | Clinical Course and Outcomes of Corneal Blood Staining in Open-Globe Injury: Eye Injury Vitrectomy Study |
| **Short Title:** | Corneal Blood Staining in Open-Globe Injury: EIVS |
| **Corresponding Author:** | Zhi-zhong Ma  Peking University Third Hospital Beijing, CHINA |
| **Keywords:** | open-globe injury; corneal blood staining; temporary keratoprosthesis; penetrating keratoplasty; vitrectomy |
| **Abstract:** | Abstract  Purpose: To describe the clinical course and outcomes of corneal blood staining in open-globe injuries (OGIs) after vitrectomy without routine immediate allograft penetrating keratoplasty (PKP).  Methods: This study included 42 eyes of 42 patients with corneal blood staining secondary to OGIs. All eyes underwent anterior chamber washout at the start of pars plana vitrectomy (PPV). When visualization remained inadequate, temporary keratoprosthesis (TKP)-assisted PPV was performed, followed by replacement of the trephined autologous cornea (TKP + PPV + RTC). Postoperative outcomes, including corneal transparency, best-corrected visual acuity (BCVA), intraocular pressure (IOP), and need for subsequent PKP, were assessed.  Results: Median age was 36.5 years; globe rupture accounted for 95.2%. Preoperative hypotony (IOP ≤10 mmHg) was present in 90.5%, with severe posterior segment injury in all eyes. Eight eyes achieved sufficient corneal clarity after washout alone; the remaining 34 required TKP + PPV + RTC. During follow-up, varying degrees of spontaneous clearance occurred in all eyes, forming a peripheral “window” that enabled fundus assessment. At the final follow-up, BCVA improved significantly from median 4.00 logMAR preoperatively to 2.80 logMAR (p = 0.002), and median IOP rose from 5.00 to 10.00 mmHg (p = 0.002). Five eyes developed phthisis bulbi, four eyes achieved complete silicone oil removal, and only 1 eye underwent allograft PKP. Conclusions: In OGIs with corneal blood staining, spontaneous partial-to-complete clearance is common after PPV, often allowing postoperative fundus evaluation. These findings support a conservative corneal strategy with selective, rather than routine, PKP in the follow-up. |
| **Order of Authors:** | Bingjie Wang, M.D. |
|  | Kang Feng, M.D. |
|  | Yao Lu, M.D. |
|  | Huijin Chen, M.D. |
|  | Liang Han, M.D. |
|  | Aihua Ding |
|  | Haikun Wang |
|  | Shaofeng Gu |
|  | Zhi-zhong Ma, M.D. |
| **Additional Information:** |  |
| **Question** | **Response** |
| **Financial Disclosure** | The author(s) received no specific funding for this work. |

| Enter a financial disclosure statement that describes the sources of funding for the work included in this submission. Review the [submission guidelines](http://journals.plos.org/plosone/s/submission-guidelines#loc-financial-disclosure-statement) for detailed requirements. View published research articles from [*PLOS ONE*](https://journals.plos.org/plosone/) for specific examples.  This statement is required for submission and **will appear in the published article** if the submission is accepted. Please make sure it is accurate. |  |
| --- | --- |
| **Funded studies**  Enter a statement with the following details:   - Initials of the authors who received each award - Grant numbers awarded to each author - The full name of each funder - URL of each funder website - Did the sponsors or funders play any role in the study design, data collection and analysis, decision to publish, or preparation of the manuscript? |  |
| Did you receive funding for this work? |  |
| **Competing Interests**  Use the instructions below to enter a competing interest statement for this submission. On behalf of all authors, disclose any [competing interests](http://journals.plos.org/plosone/s/competing-interests) that could be perceived to bias this work—acknowledging all financial support and any other relevant financial or non-financial competing interests.  This statement is required for submission and **will appear in the published article** if the submission is accepted. Please make sure it is accurate and that any funding sources listed in your Funding Information later in the submission form are also declared in your Financial Disclosure statement. | The authors have declared that no competing interests exist. |

| View published research articles from  [*PLOS ONE*](http://journals.plos.org/plosone/) for specific examples. |  |
| --- | --- |
| **NO authors have competing interests**  Enter: *The authors have declared that no competing interests exist.*  **Authors with competing interests**  Enter competing interest details beginning with this statement:  *I have read the journal's policy and the authors of this manuscript have the following competing interests: [insert competing interests here]* |  |
| * typeset |  |
| **Ethics Statement**  Enter an ethics statement for this submission. This statement is required if the study involved:   - Human participants - Human specimens or tissue - Vertebrate animals or cephalopods - Vertebrate embryos or tissues - Field research   Write "N/A" if the submission does not require an ethics statement.  General guidance is provided below. Consult the [submission guidelines](http://journals.plos.org/plosone/s/submission-guidelines#loc-guidelines-for-specific-study-types) for detailed instructions. **Make sure that all information entered here is included in the Methods section of the manuscript.** | The study was approved by the Institutional Review Board of Peking University Third Hospital Medical Science Research Ethics Committee (Ethic No. IRB00006761-2012069) and conducted in accordance with the Declaration of Helsinki. Clinical data were retrospective collected from the Eye Injury Vitrectomy Study (EIVS). The trial was registered with the Chinese Clinical Trial Registry (ChiCTR; Number: ChiCTR-OCH-08000199) on November 28, 2008. |

| **Format for specific study types**  **Human Subject Research (involving human participants and/or tissue)**   - Give the name of the institutional review board or ethics committee that approved the study - Include the approval number and/or a statement indicating approval of this research - Indicate the form of consent obtained (written/oral) or the reason that consent was not obtained (e.g. the data were analyzed anonymously)   **Animal Research (involving vertebrate animals, embryos or tissues)**   - Provide the name of the Institutional Animal Care and Use Committee (IACUC) or other relevant ethics board that reviewed the study protocol, and indicate whether they approved this research or granted a formal waiver of ethical approval - Include an approval number if one was obtained - If the study involved *non-human primates*, add *additional details* about animal welfare and steps taken to ameliorate suffering - If anesthesia, euthanasia, or any kind of animal sacrifice is part of the study, include briefly which substances and/or methods were applied   **Field Research**  Include the following details if this study involves the collection of plant, animal, or other materials from a natural setting:   - Field permit number - Name of the institution or relevant body that granted permission |  |
| --- | --- |
|  |  |
| **Data Availability**  Authors are required to make all data underlying the findings described fully available, without restriction, and from the time of publication. PLOS allows rare exceptions to address legal and ethical concerns. See the [PLOS Data Policy](http://journals.plos.org/plosone/s/data-availability) and [FAQ](http://journals.plos.org/plosone/s/data-availability#loc-faqs-for-data-policy) for detailed information. | No - some restrictions will apply |

| A Data Availability Statement describing where the data can be found is required at submission. Your answers to this question constitute the Data Availability Statement and **will be published in the article**, if accepted. |  |
| --- | --- |
| **Important:** Stating ‘data available on request from the author’ is not sufficient. If your data are only available upon request, select ‘No’ for the first question and explain your exceptional situation in the text box. |  |
| Do the authors confirm that all data underlying the findings described in their manuscript are fully available without restriction? |  |
| **Describe where the data may be found in full sentences. If you are copying our sample text, replace any instances of XXX with the appropriate details.**   - If the data are **held or will be held in a public repository**, include URLs, accession numbers or DOIs. If this information will only be available after acceptance, indicate this by ticking the box below. For example: *All XXX files are available from the XXX database (accession number(s) XXX, XXX.*). - If the data are all contained **within the manuscript and/or Supporting Information files**, enter the following: *All relevant data are within the manuscript and its Supporting Information files.* - If neither of these applies but you are able to provide **details of access elsewhere**, with or without limitations, please do so. For example:   *Data cannot be shared publicly because of [XXX]. Data are available from the XXX Institutional Data Access / Ethics Committee (contact via XXX) for researchers who meet the criteria for access to confidential data.*  *The data underlying the results presented in the study are available from (include the name of the third party* | Data cannot be shared publicly because they involve clinical information and are subject to restrictions imposed by the Institutional Review Board of Peking University Third Hospital Medical Science Research Ethics Committee . Data are available from the Ethics Committee (contact via [puh3_yk@bjmu.edu.cn](mailto:puh3_yk@bjmu.edu.cn); requests will be forwarded to the Committee) for researchers who meet the criteria for access to confidential data. |

| *and contact information or URL).*   - This text is appropriate if the data are owned by a third party and authors do not have permission to share the data.   * typeset |  |
| --- | --- |
| Additional data availability information: |  |

<https://www2.cloud.editorialmanager.com/pone/download.aspx?id=43419075&guid=cddb090e-0d0b-4072-af0f-59c8a2833b0b&scheme=1>Manuscript	[Click here to access/download;Manuscript;Corneal Blood Staining.v1.5.docx](https://www2.cloud.editorialmanager.com/pone/download.aspx?id=43419075&guid=cddb090e-0d0b-4072-af0f-59c8a2833b0b&scheme=1)

1 **Clinical Course and Outcomes of Corneal Blood Staining in Open-Globe Injury: Eye Injury**
2 **Vitrectomy Study**
3 Bingjie Wang, M.D.^1,2^, Kang Feng, M.D.^1^, Yao Lu, M.D.^1^, Huijin Chen^1^, M.D., Liang Han,
4 M.D.^1^, Aihua Ding, M.D.^1^, Haikun Wang, M.D.^1^, Shaofeng Gu, M.D. ^1^, Zhizhong Ma, M.D.^1*^
5 ^1^Department of Ophthalmology, Peking University Third Hospital, Beijing, China
6 ^2^Eye Center, Beijing Tsinghua Changgung Hospital, School of Clinical Medicine, Tsinghua
7 University, Beijing, China

8

9 Bingjie Wang, M.D. : [bingjiew29@gmail.com](mailto:bingjiew29@gmail.com)
10 Kang Feng, M.D. : [ocufeng@hsc.pku.edu.cn](mailto:ocufeng@hsc.pku.edu.cn)
11 Yao Lu, M.D. : [delichoc@sina.com](mailto:delichoc@sina.com)
12 Huijin Chen, M.D. : [chjdoc@aliyun.com](mailto:chjdoc@aliyun.com)
13 Liang Han, M.D. : [drhanliang@163.com](mailto:drhanliang@163.com)
14 Aihua Ding, M.D. : [thyrsa_ding@163.com](mailto:thyrsa_ding@163.com)
15 Haikun Wang, M.D. : [419034559@qq.com](mailto:419034559@qq.com)
16 Shaofeng Gu, M.D.: [gusf97@163.com](mailto:gusf97@163.com)
17 *Correspondence: Zhizhong Ma, M.D. :	[puh3_yk@bjmu.edu.cn](mailto:puh3_yk@bjmu.edu.cn)
18 49# HuaYuan Rd, HaiDian District, Beijing, China, 100191

19	+86-13801105509 office

20

21 ZZM conceived the study and developed the surgical approach. ZZM, YL, HC, KF, and LH
22 performed the surgical procedures. BW conducted data analysis and drafted the manuscript.
23 BW, AD, HW, and SG contributed to data collection. All authors reviewed and approved the
24 final version of the manuscript.

25

## 26 Key message:

27 What is known
28 1. Corneal blood staining in open-globe injuries traditionally requires immediate allograft penetrating
29 keratoplasty (PKP) to enable posterior segment access.
30 2. Immediate PKP in the acute phase is high-risk due to intense inflammation, hypotony, and long-term
31 silicone oil tamponade.
32 What is new
33 1. Blood-stained corneas tend to clear spontaneously; replacement of the trephined cornea allows for this
34 recovery, often creating a "window" sufficient for postoperative fundus evaluation.
35 2. Avoiding immediate allograft PKP prevents premature surgery in severely damaged eyes with poor
36 anatomical and visual prognoses.
37 3. Delaying transplantation until the ocular environment stabilizes reduces rejection risk and enables a
38 more evidence-based selection of the transplant type.

39

## 40 Abstract

41 Purpose: To describe the clinical course and outcomes of corneal blood staining in open-globe injuries
42 (OGIs) after vitrectomy without routine immediate allograft penetrating keratoplasty (PKP).
43 Methods: This study included 42 eyes of 42 patients with corneal blood staining secondary to OGIs. All
44 eyes underwent anterior chamber washout at the start of pars plana vitrectomy (PPV). When visualization
45 remained inadequate, temporary keratoprosthesis (TKP)-assisted PPV was performed, followed by
46 replacement of the trephined autologous cornea (TKP + PPV + RTC). Postoperative outcomes, including
47 corneal transparency, best-corrected visual acuity (BCVA), intraocular pressure (IOP), and need for
48 subsequent PKP, were assessed.
49 Results: Median age was 36.5 years; globe rupture accounted for 95.2%. Preoperative hypotony (IOP
50 ≤10 mmHg) was present in 90.5%, with severe posterior segment injury in all eyes. Eight eyes achieved
51 sufficient corneal clarity after washout alone; the remaining 34 required TKP + PPV + RTC. During
52 follow-up, varying degrees of spontaneous clearance occurred in all eyes, forming a peripheral “window”
53 that enabled fundus assessment. At the final follow-up, BCVA improved significantly from median 4.00
54 logMAR preoperatively to 2.80 logMAR (p = 0.002), and median IOP rose from 5.00 to 10.00 mmHg (p

55	= 0.002). Five eyes developed phthisis bulbi, four eyes achieved complete silicone oil removal, and only

56 1 eye underwent allograft PKP.
57 Conclusions: In OGIs with corneal blood staining, spontaneous partial-to-complete clearance is common
58 after PPV, often allowing postoperative fundus evaluation. These findings support a conservative corneal
59 strategy with selective, rather than routine, PKP in the follow-up.
60 **Keywords:** open-globe injury, corneal blood staining, temporary keratoprosthesis, penetrating
61 keratoplasty, vitrectomy

## 62 Statements and Declarations

63 There are no financial conflicts of interest to disclose. The manuscript has not been published and is not
64 under consideration elsewhere. All authors have approved the submission.

65

## 66 Introduction

67 Open-globe injuries (OGIs) represent one of the most severe forms of ocular trauma and may result in
68 monocular or bilateral blindness, with particularly poor visual prognosis in eyes with globe rupture [1,
69 2]. Adequate corneal transparency is essential for visualization of posterior segment pathology. In severe
70 ocular trauma, however, corneal transparency may be compromised, limiting clinical assessment and
71 impeding surgical repair.
72 Among trauma-related corneal opacities, corneal blood staining is one of the most challenging conditions.
73 It is characterized by deposition of hemoglobin and its breakdown products within the corneal stroma
74 [3], most commonly following prolonged hyphema and elevation of intraocular pressure (IOP) [4, 5],
75 but it has also been reported in eyes with low IOP [6, 7]. Disruption of endothelial function, stromal
76 impregnation by erythrocyte derivatives, and impaired metabolic clearance contribute to its development
77 [8].
78 In eyes with corneal blood staining requiring pars plana vitrectomy (PPV), a temporary keratoprosthesis
79 (TKP) is commonly used to facilitate posterior segment visualization [9]. This approach, however,
80 necessitates donor tissue for subsequent allograft penetrating keratoplasty (PKP) and is associated with
81 risks of graft rejection and infection related to long-term immunosuppression [10]. Furthermore, given
82 the poor prognosis in eyes with extensive posterior segment damage, the indication for immediate PKP
83 may be uncertain. Although endoscopy-assisted vitrectomy has been proposed as an alternative, its
84 adoption is limited by low image resolution, restricted field of view, specialized equipment requirements,
85 and a steep learning curve [11-13].
86 In this study, we evaluated a modified corneal conservative surgical strategy for OGIs complicated by
87 corneal blood staining, and analyzed anatomical and functional outcomes, as well as the evolution of
88 corneal blood staining.

## 89 Methods

90 **Human ethics and Consent to Participate declarations**
91 This study was approved by the Institutional Review Board of Peking University Third Hospital Medical
92 Science Research Ethics Committee (Ethics No. IRB00006761-2012060) and was conducted in
93 accordance with the Declaration of Helsinki. Clinical data were collected from the Eye Injury Vitrectomy
94 Study (EIVS). The trial was registered with the Chinese Clinical Trial Registry (ChiCTR; Number:
95 ChiCTR-OCH-08000199) on November 28, 2008. Written informed consent was obtained from all
96 participants at the time of enrollment in the EIVS, including consent for the use of non-personally
97 identifiable medical records for future research. For participants younger than 18 years of age, written
98 informed consent was obtained from their parents or legal guardians. The present study represents a
99 retrospective analysis of EIVS data from Peking University Third Hospital. Data were accessed for
100 research purposes on June 15, 2025. Throughout data collection and analysis, the investigators had access
101 only to fully anonymized data and no personal identifiable information. Given the retrospective nature
102 of this study and the use of anonymized data, the ethics committee waived the requirement for additional
103 informed consent.

## 104 Participants and Clinical Evaluation

105 Patients with OGIs complicated by corneal blood staining were included. Exclusion criteria were pre-
106 existing ocular disease, follow-up <12 months, or incomplete clinical records.
107 Preoperative data collected included age, sex, ocular history, best-corrected visual acuity (BCVA), IOP,
108 slit-lamp photographs, and anterior segment optical coherence tomography (AS-OCT). BCVA was
109 measured using a standard vision chart and converted into logarithm of the minimum angle of resolution
110 (logMAR). For statistical consistency, counting fingers, hand motion, light perception, and no light
111 perception (NLP) were converted to 1.87, 2.30, 2.80, and 4.00 logMAR, respectively. IOP was measured
112 using a rebound tonometer (iCare TA01i, Icare Finland Oy, Helsinki, Finland), AS-OCT (Wavelength
113 nanometer, Tomey CASIA2, Nagoya, Japan) was performed by experienced clinicians to evaluate the
114 extent of the corneal blood staining.
115 All patients underwent anterior chamber washout at the start of the initial PPV, followed by TKP-assisted
116 PPV with replacement of the trephined cornea (TKP + PPV + RTC) when necessary. The Surgical
117 Procedures of TKP + PPV + RTC was described previously [14]. Follow-up duration varied among
118 patients. BCVA, IOP, slit-lamp photographs, and AS-OCT were recorded at each visit. Additional
119 treatments were provided as clinically indicated.

## 120 Statistical analyses

121 Data were analyzed using SPSS software (version 26.0, IBM Corp., Armonk, NY, USA) and GraphPad
122 Prism 8 (GraphPad Software, San Diego, CA, USA). Descriptive statistics were reported as median
123 (interquartile range, IQR) for non-normally distributed variables or mean ± standard deviation for
124 normally distributed variables. Preoperative and postoperative parameters were compared using the
125 Wilcoxon matched-pairs signed rank test. A p-value of < 0.05 was considered statistically significant.

## 126 Results

127 A total of 42 patients (42 eyes; 16 right eyes) with corneal blood staining were included; 37 patients
128 (88.1%) were male. The median age was 36.5 years (range, 3–61 years). The mechanism of injury was
129 globe rupture in 40 eyes (95.2%), penetrating injury in 1 eye (2.4%), and intraocular foreign body (IOFB)
130 in 1 eye (2.4%). The median interval from trauma to surgery was 21.5 days (IQR, 15.0–28.5 days).
131 Preoperative ocular hypotony (intraocular pressure [IOP] ≤10 mmHg) was present in 38 eyes (90.5%).
132 Severe posterior segment injury was common: choroidal detachment occurred in 40 eyes, retinal
133 detachment in 37 eyes, and no identifiable retinal tissue was noted in 5 eyes. All eyes received intraocular
134 tamponade with silicone oil (n = 40) or long-acting gas (n = 2). During follow-up, many eyes required
135 additional silicone oil procedures (e.g., oil refill, partial removal, or removal with re-tamponade), and
136 complete silicone oil removal was achieved in only 4 eyes by the final visit.
137 After anterior chamber washout, adequate corneal clarity to proceed with PPV was achieved in 8 eyes,
138 whereas the remaining 34 eyes required TKP + PPV + RTC. Among eyes treated with TKP + PPV + RTC,
139 corneal blood staining showed varying degrees of resolution, forming a peripheral “window” that
140 permitted fundus assessment (Figure 1a–e). Some eyes subsequently developed band keratopathy and/or
141 corneal scarring (Figure 1). In all 8 eyes undergoing PPV after anterior chamber washout without TKP,
142 corneal clearing was observed during follow-up. Figure 2 presents serial AS-OCT images of one
143 representative case managed without TKP; the cornea progressively cleared and ultimately achieved
144 complete transparency.
145 Visual acuity improved postoperatively. Preoperatively, 28 eyes (66.7%) had NLP, which decreased to
146 12 eyes (28.6%) after surgery. At the final follow-up, phthisis bulbi developed in 5 eyes. Only 1 eye
147 (tamponaded with gas) underwent allograft PKP at the final visit. Baseline characteristics and
148 perioperative outcomes are summarized in Table 1.
149 Median BCVA improved from 4.00 logMAR preoperatively (IQR, 2.80–4.00) to 3.40 logMAR on day 1
150 (IQR, 2.80–4.00; p = 0.016) and 2.80 logMAR on day 7 (IQR, 2.80–4.00; p = 0.002), as well as at the
151 final visit (median, 2.80 logMAR; IQR, 2.30–4.00; p = 0.002). BCVA showed no significant difference
152 between day 7 and the final visit (p = 0.344) (Figure 3A).
153 Median IOP increased from 5.00 mmHg preoperatively (IQR, 4.00–8.75) to 8.95 mmHg on day 1 (IQR,
154 5.50–10.00; p = 0.019), measured 7.00 mmHg on day 7 (IQR, 6.25–10.00, p = 0.401), and reached 10.00
155 mmHg at the final visit (IQR, 5.50–11.00, p = 0.002) (Figure 3B).

## 156 Discussion

157 Management of corneal blood staining in the setting of complex ocular trauma remains challenging. In
158 this series, we applied a modified approach (anterior chamber washout first, then TKP + PPV + RTC if
159 needed) instead of performing immediate allogeneic PKP. A key observation was that corneal blood
160 staining frequently showed spontaneous partial-to-complete resolution after surgery, often providing a
161 peripheral “window” that permitted postoperative fundus assessment (Figure 1a–e). Only one eye
162 ultimately underwent PKP. These findings support a conservative corneal strategy in eyes with limited
163 visual potential and high graft-risk profiles. Conventionally, surgery has relied on immediate allogeneic
164 PKP following TKP–assisted PPV. However, this strategy exposes the corneal graft to pathophysiologic
165 stressors that are common in severely injured eyes.
166 Severe ocular trauma often leads to persistent hypotony and silicone-oil sustained [14, 15]. In OGIs,
167 hypotony can arise from direct ciliary body injury, cyclodialysis, ischemia and/or membrane formation
168 of ciliary body, leading to reduced aqueous humor production and/or increased outflow through the
169 suprachoroidal space [16-18]. Under these conditions, impaired aqueous circulation compromises
170 corneal metabolic support, while sustained contact between silicone oil and the corneal endothelium
171 accelerates endothelial dysfunction [19]. Together, these factors increase the risk of graft decompensation
172 and failure after PKP [20].
173 Inflammation is another problem. Eyes with OGIs are commonly in an active inflammatory state at the
174 time of PPV, which is a well-established independent risk factor for graft failure [21]. Inflammation
175 induces upregulation of major histocompatibility complex (MHC) class I and class II antigen expression
176 [22] and promotes vascular endothelial growth factor production, thereby facilitating graft rejection and
177 corneal neovascularization [23]. Consistent with these mechanisms, Williams et al. showed that past
178 ocular inflammation increased the risk of graft failure by 4.40-fold, while active inflammation increased
179 the risk to 9.6-fold [24]. Accordingly, delaying PKP until inflammation has stabilized may reduce the
180 risk of failure. The clinical importance of PKP timing has been supported by prior studies; for example,
181 Roters et al. reported a significantly lower rate of graft failure when PKP was performed after 8 weeks
182 of trauma [20]. Although delaying surgery may allow neovascularization to develop, as shown in Figure
183 1a, resulting in a vascularized graft bed. This trade-off may be acceptable, as the associated risk (odds
184 ratio, 2.74 [24]) is substantially lower than that posed by active inflammation.
185 Importantly, the overall prognosis of OGIs is often poor [14]. All eyes in our series had severe vision-
186 threatening pathology, including choroidal detachment, retinal detachment, or retinal tissue loss. Twenty-
187 eight eyes presented with no light perception (NLP), and 12 remained NLP after surgery. During follow-
188 up, 11.9% progressed to phthisis, indicating irreversible anatomical failure. In addition, silicone oil
189 removal, which is critical for graft survival, was achieved in only 10% of our cases. Under these
190 conditions, corneal transplantation may offer limited functional benefit in many eyes. Other global
191 assessment of ocular viability—such as IOP trends, residual light perception, and B scan findings—may
192 be more informative in guiding management decisions [25-27]. Consistent with this reality, only one eye
193 underwent PKP in our study, suggesting that most patients were poor candidates for primary keratoplasty;
194 for these individuals, TKP + PPV + RTC represents a more cost-effective and practical strategy. This
195 selective approach is also vital when considering the critical global shortage of donor tissue, where only
196 one cornea is available for every 70 patients in need [28].
197 The natural course of corneal blood staining further supports a conservative surgical strategy. Several
198 case reports have described spontaneous resolution of corneal blood staining [29, 30]. Clearance typically
199 progresses from the peripheral cornea toward the center and from posterior to anterior layers [30],
200 consistent with our observations. This pattern has been attributed to limbal circulation and shedding of
201 iron-containing epithelial cells [8]. Conjunctival flap coverage may further accelerate this process [31],
202 possibly by increasing local blood supply. In our series, all eyes showed varying degrees of spontaneous
203 clearance during follow-up, which enabled posterior segment assessment through this window. Figure 3
204 illustrates one case with complete clearance, the AS-OCT result showed a progressive reduction and
205 eventual disappearance of hyperreflective stromal opacity. Serial AS-OCT may also provide prognostic
206 value. Specifically, loss of hyperreflectivity of Descemet’s membrane has been proposed as a marker of
207 irreversible Descemet–endothelial complex injury, which may indicate the need for endothelial
208 keratoplasty [7]. Besides, many other eyes developed central band keratopathy after clearance as shown
209 in Figure 1f, for which lamellar keratoplasty is typically sufficient [32].

## 210 Limitations

211 This study is limited by its retrospective analysis and the heterogeneity of severe ocular trauma, including
212 variability in injury mechanism, posterior segment pathology, tamponade status, and follow-up duration.
213 Larger, prospective studies with standardized imaging and clinical grading are needed to better define
214 predictors of spontaneous clearance and to refine timing and selection of subsequent keratoplasty.

## 215 Conclusion

216 This conservative corneal strategy—beginning with anterior chamber washout and proceeding to TKP +
217 PPV + RTC when needed—often allowed spontaneous corneal clearance and enabled postoperative
218 fundus assessment. These findings support selective, rather than routine, PKP in OGIs complicated by
219 corneal blood staining.

## 220 Acknowledgement

221 This project has no funding support.

## 222 Conflict of Interest:

223 None of the authors have any conflicting interests to disclose.

## 224 Author Contribution Statement:

225 ZZM conceived the study and developed the surgical approach. ZZM, YL, HC, KF, and LH performed
226 the surgical procedures. BW conducted data analysis and drafted the manuscript. BW, AD, HW, and SG
227 contributed to data collection. All authors reviewed and approved the final version of the manuscript.

## 228 Reference

229 1.	Colyer, M.H., et al., *Perforating globe injuries during operation Iraqi Freedom.*

230	Ophthalmology, 2008. **115**(11): p. 2087-2093. e2.

231	2.	Miller, S.C., et al., *Global Current Practice Patterns for the Management of Open Globe*

232	*Injuries.* American Journal of Ophthalmology, 2022. **234**: p. 259-273.

233	3.	Ryan, D.S., et al., *Anterior segment imaging in combat ocular trauma.* J Ophthalmol, 2013.

234	**2013**: p. 308259.

235	4.	McDonnell, P.J., et al., *Fluorescence of blood-stained cornea.* Cornea, 1991. **10**(5): p. 445-

236	9.

237	5.	Brodrick, J.D., *Corneal blood staining after hyphaema.* Br J Ophthalmol, 1972. **56**(8): p.

238	589-93.

239	6.	Beyer, T.L. and L.W. Hirst, *Corneal blood staining at low pressures.* Arch Ophthalmol, 1985.

240	**103**(5): p. 654-5.

241 7.	Mansoori, T. and A. Srirampur, *Anterior Segment Optical Coherence Tomography*
242 *Findings in Corneal Blood Staining.* J Curr Ophthalmol, 2021. **33**(1): p. 95.
243 8.	McDonnell, P.J., et al., *Blood staining of the cornea. Light microscopic and ultrastructural*

244	*features.* Ophthalmology, 1985. **92**(12): p. 1668-74.

245 9.	Guevara-Villarreal, D.A. and P.J. Rodrjguez-Valdés, *Posterior Segment Intraocular Foreign*

# 246 Body: Extraction Surgical Techniques, Timing, and Indications for Vitrectomy. J

Ophthalmol, 2016. **2016**: p. 2034509.

| 247 |  |
| --- | --- |
| 248 | 10. |
| 249 |  |
| 250 | 11. |
| 251 |  |
| 252 | 12. |
| 253 |  |
| 254 | 13. |
| 255 |  |
| 256 | 14. |
| 257 |  |
| 258 |  |
| 259 | 15. |
| 260 |  |
| 261 |  |
| 262 | 16. |
| 263 |  |
| 264 | 17. |
| 265 |  |
| 266 | 18. |
| 267 |  |
| 268 |  |
| 269 | 19. |
| 270 |  |
| 271 | 20. |
| 272 |  |
| 273 | 21. |
| 274 |  |
| 275 | 22. |
| 276 |  |
| 277 | 23. |
| 278 |  |
| 279 |  |
| 280 | 24. |
| 281 |  |
| 282 | 25. |
| 283 |  |
| 284 | 26. |
| 285 |  |
| 286 | 27. |
| 287 |  |
| 288 | 28. |
| 289 |  |
| 290 | 29. |

Fasolo, A., et al., *Risk factors for graft failure after penetrating keratoplasty: 5-year follow-up from the corneal transplant epidemiological study.* Cornea, 2011. **30**(12): p. 1328-35. Dave, V.P., et al., *Intraocular endoscopy: A review.* Indian J Ophthalmol, 2021. **69**(1): p. 14-25.

Ajlan, R.S., A.A. Desai, and M.A. Mainster, *Endoscopic vitreoretinal surgery: principles, applications and new directions.* Int J Retina Vitreous, 2019. **5**: p. 15.

Abouodah, H., et al., *Endoscopic Pars Plana Vitrectomy in Patients With Open-Globe Injury and Corneal Opacity.* J Vitreoretin Dis, 2023. **7**(2): p. 103-108.

Chen, H.J., et al., *Anatomical outcome of vitreoretinal surgery using temporary keratoprosthesis and replacement of the trephined corneal button for severe open globe injuries: one-year result.* J Ophthalmol, 2014. **2014**: p. 794039.

Pfister, N., et al., *LONG-TERM MICROVASCULAR REMODELING AND CYSTIC CHANGES AFTER RETINAL DETACHMENT TREATED WITH SILICON OIL TAMPONADE.* Retina, 2023. **43**(6): p. 923-931.

Chen, B., et al., *Scleral buckling combined with internal cyclopexy for severe traumatic cyclodialysis cleft in open globe injuries.* Int J Ophthalmol, 2019. **12**(10): p. 1649-1653.

Rizzo, S., et al., *Hypotony and the Argus II retinal prosthesis: causes, prevention and management.* Br J Ophthalmol, 2020. **104**(4): p. 518-523.

Faude, F. and P. Wiedemann, *Vitreoretinal endoscope for the assessment of the peripheral retina and the ciliary body after large retinectomies in severe anterior PVR.* Int Ophthalmol, 2004. **25**(1): p. 53-6.

Yang, C.S., et al., *Cytotoxicity of silicone oil on cultivated human corneal endothelium.* Eye, 2008. **22**(2): p. 282-288.

Roters, S., et al., *Outcome of combined penetrating keratoplasty with vitreoretinal surgery for management of severe ocular injuries.* Retina, 2003. **23**(1): p. 48-56.

Coster, D.J. and K.A. Williams, *The impact of corneal allograft rejection on the long-term outcome of corneal transplantation.* Am J Ophthalmol, 2005. **140**(6): p. 1112-22.

Pepose, J.S., et al., *Detection of HLA class I and II antigens in rejected human corneal allografts.* Ophthalmology, 1985. **92**(11): p. 1480-4.

Cursiefen, C., et al., *VEGF-A stimulates lymphangiogenesis and hemangiogenesis in inflammatory neovascularization via macrophage recruitment.* The Journal of clinical investigation, 2004. **113**(7): p. 1040-1050.

Williams, K.A., et al., *Factors predictive of corneal graft survival. Report from the Australian Corneal Graft Registry.* Ophthalmology, 1992. **99**(3): p. 403-14.

Zhou, Y., et al., *Open Globe Injuries: Review of Evaluation, Management, and Surgical Pearls.* Clin Ophthalmol, 2022. **16**: p. 2545-2559.

Kylstra, J.A., J.C. Lamkin, and D.K. Runyan, *Clinical Predictors of Scleral Rupture After Blunt Ocular Trauma.* American Journal of Ophthalmology, 1993. **115**(4): p. 530-535.

Ding, C. and J. Zeng, *Clinical study on Hypotony following blunt ocular trauma.* Int J Ophthalmol, 2012. **5**(6): p. 771-3.

Gain, P., et al., *Global Survey of Corneal Transplantation and Eye Banking.* JAMA Ophthalmology, 2016. **134**(2): p. 167-173.

Hwang, B.E., et al., *Serial Photographic Monitoring of Spontaneous Clearance of Corneal*

291 *Blood Stain in a Child with Traumatic Hyphema.* Korean J Ophthalmol, 2017. **31**(3): p. 281-
292 282.
293 30.	Lin, H.Y. and K.L. Peng, *Gradual spontaneous resolution of corneal bloodstaining after*

# 294 anterior chamber irrigation for rebleeding secondary to traumatic hyphema. Int Med Case

295 Rep J, 2018. **11**: p. 239-242.
296 31.	Yang, X., Q. Zhou, and S. Du, *Conjunctival flap covering in the treatment of corneal blood*
297 *staining.* Can J Ophthalmol, 2011. **46**(5): p. 442-3.
298 32.	Kwon, Y.S., Y.S. Song, and J.C. Kim, *New treatment for band keratopathy: superficial*

# 299 lamellar keratectomy, EDTA chelation and amniotic membrane transplantation. J Korean

300 Med Sci, 2004. **19**(4): p. 611-5.

301

302

303

## 304 Table 1 Patient demographics, preoperative/postoperative ocular findings

Patients Enrolled (n = 42)

Age, median (IQR)	(years)	36.5 (25.8~ 49.3)

| Right eye, n (%) |  | 16 (38.1) |
| --- | --- | --- |
| Male, n (%) |  | 37 (88.1) |
| Surgery time interval, median (IQR) | (days) | 21.5 (15.0~ 28.5) |
| Open globe injury, n (%) |  | 42 (100) |
| Rupture | | 40 (95.2) |
| Penetrating | | 1 (2.4) |
| Intraocular foreign bodies (IOFB) | | 1 (2.4) |
| Hypotony, n (%) | | 38 (90.5) |
| Retinal and choroidal condition, n (%) | |  |
| Choroidal detachment | | 40 (95.2) |
| Retinal detachment | | 37 (88.1) |
| No retina detected | | 5 (11.9) |
| Intraocular tamponade, n (%) | | 42 (100) |
| Silicone oil | | 40 (95.2) |
| Long-acting gas | | 2 (4.8) |
| Balanced salt solution | | 0 (0) |

Cornea management, n (%)

TKP + PPV + RTC	34 (81.0)

After anterior chamber washout, corneal

transparency was sufficient for PPV

8 (19.0)

|  | NLP preoperatively, n (%) | 28 (66.7) |
| --- | --- | --- |
|  | NLP postoperatively, n (%) | 12 (28.6) |
|  | Phthisis bulbi at the final follow-up, n (%) | 5 (11.9) |
|  | SOR at the final follow-up, n (%) | 4/40 (10) |
|  | Received PKP at the final follow-up, n (%) | 1 (2.4), tamponade with gas |
| 305 |  |  |
| 306 |  |  |

307

## 308 Figure 1. Anterior segment photographs of corneal blood staining before and after clearing, with

309 **corresponding fundus views.**
310 This series focuses on corneal changes and the possible for fundus assessment; concomitant intraocular
311 comorbidities were not considered. Each case is presented as paired panels, with the corresponding
312 fundus image displayed in the upper right corner (a–e).
313 (A, a) Immediate postoperative and postoperative month 6 (history of radial keratotomy). Vascularization
314 at periphery cornea can be seen.
315 (B, b) Postoperative months 2 and 17.
316 (C, c) Intraoperative and postoperative month 12.
317 (D, d) Postoperative months 6 and 24.
318 (E, e) Intraoperative and postoperative month 48.
319 (F, f) Preoperative and postoperative month 7, showing band keratopathy.
320 (G, g) Preoperative and postoperative day 1.
321 (H, h) Immediate postoperative and postoperative month 24, showing central corneal opacity.
322 (I, i) Postoperative day 1 and postoperative month 15.
323 (J, j) Preoperative and postoperative month 4.

324

325

326

## Figure 2. Slit-lamp photographs and AS-OCT demonstrating complete resolution of corneal blood

327	**staining.**

328

329

(A, D) Postoperative month 4; (B, E) postoperative month 10; (C, F) postoperative month 16.

330

## 331 Figure 3. Changes in BCVA and IOP preoperative/postoperative.

332 (A) BCVA demonstrated significant improvement at D1, D7 and the final visit comparing with
333 preoperative. No significant improvement was observed at final visit when compared with D7 (n = 42).
334 (B) IOP significantly increased at D1, and dropped at D7 to preoperative level. At final visit, IOP showed
335 significant increase compared with pre-op, this process includes multiple silicone oil refilling in some
336 cases.
337 Median with IQR, Wilcoxon matched-pairs signed-rank test; *p < 0.05, **p < 0.01.
338 Abbreviations: Pre-op, preoperative; D1, postoperative day 1; D7, postoperative day 7.

339

340

Figure	[Click here to access/download;Figure;Figure 1.png](https://www2.cloud.editorialmanager.com/pone/download.aspx?id=43230351&guid=59f4ce05-e740-4f5b-8d85-574bbba2b4f8&scheme=1)[<https://www2.cloud.editorialmanager.com/pone/download.aspx?id=43230351&guid=59f4ce05-e740-4f5b-8d85-574bbba2b4f8&scheme=1>](https://www2.cloud.editorialmanager.com/pone/download.aspx?id=43230351&guid=59f4ce05-e740-4f5b-8d85-574bbba2b4f8&scheme=1)

Figure	[Click here to access/download;Figure;Figure 2.png](https://www2.cloud.editorialmanager.com/pone/download.aspx?id=43230392&guid=72cd4c68-29bc-44da-a79b-3fbe2fb90f33&scheme=1)[<https://www2.cloud.editorialmanager.com/pone/download.aspx?id=43230392&guid=72cd4c68-29bc-44da-a79b-3fbe2fb90f33&scheme=1>](https://www2.cloud.editorialmanager.com/pone/download.aspx?id=43230392&guid=72cd4c68-29bc-44da-a79b-3fbe2fb90f33&scheme=1)

Figure	[Click here to access/download;Figure;Figure 3.png](https://www2.cloud.editorialmanager.com/pone/download.aspx?id=43230393&guid=12171172-99af-42b3-9b74-7fcaccc89bf8&scheme=1)[<https://www2.cloud.editorialmanager.com/pone/download.aspx?id=43230393&guid=12171172-99af-42b3-9b74-7fcaccc89bf8&scheme=1>](https://www2.cloud.editorialmanager.com/pone/download.aspx?id=43230393&guid=12171172-99af-42b3-9b74-7fcaccc89bf8&scheme=1)
