# Supplementary material for: Clinical course and outcomes of corneal blood staining in open-globe injury: Eye Injury Vitrectomy Study
Source: PLoS One. 2026 Aug 4;21(8):e0354967. doi: 10.1371/journal.pone.0354967 (PMC13436741; doi:10.1371/journal.pone.0354967)
Supplement: Supplementary file 7 [file pone.0354967.s005.docx]

**PLOS One**

**Clinical course and outcomes of corneal blood staining in open-globe injury: Eye Injury Vitrectomy Study**

--Manuscript Draft--

| **Manuscript Number:** | PONE-D-26-00677R1 |
| --- | --- |
| **Article Type:** | Research Article |
| **Full Title:** | Clinical course and outcomes of corneal blood staining in open-globe injury: Eye Injury Vitrectomy Study |
| **Short Title:** | Corneal blood staining in open-globe injury: EIVS |
| **Corresponding Author:** | Zhi-zhong Ma, M.D.  Peking University Third Hospital Beijing, CHINA |
| **Keywords:** | open-globe injury; corneal blood staining; temporary keratoprosthesis; penetrating keratoplasty; vitrectomy |
| **Abstract:** | Purpose: To describe the clinical course and outcomes of corneal blood staining in open-globe injuries (OGIs) after vitrectomy without routine immediate allograft penetrating keratoplasty (PKP).  Methods: This study included 42 eyes of 42 patients with corneal blood staining secondary to OGIs. Clinical data were retrospectively collected from the Eye Injury Vitrectomy Study (EIVS). All eyes underwent anterior chamber washout at the start of pars plana vitrectomy (PPV). When visualization remained inadequate, temporary keratoprosthesis (TKP)-assisted PPV was performed, followed by replacement of the trephined autologous cornea (TKP + PPV + RTC). Postoperative outcomes, including corneal transparency, best-corrected visual acuity (BCVA), intraocular pressure (IOP), and need for subsequent PKP, were assessed.  Results: Median age was 36.5 years; globe rupture accounted for 95.2%. Preoperative hypotony (IOP ≤10 mmHg) was present in 90.5%, with severe posterior segment injury in all eyes. Eight eyes achieved sufficient corneal clarity after washout alone; the remaining 34 required TKP + PPV + RTC. During follow-up, varying degrees of spontaneous clearance occurred in most eyes, forming a peripheral “window” that enabled fundus assessment. At the final follow-up, BCVA improved from median 4.00 logMAR preoperatively to 2.80 logMAR (p = 0.002), and median IOP rose from 5.00 to  10.00 mmHg (p = 0.002). Five eyes developed phthisis bulbi, four eyes achieved complete silicone oil removal, and only 1 eye underwent allograft PKP.  Conclusions: The strategy of anterior chamber washout first, followed by TKP + PPV + RTC when necessary, provides timely posterior segment access while preserving the globe and conserving corneal tissue in severe OGIs complicated by corneal blood staining. These findings support selective, rather than routine, PKP during follow-up. |
| **Order of Authors:** | Bingjie Wang, M.D. |
|  | Kang Feng, M.D. |
|  | Yao Lu, M.D. |
|  | Huijin Chen, M.D. |
|  | Liang Han, M.D. |
|  | Aihua Ding |
|  | Haikun Wang |
|  | Shaofeng Gu |
|  | Zhi-zhong Ma, M.D. |
| **Response to Reviewers:** | Reviewer #1: This article is generally well written and only requires minor revision. Some suggestions  Line 67 severe could be replaced by "the worst visual outcomes' Line 113 , define what is an experience physician |

|  | Thank you very much for the positive evaluation of our manuscript and for these helpful suggestions.  For Line 67, we have revised the sentence accordingly. (Line 49, Page 4) “Open-globe injuries (OGIs) are ocular injuries associated with the worst visual  outcomes and may result in monocular or bilateral blindness, particularly in eyes with globe rupture ”  For the definition of “experience physician”. We agree that the term “experienced physician” was not sufficiently specific and could be misleading. We intended to indicate trained clinicians who were able to align serial AS-OCT scans by using anatomical landmarks and previous scan locations as references. We have revised the Methods to clarify this point. (Line 96- 99, Page 6)  “…AS-OCT (Wavelength nanometer, Tomey CASIA2, Nagoya, Japan) was performed by trained clinicians (AD, HW, and SG), with serial scans were obtained from same corneal regions whenever possible, using anatomical landmarks and previous scan locations as references.”  Please find the revisions highlighted in red.  Reviewer #2: Reviewer Comments  This manuscript addresses an uncommon but clinically relevant problem: the management of corneal blood staining in severe open-globe injury requiring posterior segment surgery. The authors present a conservative strategy in which anterior chamber washout is performed first, followed by TKP-assisted PPV with replacement of the trephined autologous cornea when visualization remains inadequate, while avoiding routine immediate allograft PKP. The topic is important, the case series is valuable for a relatively rare clinical scenario, and the photographic documentation is a strength. However, the manuscript requires major revision before it can be considered for publication. The main issues relate to incomplete characterization of cohort selection and follow-up, lack of a clearly defined corneal clearance outcome, limited analysis, underdeveloped AS-OCT reporting, and conclusions that are somewhat stronger than the data currently support.  Thank you for your comments.  Major comments   1. Study design and cohort selection need clearer reporting.   The manuscript states that this is a retrospective analysis of EIVS data, but it is unclear whether the 42 eyes represent a consecutive case series, a selected registry subset, or all eligible cases during a defined period (which was not stated). The authors should state the period during which the cases were selected, whether patients were consecutive, the number screened or excluded, and reasons for exclusion. A simple flow diagram would improve transparency and help readers assess selection bias and generalizability.  Thanks for this valuable suggestion.  This was a retrospective analysis of cases retrieved from the EIVS database between June 2012 and June 2025. As defined by the EIVS database, the general inclusion criteria were patients who suffered from severe eye injury and were treated with vitreoretinal surgery, enucleation, or evisceration [1].  A total of 3,152 eyes in the EIVS database were screened, including 1,920 eyes with open-globe injuries. Among them, 157 eyes had documented corneal blood staining. After excluding 115 eyes because of endophthalmitis, pre-existing ocular diseases, incomplete clinical or photographic records, follow-up shorter than 12 months, or enucleation/evisceration within 12 months; 42 eyes were included in the final analysis. We have added these details to the Methods and Results, and provided a flow diagram as S1 Figure.  Please find them highlighted in red (Line 81-83, Page 5; Line 89- 90, Page 6; Line 124-  128, Page 7)   2. The central corneal outcome is not operationally defined. |
| --- | --- |

|  | The objective of this paper was to describe clinical course and outcome of corneal blood staining following the interventions. Yet, the paper’s main conclusion rests on “spontaneous clearance,” “corneal clarity,” and formation of a peripheral “window,” yet these outcomes are described narratively rather than systematically. The authors should define what constitutes sufficient clarity for PPV after washout, and how corneal status was graded clinically, photographically, or by AS-OCT, and whether grading was performed by one or more observers. A formal grading system or at least reproducible categories would make the conclusions more credible.  We appreciate this comment.  In the revised manuscript, we defined “sufficient clarity for PPV after anterior chamber washout” as intraoperative corneal clarity that allowed the surgeon to safely identify major posterior segment structures and proceed with PPV without the use of TKP. We acknowledge that this criterion inevitably contains some degree of clinical judgment, because the feasibility of PPV depends not only on the size and location of the clear corneal area, but also on the extent of posterior segment pathology, the presence of iris or blood clot obstruction, and the surgeon’s experience. In this study, PPV without TKP was attempted only when the operating surgeon (including KF, YL, HC, LH and ZZM) considered that a continuous, unobstructed corneal viewing area was sufficient to permit safe posterior segment visualization and surgical manipulation. Otherwise, TKP-assisted PPV was performed. (Line 107- 113, Page 6 and 7, highlighted in red) We also added reproducible postoperative corneal outcome categories, including complete clearing, partial clearing, and persistent opacity. “Complete clearing” was defined as complete disappearance of opacity attributable to corneal blood staining during follow-up, although other non-blood-staining corneal changes, such as scarring or band keratopathy, could still be present. “Partial clearing” was defined as recognizable decrease of corneal blood staining compared with the preoperative status, with formation of a clear corneal area sufficient for clinical posterior segment assessment, including retinal status and intraocular tamponade status. “Persistent opacity” was defined as residual corneal opacity due to blood staining that showed little or no regression compared with the preoperative status and continued to limit corneal transparency or fundus visualization.  Serial slit-lamp photographs, fundus photographs when available, and clinical records were independently reviewed by two ophthalmologists (KF and LH) to classify the corneal outcomes. Disagreements were resolved by a senior ophthalmologist (ZZM). These definitions have been added to the Methods (Line 114- 116, Page 7), and the corresponding subgroup results have been summarized in the Results (Line 140- 143, Page 8) and Table 1.  3. Follow-up duration must be reported more adequately.  Since corneal blood staining clearance is inherently time-dependent, the actual follow-up duration of the cohort is essential. The manuscript should provide the median follow-up with IQR or range, and ideally also report time to partial clearance, time to complete clearance where achieved, and time to eventual PKP in the one transplanted eye.  Thank you for highlighting this issue. We agree that follow-up duration is essential for interpreting the natural course of corneal blood staining. Previous reports have shown substantial variability in the speed of spontaneous clearing. In traumatic hyphema-related corneal blood staining, partial clearing has been observed within weeks to months, whereas complete clearing may require 10- 15 months or even up to 2- 3 years in some cases [2-5]. This variability suggests that the course of clearance may be influenced by the severity of corneal blood staining, as well as the degree of damage to the corneal endothelium and stroma.  In our series, all eyes had trauma-related corneal blood staining and underwent anterior chamber washout during surgery; however, the degree of hyphema, preoperative IOP, interval from injury to surgery, and tamponade / anterior segment status varied among patients. These factors may have contributed to heterogeneity in the timing and extent of corneal clearing.  Accordingly, we have added the median follow-up duration of 36 months (IQR, 23- 42 months; range, 12- 60 months) to the Results (Line 135- 136, Page 8) and Table 1. We also reviewed the available clinical records and serial photographs and reported the first documented visit at which partial or complete clearing was observed (S1 Table).  The timing for corneal clearing varies widely among individual patients, as it depends |
| --- | --- |

|  | on the severity and extent of the pre-operative opacity , as well as the severity of the ocular trauma. Specifically, the median time to partial clearing was 3 months (IQR, 3-7.5 months), and the median time to complete clearing was 24 months (IQR, 10- 30 months). Given the retrospective nature of this cohort, follow-up visits were not scheduled at uniform intervals; thus the exact onset of corneal clearing could not be precisely determined for every eye, and only the timing of actual follow-up visits could be verified. We have clarified this limitation in the revised manuscript. (Page 15 and 16)  For the single eye that underwent allograft PKP, we now state that PKP was performed 14 months after the initial surgery, together with the ocular status at that time (Line  173- 183, Page 9 and 10).   4. The results should more clearly describe what happened to each subgroup.   The manuscript reports that 8 eyes achieved adequate clarity after anterior chamber washout alone, while 34 required TKP + PPV + RTC, but the later corneal course of each subgroup is not fully summarized. The authors should state:  •how many eyes achieved complete versus partial clearing  •how many retained persistent central opacity or scarring  •how many developed band keratopathy  •whether all eyes eventually achieved a clinically useful fundus view,  •and whether corneal outcomes differed between the washout-only and TKP groups. This has a tendency to further strengthen the clinical message.  Thank you for this suggestion. In the supplementary materials, we have added a subgroup analysis comparing the washout-only group and the TKP + PPV + RTC group. The numbers of eyes with complete clearing, partial clearing, and persistent opacity have been added to the Results (Line 140- 144, Page 8) and Table 1.  Persistent central opacity or scarring was observed in 17/42 eyes. In this cohort of severe OGIs, residual central opacity may have resulted from multiple factors in addition to corneal blood staining, including the original corneal wound, suture-related scarring, and band keratopathy. Final visual function was also strongly affected by lens loss and severe posterior segment injuries, such as retinal detachment, choroidal detachment, and retinal tissue loss. Therefore, the independent functional impact of central opacity could not be reliably isolated in this retrospective study.  Band keratopathy was observed in 9 of 42 eyes, all of which were tamponaded with silicone oil. Because band keratopathy has been associated with long-term silicone oil tamponade, aphakia (which may facilitate direct contact between silicone oil and the corneal endothelium), hypotony, repeated intraocular surgery, and chronic inflammation [6, 7], we did not interpret it as a direct consequence of corneal blood staining. Instead, it is reported as a postoperative corneal complication in the context of severe ocular trauma.  We further clarified whether a clinically useful fundus view was achieved in each subgroup, including eyes with complete and partial clearing. In anterior chamber washout group (n = 8), 4/8 achieved complete clearing, while 4/8 showed partial clearing. In the TKP + PPV + RTC group (n = 34), 9/34 exhibited complete clearing, 18/34 presented partial clearing, and 7/34 suffered from persistent opacity.  For eyes with corneal opacity that limited direct visualization, including during the early postoperative period (e.g., at 1 week), posterior segment assessment was performed using B-scan ultrasonography, visual function examinations, and IOP measurement. The decision to perform subsequent PKP is strictly based on a comprehensive evaluation of both visual function and the anatomical viability of the injured eye; otherwise, allogeneic PKP is not recommended. (Line 209- 213, Page 12)  The data suggested that eyes in the washout-only group generally demonstrated faster and more complete clearing of corneal blood staining (please find it in S1 Table). Given the limited sample size and baseline imbalance, these subgroup findings are presented descriptively.   5. AS-OCT is underdeveloped and should either be analyzed or toned down.   AS-OCT is included in the methods and highlighted in a representative figure, yet no cohort-level imaging findings are presented. If AS-OCT was routinely performed, the authors should summarize findings such as stromal hyperreflectivity, corneal thickness, and correlation with final corneal status. If it was not analyzable in a systematic way, then the discussion of AS-OCT’s prognostic value should be more cautious. |
| --- | --- |

|  | We agree with the reviewer. In the original manuscript, AS-OCT was overemphasized relative to the available cohort-level imaging data. Although AS-OCT was useful for documenting representative anatomical changes, imaging was not performed using a standardized quantitative protocol at fixed postoperative intervals in all eyes.  Therefore, reliable cohort-level analysis of stromal hyperreflectivity, corneal thickness, or correlations with final corneal status was not feasible. In the revised manuscript, we have toned down the AS-OCT-related statements, retained AS-OCT only as representative images, and added the lack of standardized serial AS-OCT analysis as a limitation. (Page 15 and 16)   6. Visual outcome interpretation should be more cautious.   The manuscript reports statistically significant improvement in BCVA, but the cohort remains profoundly visually compromised overall, with many eyes presenting with NLP, many remaining NLP, and a proportion progressing to phthisis. The discussion and conclusion should avoid implying a strong functional benefit and should instead emphasize that this may be a globe-preserving and cornea-conserving strategy in eyes with otherwise poor prognosis.  We appreciate this comment. We agree that although BCVA showed statistical improvement, the cohort remained severely visually compromised overall, and the visual outcome was mainly limited by the severity of posterior segment injury. We have deleted this sentence in Result and revised the Discussion and Conclusion to avoid implying a strong functional benefit. The revised manuscript now emphasizes that this approach should be interpreted primarily as a globe-preserving and cornea-conserving strategy that may reduce exposure to prolonged postoperative corticosteroid or immunosuppressive therapy and lower the risk of graft rejection in eyes with limited visual potential. (Page 13, 15, 16)   7. Silicone oil status and IOP outcomes need deeper contextualization.   The paper notes that many eyes required additional silicone oil procedures and that only a few achieved complete oil removal. This is highly relevant because final IOP and eventual candidacy for corneal transplantation are likely influenced by recurrent posterior pathology, persistent hypotony, and the long-term graft-host environment.  The manuscript should clarify how many eyes had secondary procedures, how many had oil refill, partial removal, or re-tamponade, whether final IOP was measured under oil in most eyes, and whether phthisical eyes were included in the IOP analysis.  Thank you for this suggestion. We agree that silicone oil status, IOP, and secondary procedures are important for interpreting final ocular status and later PKP candidacy. In this cohort, all eyes were followed for at least 12 months. Among the 40 eyes initially tamponaded with silicone oil, only 4 achieved complete silicone oil removal by the final visit. Therefore, final IOP in most eyes was measured in the presence of silicone oil or partial silicone oil tamponade. The remaining eyes required one or more silicone oil-related secondary procedures during follow-up, including oil refill, partial oil removal, or oil removal with re-tamponade, mainly because of silicone oil emulsification, persistent hypotony, or recurrent posterior segment instability. Some eyes underwent more than one such procedure.  Silicone oil emulsification is a well-recognized complication of long-term tamponade, with previous studies reporting an increased risk when silicone oil remains in the eye for more than 6 months [8], an average onset of approximately 13 months [9], and very high rates after 12 months of tamponade [10]. Thus, in this cohort with follow-up of at least 12 months, repeated silicone oil-related procedures were not unexpected.  We also clarified that phthisis bulbi (n=5) were included in the overall anatomical outcome analysis. These findings further support our view that corneal transplantation candidacy in this cohort were strongly influenced by posterior segment status, tamponade condition, and overall ocular viability, rather than by corneal blood staining alone.  These findings suggest that many eyes remained in an unstable ocular condition, with persistent hypotony, long-term tamponade, or repeated posterior segment procedures, which may be unfavorable for graft survival after PKP.   8. The indication and context for the single allograft PKP should be stated explicitly. Because only one eye underwent PKP, that case is clinically informative. The manuscript should state the indication, timing, tamponade status, ocular condition, and visual/anatomical rationale for proceeding with PKP. This would help define the |
| --- | --- |

|  | authors’ threshold for delayed transplantation.  Thank you for this suggestion. We have revised the Results to provide additional details of the single case that underwent delayed allograft PKP. (Line 173- 183, Page 9 and 10)  “Only one eye underwent delayed allograft PKP. This was a gas-tamponaded eye in a 46-year-old man with traumatic aniridia, aphakia after prior lens extraction in local hospital, vitreous hemorrhage, and post-traumatic secondary glaucoma. Although preoperative B-scan suggested choroidal detachment, this finding were not confirmed intraoperatively, and laser photocoagulation was applied around the retinal break after retinal attachment. The RTC gradually clearing, and BCVA reached 0.5 at 14 months after the initial surgery, with well-controlled IOP, indicating favorable visual potential and a relatively stable posterior segment. Allograft PKP combined with scleral-fixated IOL implantation was therefore performed for optical rehabilitation and aphakia correction. Early post-PKP follow-up was available, but the patient was subsequently lost to follow-up. When he returned 3 years after PKP, the eye had progressed to NLP due to glaucoma, with epithelial edema but relatively preserved stromal clarity in corneal graft.”   9. The manuscript would benefit from association or predictor analyses.   The current analysis is mainly descriptive. Even in a modest sample, simple univariable or subgroup analyses would be useful. Potential comparisons include: washout-only versus TKP + PPV + RTC, complete versus incomplete corneal clearing, phthisis versus non-phthisis, final NLP versus retained light perception, eyes with and without useful fundus assessment, and associations with age, injury type (especially among the majority who had globe rupture), hypotony, retinal detachment, choroidal detachment, tamponade type, or no retina detected. This would strengthen the manuscript beyond a purely descriptive case series.  We appreciate this thoughtful suggestion. Given the small sample size and the highly imbalanced distribution of injury severity, including choroidal detachment, retinal detachment, retinal tissue loss, hypotony, and tamponade type, multivariable prediction modeling would be statistically underpowered and could lead to potentially misleading conclusions.  In this cohort of severe open-globe injuries, final visual and anatomical outcomes were largely determined by posterior segment damage and overall ocular viability.  Therefore, the independent contribution of corneal blood staining to global outcomes, such as final BCVA, phthisis bulbi, or complete silicone oil removal, could not be reliably isolated in this retrospective dataset.  To provide additional clinical information while avoiding overinterpretation, we added an exploratory descriptive comparison between the washout-only group and the TKP + PPV + RTC group in S1 Table and S2 Table. The parameters included final corneal status, final BCVA, final IOP, and complete silicone oil removal. Eyes in the washout-only group tended to show faster and more complete clearance of corneal blood staining; however, this subgroup included only 8 eyes and likely represented eyes with less severe initial corneal opacity. Therefore, these findings are presented descriptively rather than as formal comparative or predictive analyses.   10. The strategy should be positioned more clearly relative to alternative approaches. The discussion would be improved by explicitly comparing this approach with TKP plus immediate PKP, endoscopic vitrectomy, staged surgery, and observation before PPV where feasible. This would help readers understand when the proposed conservative approach is most appropriate in real-world practice.   Thank you for this comment. We respectfully emphasize that, in severe open-globe injuries with posterior segment pathology requiring PPV, observation for spontaneous corneal clearing should not be regarded as a clinically appropriate alternative.  Corneal blood staining may clear spontaneously, but this process can take months to years. In contrast, the timing of PPV after severe ocular trauma is critical. Delaying PPV increase the risk of proliferative vitreoretinopathy, which lead to irreversible anatomical or functional loss [11-13]. Therefore, the clinical question in these eyes is not whether to wait for corneal clearing before PPV, but how to obtain adequate posterior segment access while avoiding unnecessary high-risk allograft PKP in the acute traumatic setting. |
| --- | --- |

|  | Our strategy addresses this problem by performing anterior chamber washout first, followed by TKP-assisted PPV with replacement of the trephined autologous cornea when visualization remains inadequate. Compared with TKP followed by immediate allograft PKP, this approach avoids placing donor tissue into an acutely inflamed and often silicone-oil-filled eye. Although endoscopic vitrectomy can bypass corneal opacity, it is not yet a widely adopted standard approach in many centers and remains limited by a narrower field of view, lower image resolution, specialized equipment requirements, and a steep learning curve. These were discussed in the Introduction of original manuscript and we have revised them in Discussion to clarify this clinical positioning further. (Page 15)  Minor comments   1. Ethics approval inconsistency.   The ethics number differs between the submission material and the manuscript body and should be corrected.  Thank you for pointing this out. We apologize for the typo in the submission materials. The ethics approval number has been corrected.   2. Grammar and wording issues.   Several language issues need correction, including awkward or ungrammatical phrases.  Thank you for pointing this out. These have been corrected.   3. Table 1.   Table 1 would be more informative if it included follow-up duration, number of reoperations, timing of silicone oil removal/refill, corneal outcome categories, and final transparency status.  Thank you for pointing this out. We have expanded Table 1.   4. Discussion wording and internal consistency.   Some phrases in the discussion are awkward and should be revised for clarity. There also appears to be an incorrect figure reference when discussing the case with complete clearance (check inserted comments).  Thank you for pointing this out. We have revised them accordingly. Please find them highlighted in red.   5. References.   The reference list should be reviewed carefully for journal formatting and typographic consistency.  Sorry for the formatting and typographic inconsistency in the reference list. The references were now formatted using the 'Vancouver' style via EndNote, as recommended in the journal's submission guidelines. We also have manually checked and corrected the reference formatting for consistency and accuracy. Please let us know if any further changes are necessary. Thank you for pointing this out.  Overall recommendation  This is a clinically relevant manuscript with useful photographic documentation and an important practical message, especially in eyes with poor prognosis and limited donor-tissue justification. However, the paper currently needs substantial revision in outcome definition, follow-up reporting, and discussion balance before it is ready for publication.   1. Feng, K., Y.T. Hu, and Z. Ma, Prognostic indicators for no light perception after open-globe injury: eye injury vitrectomy study. Am J Ophthalmol, 2011. 152(4): p. 654-662.e2. 2. Brodrick, J.D., Corneal blood staining after hyphaema. Br J Ophthalmol, 1972. 56(8):   p. 589-93.   3. Yang, X., Q. Zhou, and S. Du, Conjunctival flap covering in the treatment of corneal blood staining. Can J Ophthalmol, 2011. 46(5): p. 442-3. 4. Lin, H.Y. and K.L. Peng, Gradual spontaneous resolution of corneal bloodstaining |
| --- | --- |

|  | after anterior chamber irrigation for rebleeding secondary to traumatic hyphema. Int Med Case Rep J, 2018. 11: p. 239-242.   5. Hwang, B.E., et al., Serial Photographic Monitoring of Spontaneous Clearance of Corneal Blood Stain in a Child with Traumatic Hyphema. Korean J Ophthalmol, 2017. 31(3): p. 281-282. 6. He, K., et al., Risk Factors for Band Keratopathy in Aphakic Eyes With Silicone Oil Tamponade for Open-Globe Injuries: A Multicenter Case-Control Study. Front Med (Lausanne), 2021. 8: p. 713599. 7. Bennett, S.R. and G.W. Abrams, Band keratopathy from emulsified silicone oil. Arch Ophthalmol, 1990. 108(10): p. 1387. 8. Ni, Y., et al., Analysis of the causative factors related to earlier emulsification of silicone oil. Int J Ophthalmol, 2019. 12(3): p. 517-519. 9. Toklu, Y., et al., Time course of silicone oil emulsification. Retina, 2012. 32(10): p. 2039-44. 10. Federman, J.L. and H.D. Schubert, Complications associated with the use of silicone oil in 150 eyes after retina-vitreous surgery. Ophthalmology, 1988. 95(7): p. 870-6. 11. Ozdek, S., M. Hasanreisoglu, and E. Yuksel, Chorioretinectomy for perforating eye injuries. Eye, 2013. 27(6): p. 722-727. 12. Chauhan, M.Z., et al., Outcomes of pars plana vitrectomy following ocular trauma at varying surgical time points. International Journal of Retina and Vitreous, 2022. 8(1): p. 49. 13. Miller, S.C., et al., Global Current Practice Patterns for the Management of Open Globe Injuries. American Journal of Ophthalmology, 2022. 234: p. 259-273. |
| --- | --- |
| **Additional Information:** |  |
| **Question** | **Response** |
| **Financial Disclosure**  Enter a financial disclosure statement that describes the sources of funding for the work included in this submission. Review the [submission guidelines](http://journals.plos.org/plosone/s/submission-guidelines#loc-financial-disclosure-statement) for detailed requirements. View published research articles from [*PLOS ONE*](https://journals.plos.org/plosone/) for specific examples.  This statement is required for submission and **will appear in the published article** if the submission is accepted. Please make sure it is accurate. | The author(s) received no specific funding for this work. |
| **Funded studies**  Enter a statement with the following details:   - Initials of the authors who received each award - Grant numbers awarded to each author - The full name of each funder - URL of each funder website - Did the sponsors or funders play any role in the study design, data collection and analysis, decision to publish, or preparation of the manuscript? |  |
|  |  |

| Did you receive funding for this work? |  |
| --- | --- |
| **Competing Interests**  Use the instructions below to enter a competing interest statement for this submission. On behalf of all authors, disclose any [competing interests](http://journals.plos.org/plosone/s/competing-interests) that could be perceived to bias this work—acknowledging all financial support and any other relevant financial or non-financial competing interests.  This statement is required for submission and **will appear in the published article** if the submission is accepted. Please make sure it is accurate and that any funding sources listed in your Funding Information later in the submission form are also declared in your Financial Disclosure statement.  View published research articles from  [*PLOS ONE*](http://journals.plos.org/plosone/) for specific examples. | The authors have declared that no competing interests exist. |
| **NO authors have competing interests**  Enter: *The authors have declared that no competing interests exist.*  **Authors with competing interests**  Enter competing interest details beginning with this statement:  *I have read the journal's policy and the authors of this manuscript have the following competing interests: [insert competing interests here]* |  |
| * typeset |  |
| **Data Availability**  Authors are required to make all data underlying the findings described fully available, without restriction, and from the time of publication. PLOS allows rare exceptions to address legal and ethical concerns. See the [PLOS Data Policy](http://journals.plos.org/plosone/s/data-availability) and | No - some restrictions will apply |

| [FAQ](http://journals.plos.org/plosone/s/data-availability#loc-faqs-for-data-policy) for detailed information.  A Data Availability Statement describing where the data can be found is required at submission. Your answers to this question constitute the Data Availability Statement and **will be published in the article**, if accepted. |  |
| --- | --- |
| **Important:** Stating ‘data available on request from the author’ is not sufficient. If your data are only available upon request, select ‘No’ for the first question and explain your exceptional situation in the text box. |  |
| Do the authors confirm that all data underlying the findings described in their manuscript are fully available without restriction? |  |
| Additional data availability information: |  |
| **Ethics Statement**  Enter an ethics statement for this submission. This statement is required if the study involved:   - Human participants - Human specimens or tissue - Vertebrate animals or cephalopods - Vertebrate embryos or tissues - Field research   Write "N/A" if the submission does not require an ethics statement.  General guidance is provided below. Consult the [submission guidelines](http://journals.plos.org/plosone/s/submission-guidelines#loc-guidelines-for-specific-study-types) for detailed instructions. **Make sure that all information entered here is included in the Methods section of the manuscript.** | The study was approved by the Institutional Review Board of Peking University Third Hospital Medical Science Research Ethics Committee (Ethic No. IRB00006761-2012060) and conducted in accordance with the Declaration of Helsinki. Clinical data were retrospectively collected from the Eye Injury Vitrectomy Study (EIVS). The trial was registered with the Chinese Clinical Trial Registry (ChiCTR; Number: ChiCTR-OCH-08000199) on November 28, 2008. |

| **Format for specific study types**  **Human Subject Research (involving human participants and/or tissue)**   - Give the name of the institutional review board or ethics committee that approved the study - Include the approval number and/or a statement indicating approval of this research - Indicate the form of consent obtained (written/oral) or the reason that consent was not obtained (e.g. the data were analyzed anonymously)   **Animal Research (involving vertebrate animals, embryos or tissues)**   - Provide the name of the Institutional Animal Care and Use Committee (IACUC) or other relevant ethics board that reviewed the study protocol, and indicate whether they approved this research or granted a formal waiver of ethical approval - Include an approval number if one was obtained - If the study involved *non-human primates*, add *additional details* about animal welfare and steps taken to ameliorate suffering - If anesthesia, euthanasia, or any kind of animal sacrifice is part of the study, include briefly which substances and/or methods were applied   **Field Research**  Include the following details if this study involves the collection of plant, animal, or other materials from a natural setting:   - Field permit number - Name of the institution or relevant body that granted permission |  |
| --- | --- |
|  |  |
| **Data Availability**  Before publication, Authors are required to make fully available and without restriction all data underlying their findings. Sharing the data underlying your findings increases the visibility, credibility, and impact of your work, while supporting transparency and future scientific discovery. Please see our [PLOS Data](https://journals.plos.org/plosone/s/data-availability) | Data cannot be shared publicly because they involve clinical information and are subject to restrictions imposed by the Institutional Review Board of Peking University Third Hospital Medical Science Research Ethics Committee . Data are available from the Ethics Committee (contact via [wangsiya745@126.com](mailto:wangsiya745@126.com)) for researchers who meet the criteria for access to confidential data. |

| [Policy page](https://journals.plos.org/plosone/s/data-availability) for detailed information on this policy.  A **Data Availability Statement**, detailing where the data can be accessed, is required at first submission. Insert your Data Availability Statement in the box below.  Please see the [data reporting](https://journals.plos.org/plosone/s/submission-guidelines#loc-data-reporting) section of our submission guidelines for instructions on what you need to include in your Data Availability Statement. Examples of acceptable Data Availability Statements are included here for reference:   1. The minimal data set [and accompanying code, where generated] is available at [repository name] via [DOI hyperlink] 2. No data was generated by this study. The following existing data sources were used: [dataset name] from [dataset location] available via [web address or DOI hyperlink]. 3. For Study Protocols: No datasets were generated or analysed during the current study. All relevant data from this study will be made available upon study completion.   *PLOS allows rare exemptions to address legal and ethical concerns. If you have legal or ethical restrictions, please use the box below to detail these in full sentences for the Journal team to consider.*  This statement will be typeset if the manuscript is accepted for publication. |  |
| --- | --- |

<https://www2.cloud.editorialmanager.com/pone/download.aspx?id=44823304&guid=ca75a736-7b4e-475b-a384-a454a22129a9&scheme=1>Manuscript	[Click here to](https://www2.cloud.editorialmanager.com/pone/download.aspx?id=44823304&guid=ca75a736-7b4e-475b-a384-a454a22129a9&scheme=1)

[access/download;Manuscript;CBS_revision_v2_clean2.docx](https://www2.cloud.editorialmanager.com/pone/download.aspx?id=44823304&guid=ca75a736-7b4e-475b-a384-a454a22129a9&scheme=1)

1 **Clinical course and outcomes of corneal blood staining in open-globe injury: Eye Injury**
2 **Vitrectomy Study**
3 Bingjie Wang^1,2^, Kang Feng ^1^, Yao Lu ^1^, Huijin Chen^1^, Liang Han^1^, Aihua Ding^1^, Haikun Wang^1^,
4 Shaofeng Gu^1^, Zhizhong Ma ^1*^
5 ^1^Department of Ophthalmology, Peking University Third Hospital, Beijing, China
6 ^2^Eye Center, Beijing Tsinghua Changgung Hospital, School of Clinical Medicine, Tsinghua
7 University, Beijing, China

8

9

##### 10 Key message:

11 What is known
12 1. Corneal blood staining in open-globe injuries (OGIs) traditionally requires immediate allograft
13 penetrating keratoplasty (PKP) to enable posterior segment access.
14 2. Immediate PKP in the acute phase is high-risk due to intense inflammation, hypotony, and long-term
15 silicone oil tamponade.
16 What is new
17 1. This study highlights a globe-preserving and cornea-conserving strategy for severe OGIs complicated
18 by corneal blood staining, using anterior chamber washout first, and TKP-assisted PPV with replacement
19 of the trephined autologous cornea when necessary.
20 2. By avoiding routine immediate allograft PKP in the acute traumatic setting, this approach preserves
21 the opportunity for later keratoplasty to be selected after ocular stabilization, based on overall ocular
22 status, corneal condition, and visual potential, while reducing graft-related risks.

23

24	**Abstract**

25 Purpose: To describe the clinical course and outcomes of corneal blood staining in open-globe injuries
26 (OGIs) after vitrectomy without routine immediate allograft penetrating keratoplasty (PKP).
27 Methods: This study included 42 eyes of 42 patients with corneal blood staining secondary to OGIs.
28 Clinical data were retrospectively collected from the Eye Injury Vitrectomy Study (EIVS). All eyes
29 underwent anterior chamber washout at the start of pars plana vitrectomy (PPV). When visualization
30 remained inadequate, temporary keratoprosthesis (TKP)-assisted PPV was performed, followed by
31 replacement of the trephined autologous cornea (TKP + PPV + RTC). Postoperative outcomes, including
32 corneal transparency, best-corrected visual acuity (BCVA), intraocular pressure (IOP), and need for
33 subsequent PKP, were assessed.
34 Results: Median age was 36.5 years; globe rupture accounted for 95.2%. Preoperative hypotony (IOP
35 ≤10 mmHg) was present in 90.5%, with severe posterior segment injury in all eyes. Eight eyes achieved
36 sufficient corneal clarity after washout alone; the remaining 34 required TKP + PPV + RTC. During
37 follow-up, varying degrees of spontaneous clearance occurred in most eyes, forming a peripheral
38 “window” that enabled fundus assessment. At the final follow-up, BCVA improved from median 4.00
39 logMAR preoperatively to 2.80 logMAR (p = 0.002), and median IOP rose from 5.00 to 10.00 mmHg (p

40	= 0.002). Five eyes developed phthisis bulbi, four eyes achieved complete silicone oil removal, and only

41 1 eye underwent allograft PKP.
42 Conclusions: The strategy of anterior chamber washout first, followed by TKP + PPV + RTC when
43 necessary, provides timely posterior segment access while preserving the globe and conserving corneal
44 tissue in severe OGIs complicated by corneal blood staining. These findings support selective, rather
45 than routine, PKP during follow-up.
46 **Keywords:** open-globe injury, corneal blood staining, temporary keratoprosthesis, penetrating
47 keratoplasty, vitrectomy

# 48 Introduction

49 Open-globe injuries (OGIs) are ocular injuries associated with the worst visual outcomes and may result
50 in monocular or bilateral blindness, particularly in eyes with globe rupture [1, 2]. Adequate corneal
51 transparency is essential for visualization of posterior segment pathology. In severe ocular trauma,
52 however, corneal transparency may be compromised, limiting clinical assessment and impeding surgical
53 repair.
54 Among trauma-related corneal opacities, corneal blood staining is one of the most challenging conditions.
55 It is characterized by deposition of hemoglobin and its breakdown products within the corneal stroma
56 [3], most commonly following prolonged hyphema and elevation of intraocular pressure (IOP) [4, 5],
57 but it has also been reported in eyes with low IOP [6, 7]. Disruption of endothelial function, stromal
58 impregnation by erythrocyte derivatives, and impaired metabolic clearance contribute to its development
59 [8].
60 In eyes with corneal blood staining requiring pars plana vitrectomy (PPV), a temporary keratoprosthesis
61 (TKP) is commonly used to facilitate posterior segment visualization [9]. This approach, however,
62 necessitates donor tissue for subsequent allograft penetrating keratoplasty (PKP) and is associated with
63 risks of graft rejection and infection related to long-term immunosuppression [10]. Furthermore, given
64 the poor prognosis in eyes with extensive posterior segment damage, the indication for immediate PKP
65 may be uncertain. Although endoscopy-assisted vitrectomy has been proposed as an alternative, its
66 adoption is limited by low image resolution, restricted field of view, specialized equipment requirements,
67 and a steep learning curve [11-13].
68 In this study, we evaluated a modified corneal conservative surgical strategy for OGIs complicated by
69 corneal blood staining, and analyzed anatomical and functional outcomes, as well as the evolution of
70 corneal blood staining.

71	**Methods**

## 72 Human ethics and consent to participate declarations

73 This study was approved by the Institutional Review Board of Peking University Third Hospital Medical
74 Science Research Ethics Committee (Ethics No. IRB00006761-2012060) and was conducted in
75 accordance with the Declaration of Helsinki. Clinical data were collected from the Eye Injury Vitrectomy
76 Study (EIVS). The trial was registered with the Chinese Clinical Trial Registry (ChiCTR; Number:
77 ChiCTR-OCH-08000199) on November 28, 2008. Written informed consent was obtained from all
78 participants at the time of enrollment in the EIVS, including consent for the use of non-personally
79 identifiable medical records for future research. For participants younger than 18 years of age, written
80 informed consent was obtained from their parents or legal guardians. The present study represents a
81 retrospective analysis of EIVS data from Peking University Third Hospital between June 2012 and June
82 2025. As defined by the EIVS database, the general inclusion criteria were patients who suffered from
83 severe eye injury and were treated with vitreoretinal surgery, enucleation, or evisceration [14].
84 Throughout data collection and analysis, the investigators had access only to fully anonymized data and
85 no personal identifiable information. Given the retrospective nature of this study and the use of
86 anonymized data, the ethics committee waived the requirement for additional informed consent.

## 87 Participants and clinical evaluation

88 Patients with OGIs complicated by corneal blood staining were included. Exclusion criteria were
89 endophthalmitis, pre-existing ocular pathology, incomplete clinical or photographic records, follow-up
90 < 12 months, or enucleation/evisceration within 12 months (S1 Fig).
91 Preoperative data collected included age, sex, ocular history, best-corrected visual acuity (BCVA), IOP,
92 slit-lamp photographs, and anterior segment optical coherence tomography (AS-OCT). BCVA was
93 measured using a standard vision chart and converted into logarithm of the minimum angle of resolution
94 (logMAR). For statistical consistency, counting fingers, hand motion, light perception, and no light
95 perception (NLP) were converted to 1.87, 2.30, 2.80, and 4.00 logMAR, respectively. IOP was measured
96 using a rebound tonometer (iCare TA01i, Icare Finland Oy, Helsinki, Finland). AS-OCT (Wavelength
97 nanometer, Tomey CASIA2, Nagoya, Japan) was performed by trained clinicians (AD, HW, and SG),
98 with serial scans were obtained from same corneal regions whenever possible, using anatomical
99 landmarks and previous scan locations as references.
100 All patients underwent anterior chamber washout at the start of the initial PPV. Sufficient clarity for PPV
101 after washout was defined as intraoperative corneal clarity that allowed the surgeon to identify major
102 posterior segment structures and proceed safely without TKP. When safe visualization could not be
103 achieved, TKP-assisted PPV was performed, followed by replacement of the trephined autologous cornea
104 (TKP + PPV + RTC). The surgical procedure for TKP + PPV + RTC was described previously [15].
105 Follow-up duration varied among patients. BCVA, IOP, and slit-lamp photographs were recorded at each
106 visit. Additional treatments were provided as clinically indicated.
107 Postoperative corneal outcomes were categorized as complete clearing, partial clearing, or persistent
108 opacity. Complete clearing was defined as disappearance of opacity attributable to corneal blood staining
109 during follow-up, although other corneal changes such as scarring or band keratopathy could still be
110 present. Partial clearing was defined as a recognizable decrease in corneal blood staining compared with
111 baseline, with formation of a clear corneal area sufficient for clinical posterior segment assessment.
112 Persistent opacity was defined as residual opacity attributable to corneal blood staining with little or no
113 regression compared with baseline, continuing to limit corneal transparency or fundus visualization.
114 Serial slit-lamp photographs, fundus photographs when available, and clinical records were
115 independently reviewed by two ophthalmologists (KF and LH) to classify the corneal outcomes.
116 Disagreements were resolved by a senior ophthalmologist (ZZM).

## 117 Statistical analyses

118 Data were analyzed using SPSS software (version 26.0, IBM Corp., Armonk, NY, USA) and GraphPad
119 Prism 8 (GraphPad Software, San Diego, CA, USA). Descriptive statistics were reported as median
120 (interquartile range, IQR) for non-normally distributed variables or mean ± standard deviation for
121 normally distributed variables. Preoperative and postoperative parameters were compared using the
122 Wilcoxon matched-pairs signed rank test. A p-value of < 0.05 was considered statistically significant.

123	**Result**

124 A total of 3,152 eyes in the EIVS database were screened, including 1,920 eyes with open-globe injuries.
125 Among them, 157 eyes had documented corneal blood staining. After excluding 115 eyes because of
126 endophthalmitis, pre-existing ocular diseases, incomplete clinical or photographic records, follow-up
127 shorter than 12 months, or enucleation/evisceration within 12 months; 42 eyes (42 patients, 37 patients
128 [88.1%] were male; 16 right eyes) were included in the final analysis (S1Fig). The median age was 36.5
129 years (range, 3- 61 years). The mechanism of injury was globe rupture in 40 eyes (95.2%), penetrating
130 injury in 1 eye (2.4%), and intraocular foreign body (IOFB) in 1 eye (2.4%). The median interval from
131 trauma to surgery was 21.5 days (IQR, 15.0- 28.5 days).
132 Preoperative ocular hypotony (intraocular pressure [IOP] ≤10 mmHg) was present in 38 eyes (90.5%).
133 Severe posterior segment injury was common: choroidal detachment occurred in 40 eyes, retinal
134 detachment in 37 eyes, and no identifiable retinal tissue was noted in 5 eyes. All eyes received intraocular
135 tamponade with silicone oil (n = 40) or long-acting gas (n = 2). The median follow-up duration was 36
136 months (IQR, 23- 42 months; range, 12- 60 months). During follow-up, most eyes required additional
137 silicone oil procedures (e.g., oil refill, partial removal, or removal with re-tamponade), and complete
138 silicone oil removal was achieved in only 4 eyes by the final visit.
139 After anterior chamber washout, adequate corneal clarity to proceed with PPV was achieved in 8 eyes,
140 whereas the remaining 34 eyes required TKP + PPV + RTC. During follow-up, clearing of cornea blood
141 staining was observed 35/42 eyes. In the anterior chamber washout only group, complete clearing
142 occurred in 4 eyes and partial clearing in 4 eyes. In the TKP + PPV + RTC group, complete clearing
143 occurred in 9 eyes, partial clearing in 18 eyes, and persistent opacity in 7 eyes. The first documented
144 timing of corneal clearing is summarized in S1 Table. Complete or partial clearing formed a peripheral
145 “window” that permitted fundus assessment (Fig 1 a- e). At the final follow-up, persistent central opacity
146 or scarring was observed in 17 of 42 eyes, including band keratopathy (9/42), central corneal scarring,
147 and residual blood staining (Fig 1). Fig 2 presents serial AS-OCT images of a representative case from
148 the complete clearing group managed with anterior chamber washout only, showing gradual clearing of
149 corneal blood staining in this eye.

150

##### 151 Fig 1. Anterior segment photographs of corneal blood staining before and after clearing, with

152 **corresponding fundus views.**
153 This series focuses on corneal changes and the possibility for fundus assessment; concomitant intraocular
154 comorbidities were not considered. Each case is presented as paired panels, with the corresponding
155 fundus image displayed in the upper right corner (a–e).
156 (A, a) Immediate postoperative and postoperative month 6 (history of radial keratotomy). Vascularization
157 at periphery cornea can be seen.
158 (B, b) Postoperative months 2 and 17.
159 (C, c) Intraoperative and postoperative month 12.
160 (D, d) Postoperative months 6 and 24.
161 (E, e) Intraoperative and postoperative month 48.
162 (F, f) Preoperative and postoperative month 7, showing band keratopathy.
163 (G, g) Preoperative and postoperative day 1.
164 (H, h) Immediate postoperative and postoperative month 24, showing central corneal opacity.
165 (I, i) Postoperative day 1 and postoperative month 15.
166 (J, j) Preoperative and postoperative month 4.

##### 167 Fig 2. Slit-lamp photographs and AS-OCT demonstrating complete resolution of corneal blood

168 **staining.**
169 (A, D) Postoperative month 4; (B, E) postoperative month 10; (C, F) postoperative month 16.

170

171 Preoperatively, 28 eyes (66.7%) had NLP, which decreased to 12 eyes (28.6%) after surgery. At the final
172 follow-up, phthisis bulbi developed in 5 eyes. Baseline characteristics and perioperative outcomes are
173 summarized in Table 1. Only one eye underwent delayed allograft PKP. This was a gas-tamponaded eye
174 in a 46-year-old man with traumatic aniridia, aphakia after prior lens extraction in local hospital, vitreous
175 hemorrhage, and post-traumatic secondary glaucoma. Corneal clarity improved gradually and BCVA reached 0.5 at
176 14 months after the initial surgery, with well-controlled IOP, indicating favorable visual potential and a
177 relatively stable posterior segment. Allograft PKP combined with scleral-fixated IOL implantation was
178 therefore performed for optical rehabilitation and aphakia correction. Early post-PKP follow-up was
179 available, but the patient was subsequently lost to follow-up. Upon return for follow-up 3 years after PKP, the
180 eye had progressed to NLP due to glaucoma, with epithelial edema but relatively preserved stromal
181 clarity in corneal graft.

##### 182 Table 1 Patient demographics, preoperative/postoperative ocular findings

Patients Enrolled (n = 42)

Age, median (IQR)	(years)	36.5 (25.8- 49.3)

| Right eye, n (%) |  | 16 (38.1) |
| --- | --- | --- |
| Male, n (%) |  | 37 (88.1) |
| Surgery time interval, median (IQR) | (days) | 21.5 (15.0- 28.5) |
| Open globe injury, n (%) |  | 42 (100) |
| Rupture | | 40 (95.2) |
| Penetrating | | 1 (2.4) |
| Intraocular foreign bodies (IOFB) | | 1 (2.4) |
| Hypotony, n (%) | | 38 (90.5) |
| Retinal and choroidal condition, n (%) | |  |
| Choroidal detachment | | 40 (95.2) |
| Retinal detachment | | 37 (88.1) |
| No retina detected | | 5 (11.9) |
| Intraocular tamponade, n (%) | | 42 (100) |
| Silicone oil | | 40 (95.2) |

Long-acting gas	2 (4.8)

Balanced salt solution	0 (0) Cornea management, n (%)

TKP + PPV + RTC	34 (81.0)

After anterior chamber washout, corneal

transparency was sufficient for PPV

8 (19.0)

Follow-up duration, median (IQR)	(months)	36 (23- 42)

| NLP preoperatively, n (%) | 28 (66.7) |
| --- | --- |
| NLP postoperatively, n (%) | 12 (28.6) |
| Phthisis bulbi at the final follow-up, n (%) | 5 (11.9) |
| SOR at the final follow-up, n (%) | 4/40 (10) |
| Received PKP at the final follow-up, n (%) | 1 (2.4), tamponade with gas |
| Cornea outcome, n (%) |  |
| TKP + PPV + RTC Complete clearing | 9/34 (26.5) |
| Partial clearing | 18/34 (52.9) |
| Persistent opacity | 7/34 (20.6) |

Anterior chamber washout only

Complete clearing

4/8 (50)

Partial clearing	4/8 (50) Persistent opacity	0/8 (0)

185

186 Median BCVA improved from 4.00 logMAR preoperatively (IQR, 2.80- 4.00) to 3.40 logMAR on day 1
187 (IQR, 2.80- 4.00; p = 0.016) and 2.80 logMAR on day 7 (IQR, 2.80- 4.00; p = 0.002), as well as at the
188 final visit (median, 2.80 logMAR; IQR, 2.30- 4.00; p = 0.002). BCVA showed no significant difference
189 between day 7 and the final visit (p = 0.344) (Fig 3A).

190

191

##### 192 Fig 3. Changes in BCVA and IOP preoperative/postoperative.

193 (A) BCVA demonstrated significant improvement at D1, D7 and the final visit comparing with
194 preoperative. No significant improvement was observed at final visit when compared with D7 (n = 42).
195 (B) IOP significantly increased at D1, and dropped at D7 to preoperative level. At final visit, IOP showed
196 significant increase compared with pre-op, this process includes multiple silicone oil refilling in some
197 cases.
198 Median with IQR, Wilcoxon matched-pairs signed-rank test; *p < 0.05, **p < 0.01.
199 Abbreviations: Pre-op, preoperative; D1, postoperative day 1; D7, postoperative day 7.

200

201 Median IOP increased from 5.00 mmHg preoperatively (IQR, 4.00- 8.75) to 8.95 mmHg on day 1 (IQR,
202 5.50- 10.00; p = 0.019), measured 7.00 mmHg on day 7 (IQR, 6.25- 10.00, p = 0.401), and reached 10.00
203 mmHg at the final visit (IQR, 5.50- 11.00, p = 0.002) (Fig 3B).

204	**Discussion**

205 Management of corneal blood staining in the setting of complex ocular trauma remains challenging. In
206 this series, we applied a modified approach (anterior chamber washout first, then TKP + PPV + RTC if
207 needed) instead of performing immediate allogeneic PKP. A key observation was that corneal blood
208 staining frequently showed spontaneous partial-to-complete resolution after surgery, often providing a
209 peripheral “window” that permitted postoperative fundus assessment (Fig 1a- e). For eyes with corneal
210 opacity that limited direct visualization, including during the early postoperative period (e.g., at 1 week),
211 posterior segment assessment was performed using B-scan ultrasonography, visual function
212 examinations, and IOP measurement. The decision to perform subsequent PKP is strictly based on a
213 comprehensive evaluation of both visual function and the anatomical viability of the injured eye. Only
214 one eye ultimately underwent PKP. These findings support a conservative corneal strategy in eyes with
215 limited visual potential and high graft-risk profiles. Conventionally, surgery has relied on immediate
216 allogeneic PKP following TKP-assisted PPV. However, this strategy exposes the corneal graft to
217 pathophysiologic stressors that are common in severely injured eyes, and may increase exposure to
218 prolonged postoperative corticosteroid or immunosuppressive therapy as well as the risk of graft
219 rejection in eyes with limited visual potential.
220 OGIs often leads to persistent hypotony and silicone-oil sustained [15, 16]. In OGIs, hypotony can arise
221 from direct ciliary body injury, cyclodialysis, ischemia and/or membrane formation of ciliary body,
222 leading to reduced aqueous humor production and/or increased outflow through the suprachoroidal space
223 [17-19]. Under these conditions, impaired aqueous circulation compromises corneal metabolic support,
224 while sustained contact between silicone oil and the corneal endothelium accelerates endothelial
225 dysfunction [20]. Together, these factors increase the risk of graft decompensation and failure after PKP
226 [21].
227 Inflammation is another problem. Eyes with OGIs are commonly in an active inflammatory state at the
228 time of PPV, which is a well-established independent risk factor for graft failure [22]. Inflammation
229 induces upregulation of major histocompatibility complex (MHC) class I and class II antigen expression
230 [23] and promotes vascular endothelial growth factor production, thereby facilitating graft rejection and
231 corneal neovascularization [24]. Consistent with these mechanisms, Williams et al. showed that past
232 ocular inflammation increased the risk of graft failure by 4.40-fold, while active inflammation increased
233 the risk to 9.6-fold [25]. Accordingly, delaying PKP until inflammation has stabilized may reduce the
234 risk of failure. The clinical importance of PKP timing has been supported by prior studies; for example,
235 Roters et al. reported a significantly lower rate of graft failure when PKP was performed after 8 weeks
236 of trauma [21]. Although delaying surgery may allow neovascularization to develop, as shown in Fig 1a,
237 resulting in a vascularized graft bed. This trade-off may be acceptable, as the associated risk (odds ratio,
238 2.74 [25]) is substantially lower than that posed by active inflammation.
239 Importantly, the overall prognosis of OGIs is often poor [15]. All eyes in our series had severe vision-
240 threatening pathology, including choroidal detachment, retinal detachment, or retinal tissue loss. Twenty-
241 eight eyes presented with no light perception (NLP), and 12 remained NLP after surgery. During follow-
242 up, 11.9% progressed to phthisis, indicating irreversible anatomical failure. In addition, silicone oil
243 removal, which is critical for graft survival, was achieved in only 10% of our cases. Under these
244 conditions, corneal transplantation may offer limited functional benefit in many eyes. Other global
245 assessment of ocular viability—such as IOP trends, residual light perception, and B scan findings—may
246 be more informative in guiding management decisions [26-28]. Consistent with this reality, only one eye
247 underwent PKP in our study, suggesting that most patients were poor candidates for primary keratoplasty;
248 for these individuals, TKP + PPV + RTC represents a more cost-effective and practical strategy. This
249 selective approach is also vital when considering the critical global shortage of donor tissue, where only
250 one cornea is available for every 70 patients in need [29].
251 The natural course of corneal blood staining further supports a conservative surgical strategy. Several
252 case reports have described spontaneous resolution of corneal blood staining [30, 31]. Clearance typically
253 progresses from the peripheral cornea toward the center and from posterior to anterior layers [31],
254 consistent with our observations. This pattern has been attributed to limbal circulation and shedding of
255 iron-containing epithelial cells [8]. Conjunctival flap coverage may further accelerate this process [32],
256 possibly by increasing local blood supply. In our series, most eyes showed varying degrees of
257 spontaneous clearance during follow-up, which enabled posterior segment assessment through this
258 window. Fig 2 illustrates one case with complete clearance, the AS-OCT result showed a progressive
259 reduction and eventual disappearance of hyperreflective stromal opacity.
260 Although corneal blood staining may clear spontaneously, this process can take months to years [5, 30-
261 32]. In contrast, the timing of PPV after severe ocular trauma is critical, as delayed intervention may
262 increase the risk of proliferative vitreoretinopathy and lead to irreversible anatomical or functional loss
263 [2, 33, 34]. Therefore, timely posterior segment access while avoiding unnecessary high-risk allograft
264 PKP in the acute traumatic setting is essential. Our strategy addresses this dilemma by performing
265 anterior chamber washout first, followed by TKP + PPV + RTC when visualization remains inadequate.
266 Compared with conventional TKP-assisted PPV followed by immediate allograft PKP, this approach
267 avoids placing donor tissue into an acutely inflamed and often silicone-oil-filled eye. Compared with
268 endoscopy-assisted vitrectomy, it preserves a conventional wide-field microscopic surgical view and
269 does not require specialized endoscopic equipment or a separate technical learning. Thus, this approach
270 may provide a practical alternative for managing severe OGIs with corneal blood staining, particularly
271 in centers where endoscopic vitreoretinal surgery is not routinely available.

## 272 Limitation

273 This study has several limitations. First, it was a retrospective analysis of a small cohort with highly
274 heterogeneous severe ocular trauma, including variability in injury mechanism, posterior segment
275 pathology, tamponade status, and follow-up duration. These factors, together with differences in the
276 severity of corneal blood staining and corneal tissue damage, may have influenced the timing and extent
277 of corneal clearing. Second, follow-up visits and AS-OCT imaging were not performed at standardized
278 intervals. Therefore, the exact onset of corneal clearing could not be determined for every eye, and
279 quantitative cohort-level AS-OCT analyses were not feasible. The analysis provided in the supplementary
280 materials should be interpreted cautiously because of the limited sample size and substantial baseline
281 imbalance. Finally, in this cohort of severe OGIs, final visual and anatomical outcomes were largely
282 determined by posterior segment damage and overall ocular viability. Thus, the independent contribution
283 of corneal blood staining to final BCVA, phthisis bulbi, or complete silicone oil removal could not be
284 reliably isolated. Larger, prospective studies with standardized imaging, clinical grading, and follow-up
285 protocols are needed to better define predictors of spontaneous clearance and to refine timing and
286 selection of subsequent keratoplasty.

287	**Conclusion**

288	This conservative corneal strategy provides timely posterior segment access in severe OGIs complicated

289	by corneal blood staining while preserving the globe and conserving corneal tissue. By avoiding routine

290	immediate allograft PKP in the acute traumatic setting, it allows subsequent keratoplasty decisions to be

291	guided by ocular stability, corneal status, and visual potential. These findings support selective, rather

292	than routine, allograft PKP in such severely injured eyes.

293	**Acknowledgement**

294	This project has no funding support.

# 295 Conflict of Interest

296	None of the authors have any conflicting interests to disclose.

# 297 Author Contribution Statement

298 ZZM conceived the study and developed the surgical approach. ZZM, YL, HC, KF, and LH performed
299 the surgical procedures. BW conducted data analysis and drafted the manuscript. BW, AD, HW, and SG
300 contributed to data collection. All authors reviewed and approved the final version of the manuscript.

301	**Reference**

302 1. Colyer MH, Chun DW, Bower KS, Dick JS, Weichel ED. Perforating globe injuries during operation
303 Iraqi Freedom. Ophthalmology. 2008;115(11):2087-93.e2.
304 2. Miller SC, Fliotsos MJ, Justin GA, Yonekawa Y, Chen A, Hoskin AK, et al. Global current practice
305 patterns for the management of open globe injuries. Am J Ophthalmol. 2022;234:259-73.
306 3. Ryan DS, Sia RK, Colyer M, Stutzman RD, Wroblewski KJ, Mines MJ, et al. Anterior segment
307 imaging in combat ocular trauma. J Ophthalmol. 2013;2013:308259.
308 4. McDonnell PJ, Gritz DC, McDonnell JM, Zarbin MA. Fluorescence of blood-stained cornea. Cornea.
309 1991;10(5):445-9.
310 5. Brodrick JD. Corneal blood staining after hyphaema. Br J Ophthalmol. 1972;56(8):589-93.
311 6. Beyer TL, Hirst LW. Corneal blood staining at low pressures. Arch Ophthalmol. 1985;103(5):654-5.
312 7. Mansoori T, Srirampur A. Anterior segment optical coherence tomography findings in corneal blood
313 staining. J Curr Ophthalmol. 2021;33(1):95.
314 8. McDonnell PJ, Green WR, Stevens RE, Bargeron CB, Riquelme JL. Blood staining of the cornea:
315 light microscopic and ultrastructural features. Ophthalmology. 1985;92(12):1668-74.
316 9. Guevara-Villarreal DA, Rodríguez-Valdés PJ. Posterior segment intraocular foreign body: extraction
317 surgical techniques, timing, and indications for vitrectomy. J Ophthalmol. 2016;2016:2034509.
318 10. Fasolo A, Capuzzo C, Fornea M, Franch A, Birattari F, Carito G, et al. Risk factors for graft failure
319 after penetrating keratoplasty: 5-year follow-up from the corneal transplant epidemiological study.
320 Cornea. 2011;30(12):1328-35.
321 11. Dave VP, Tyagi M, Narayanan R, Pappuru RR. Intraocular endoscopy: a review. Indian J Ophthalmol.
322 2021;69(1):14-25.
323 12. Ajlan RS, Desai AA, Mainster MA. Endoscopic vitreoretinal surgery: principles, applications and
324 new directions. Int J Retina Vitreous. 2019;5:15.
325 13. Abouodah H, Hamada K, Pfannenstiel M, Ajlan R. Endoscopic pars plana vitrectomy in patients with
326 open-globe injury and corneal opacity. J Vitreoretin Dis. 2023;7(2):103-8.
327 14. Feng K, Hu YT, Ma Z. Prognostic indicators for no light perception after open-globe injury: eye
328 injury vitrectomy study. Am J Ophthalmol. 2011;152(4):654-62.e2.
329 15. Chen HJ, Wang CG, Dou HL, Feng XF, Feng K, Hu YT, et al. Anatomical outcome of vitreoretinal
330 surgery using temporary keratoprosthesis and replacement of the trephined corneal button for severe
331 open globe injuries: one-year result. J Ophthalmol. 2014;2014:794039.
332 16. Pfister N, Dormegny L, Ballonzoli L, Sauer A, Speeg-Schatz C, Bourcier T, et al. Long-term
333 microvascular remodeling and cystic changes after retinal detachment treated with silicon oil tamponade.
334 Retina. 2023;43(6):923-31.
335 17. Chen B, Wang GX, Zhang X, Yang H. Scleral buckling combined with internal cyclopexy for severe
336 traumatic cyclodialysis cleft in open globe injuries. Int J Ophthalmol. 2019;12(10):1649-53.
337 18. Rizzo S, Barale PO, Ayello-Scheer S, Devenyi RG, Delyfer MN, Korobelnik JF, et al. Hypotony and
338 the Argus II retinal prosthesis: causes, prevention and management. Br J Ophthalmol. 2020;104(4):518-
339 23.
340 19. Faude F, Wiedemann P. Vitreoretinal endoscope for the assessment of the peripheral retina and the
341 ciliary body after large retinectomies in severe anterior PVR. Int Ophthalmol. 2004;25(1):53-6.
342 20. Yang CS, Chen KH, Hsu WM, Li YS. Cytotoxicity of silicone oil on cultivated human corneal
343 endothelium. Eye. 2008;22(2):282-8.
344 21. Roters S, Szurman P, Hermes S, Thumann G, Bartz-Schmidt KU, Kirchhof B. Outcome of combined
345 penetrating keratoplasty with vitreoretinal surgery for management of severe ocular injuries. Retina.
346 2003;23(1):48-56.
347 22. Coster DJ, Williams KA. The impact of corneal allograft rejection on the long-term outcome of
348 corneal transplantation. Am J Ophthalmol. 2005;140(6):1112-22.
349 23. Pepose JS, Gardner KM, Nestor MS, Foos RY, Pettit TH. Detection of HLA class I and II antigens in
350 rejected human corneal allografts. Ophthalmology. 1985;92(11):1480-4.
351 24. Cursiefen C, Chen L, Borges LP, Jackson D, Cao J, Radziejewski C, et al. VEGF-A stimulates
352 lymphangiogenesis and hemangiogenesis in inflammatory neovascularization via macrophage
353 recruitment. J Clin Invest. 2004;113(7):1040-50.
354 25. Williams KA, Roder D, Esterman A, Muehlberg SM, Coster DJ. Factors predictive of corneal graft
355 survival: report from the Australian Corneal Graft Registry. Ophthalmology. 1992;99(3):403-14.
356 26. Zhou Y, DiSclafani M, Jeang L, Shah AA. Open globe injuries: review of evaluation, management,
357 and surgical pearls. Clin Ophthalmol. 2022;16:2545-59.
358 27. Kylstra JA, Lamkin JC, Runyan DK. Clinical predictors of scleral rupture after blunt ocular trauma.
359 Am J Ophthalmol. 1993;115(4):530-5.
360 28. Ding C, Zeng J. Clinical study on hypotony following blunt ocular trauma. Int J Ophthalmol.
361 2012;5(6):771-3.
362 29. Gain P, Jullienne R, He Z, Aldossary M, Acquart S, Cognasse F, et al. Global survey of corneal
363 transplantation and eye banking. JAMA Ophthalmol. 2016;134(2):167-73.
364 30. Hwang BE, Ahn YJ, Shin SY, Park SH. Serial photographic monitoring of spontaneous clearance of
365 corneal blood stain in a child with traumatic hyphema. Korean J Ophthalmol. 2017;31(3):281-2.
366 31. Lin HY, Peng KL. Gradual spontaneous resolution of corneal bloodstaining after anterior chamber
367 irrigation for rebleeding secondary to traumatic hyphema. Int Med Case Rep J. 2018;11:239-42.
368 32. Yang X, Zhou Q, Du S. Conjunctival flap covering in the treatment of corneal blood staining. Can J
369 Ophthalmol. 2011;46(5):442-3.
370 33. Ozdek S, Hasanreisoglu M, Yuksel E. Chorioretinectomy for perforating eye injuries. Eye.
371 2013;27(6):722-7.
372 34. Chauhan MZ, Georgiou M, Al-Hindi H, Uwaydat SH. Outcomes of pars plana vitrectomy following

373

374

ocular trauma at varying surgical time points. Int J Retina Vitreous. 2022;8(1):49.

# 375 Supporting information

##### 376 S1 Fig. Flow diagram of the study population enrollment from the EIVS database.

377 **S1 Table. The first documented timing for corneal clearing.** The timing for corneal clearing varies
378 widely among individual patients, as it depends on the severity and extent of the pre-operative opacity,
379 as well as the severity of the ocular trauma. Given the retrospective nature of this cohort, follow-up
380 visits were not scheduled at uniform intervals; thus the exact onset of corneal clearing could not be
381 precisely determined for every eye, and only the timing of actual follow-up visits could be verified.
382 These data should be interpreted cautiously.

##### 383 S2 Table. Final BCVA, final IOP, and complete silicone oil removal in the two groups. In this

384 cohort of severe open-globe injuries, final visual and anatomical outcomes were largely determined by
385 posterior segment damage and overall ocular viability. Therefore, the independent contribution of
386 corneal blood staining to global outcomes, such as final BCVA, final IOP, or complete silicone oil
387 removal, could not be reliably isolated in this retrospective dataset. Given the small sample size and the
388 highly imbalanced distribution of injury severity, including choroidal detachment, retinal detachment,
389 retinal tissue loss, hypotony, and tamponade type, multivariable prediction modeling would be
390 statistically underpowered and could lead to potentially misleading conclusions. These data should be

391

392

393

394

395

396

interpreted cautiously.

Figure	[Click here to access/download;Figure;Fig1.tif](https://www2.cloud.editorialmanager.com/pone/download.aspx?id=44823306&guid=1b35f658-6207-42dc-b01b-08285e284025&scheme=1)[<https://www2.cloud.editorialmanager.com/pone/download.aspx?id=44823306&guid=1b35f658-6207-42dc-b01b-08285e284025&scheme=1>](https://www2.cloud.editorialmanager.com/pone/download.aspx?id=44823306&guid=1b35f658-6207-42dc-b01b-08285e284025&scheme=1)

Figure	[Click here to access/download;Figure;Fig2.tif](https://www2.cloud.editorialmanager.com/pone/download.aspx?id=44823307&guid=a784c4b0-0263-4ca1-8a21-ac0d64d1b21c&scheme=1)[<https://www2.cloud.editorialmanager.com/pone/download.aspx?id=44823307&guid=a784c4b0-0263-4ca1-8a21-ac0d64d1b21c&scheme=1>](https://www2.cloud.editorialmanager.com/pone/download.aspx?id=44823307&guid=a784c4b0-0263-4ca1-8a21-ac0d64d1b21c&scheme=1)

Figure	[Click here to access/download;Figure;Fig3.tif](https://www2.cloud.editorialmanager.com/pone/download.aspx?id=44823308&guid=dadbeb86-c6f2-4dd3-9dc7-2ddfdf31ccc1&scheme=1)[<https://www2.cloud.editorialmanager.com/pone/download.aspx?id=44823308&guid=dadbeb86-c6f2-4dd3-9dc7-2ddfdf31ccc1&scheme=1>](https://www2.cloud.editorialmanager.com/pone/download.aspx?id=44823308&guid=dadbeb86-c6f2-4dd3-9dc7-2ddfdf31ccc1&scheme=1)

Supporting Information

[Click here to access/download](https://www2.cloud.editorialmanager.com/pone/download.aspx?id=44823309&guid=be3c27ca-ba99-48b5-af58-e327feba591b&scheme=1)

[**Supporting Information** S1_Fig.pdf](https://www2.cloud.editorialmanager.com/pone/download.aspx?id=44823309&guid=be3c27ca-ba99-48b5-af58-e327feba591b&scheme=1)

Supporting Information

[Click here to access/download](https://www2.cloud.editorialmanager.com/pone/download.aspx?id=44823310&guid=7e58c5f2-33b9-4971-976a-20b90d942215&scheme=1)

[**Supporting Information** S1_Table.pdf](https://www2.cloud.editorialmanager.com/pone/download.aspx?id=44823310&guid=7e58c5f2-33b9-4971-976a-20b90d942215&scheme=1)

Supporting Information

[Click here to access/download](https://www2.cloud.editorialmanager.com/pone/download.aspx?id=44823311&guid=cdbba7a1-b8f1-45c4-b22a-0d0b7f77247d&scheme=1)

[**Supporting Information** S2_Table.pdf](https://www2.cloud.editorialmanager.com/pone/download.aspx?id=44823311&guid=cdbba7a1-b8f1-45c4-b22a-0d0b7f77247d&scheme=1)

Revised Manuscript with Track Changes

1 **Clinical course and outcomes of corneal blood staining in open-globe injury: Eye Injury**
2 **Vitrectomy Study**
3 Bingjie Wang^1,2^, Kang Feng ^1^, Yao Lu ^1^, Huijin Chen^1^, Liang Han^1^, Aihua Ding^1^, Haikun Wang^1^,
4 Shaofeng Gu^1^, Zhizhong Ma ^1*^
5 ^1^Department of Ophthalmology, Peking University Third Hospital, Beijing, China
6 ^2^Eye Center, Beijing Tsinghua Changgung Hospital, School of Clinical Medicine, Tsinghua
7 University, Beijing, China

8

9

##### 10 Key message:

11 What is known
12 1. Corneal blood staining in open-globe injuries (OGIs) traditionally requires immediate allograft
13 penetrating keratoplasty (PKP) to enable posterior segment access.
14 2. Immediate PKP in the acute phase is high-risk due to intense inflammation, hypotony, and long-term
15 silicone oil tamponade.
16 What is new
17 1. This study highlights a globe-preserving and cornea-conserving strategy for severe OGIs complicated
18 by corneal blood staining, using anterior chamber washout first, and TKP-assisted PPV with replacement
19 of the trephined autologous cornea when necessary.
20 2. By avoiding routine immediate allograft PKP in the acute traumatic setting, this approach preserves
21 the opportunity for later keratoplasty to be selected after ocular stabilization, based on overall ocular
22 status, corneal condition, and visual potential, while reducing graft-related risks.

23

24	**Abstract**

25 Purpose: To describe the clinical course and outcomes of corneal blood staining in open-globe injuries
26 (OGIs) after vitrectomy without routine immediate allograft penetrating keratoplasty (PKP).
27 Methods: This study included 42 eyes of 42 patients with corneal blood staining secondary to OGIs.
28 Clinical data were retrospectively collected from the Eye Injury Vitrectomy Study (EIVS). All eyes
29 underwent anterior chamber washout at the start of pars plana vitrectomy (PPV). When visualization
30 remained inadequate, temporary keratoprosthesis (TKP)-assisted PPV was performed, followed by
31 replacement of the trephined autologous cornea (TKP + PPV + RTC). Postoperative outcomes, including
32 corneal transparency, best-corrected visual acuity (BCVA), intraocular pressure (IOP), and need for
33 subsequent PKP, were assessed.
34 Results: Median age was 36.5 years; globe rupture accounted for 95.2%. Preoperative hypotony (IOP
35 ≤10 mmHg) was present in 90.5%, with severe posterior segment injury in all eyes. Eight eyes achieved
36 sufficient corneal clarity after washout alone; the remaining 34 required TKP + PPV + RTC. During
37 follow-up, varying degrees of spontaneous clearance occurred in most eyes, forming a peripheral
38 “window” that enabled fundus assessment. At the final follow-up, BCVA improved from median 4.00
39 logMAR preoperatively to 2.80 logMAR (p = 0.002), and median IOP rose from 5.00 to 10.00 mmHg (p

40	= 0.002). Five eyes developed phthisis bulbi, four eyes achieved complete silicone oil removal, and only

41 1 eye underwent allograft PKP.
42 Conclusions: The strategy of anterior chamber washout first, followed by TKP + PPV + RTC when
43 necessary, provides timely posterior segment access while preserving the globe and conserving corneal
44 tissue in severe OGIs complicated by corneal blood staining. These findings support selective, rather
45 than routine, PKP during follow-up.
46 **Keywords:** open-globe injury, corneal blood staining, temporary keratoprosthesis, penetrating
47 keratoplasty, vitrectomy

# 48 Introduction

49 Open-globe injuries (OGIs) are ocular injuries associated with the worst visual outcomes and may result
50 in monocular or bilateral blindness, particularly in eyes with globe rupture [1, 2]. Adequate corneal
51 transparency is essential for visualization of posterior segment pathology. In severe ocular trauma,
52 however, corneal transparency may be compromised, limiting clinical assessment and impeding surgical
53 repair.
54 Among trauma-related corneal opacities, corneal blood staining is one of the most challenging conditions.
55 It is characterized by deposition of hemoglobin and its breakdown products within the corneal stroma
56 [3], most commonly following prolonged hyphema and elevation of intraocular pressure (IOP) [4, 5],
57 but it has also been reported in eyes with low IOP [6, 7]. Disruption of endothelial function, stromal
58 impregnation by erythrocyte derivatives, and impaired metabolic clearance contribute to its development
59 [8].
60 In eyes with corneal blood staining requiring pars plana vitrectomy (PPV), a temporary keratoprosthesis
61 (TKP) is commonly used to facilitate posterior segment visualization [9]. This approach, however,
62 necessitates donor tissue for subsequent allograft penetrating keratoplasty (PKP) and is associated with
63 risks of graft rejection and infection related to long-term immunosuppression [10]. Furthermore, given
64 the poor prognosis in eyes with extensive posterior segment damage, the indication for immediate PKP
65 may be uncertain. Although endoscopy-assisted vitrectomy has been proposed as an alternative, its
66 adoption is limited by low image resolution, restricted field of view, specialized equipment requirements,
67 and a steep learning curve [11-13].
68 In this study, we evaluated a modified corneal conservative surgical strategy for OGIs complicated by
69 corneal blood staining, and analyzed anatomical and functional outcomes, as well as the evolution of
70 corneal blood staining.

71	**Methods**

## 72 Human ethics and consent to participate declarations

73 This study was approved by the Institutional Review Board of Peking University Third Hospital Medical
74 Science Research Ethics Committee (Ethics No. IRB00006761-2012060) and was conducted in
75 accordance with the Declaration of Helsinki. Clinical data were collected from the Eye Injury Vitrectomy
76 Study (EIVS). The trial was registered with the Chinese Clinical Trial Registry (ChiCTR; Number:
77 ChiCTR-OCH-08000199) on November 28, 2008. Written informed consent was obtained from all
78 participants at the time of enrollment in the EIVS, including consent for the use of non-personally
79 identifiable medical records for future research. For participants younger than 18 years of age, written
80 informed consent was obtained from their parents or legal guardians. The present study represents a
81 retrospective analysis of EIVS data from Peking University Third Hospital between June 2012 and June
82 2025. As defined by the EIVS database, the general inclusion criteria were patients who suffered from
83 severe eye injury and were treated with vitreoretinal surgery, enucleation, or evisceration [14].
84 Throughout data collection and analysis, the investigators had access only to fully anonymized data and
85 no personal identifiable information. Given the retrospective nature of this study and the use of
86 anonymized data, the ethics committee waived the requirement for additional informed consent.

## 87 Participants and clinical evaluation

88 Patients with OGIs complicated by corneal blood staining were included. Exclusion criteria were
89 endophthalmitis, pre-existing ocular pathology, incomplete clinical or photographic records, follow-up
90 < 12 months, or enucleation/evisceration within 12 months (S1 Fig).
91 Preoperative data collected included age, sex, ocular history, best-corrected visual acuity (BCVA), IOP,
92 slit-lamp photographs, and anterior segment optical coherence tomography (AS-OCT). BCVA was
93 measured using a standard vision chart and converted into logarithm of the minimum angle of resolution
94 (logMAR). For statistical consistency, counting fingers, hand motion, light perception, and no light
95 perception (NLP) were converted to 1.87, 2.30, 2.80, and 4.00 logMAR, respectively. IOP was measured
96 using a rebound tonometer (iCare TA01i, Icare Finland Oy, Helsinki, Finland). AS-OCT (Wavelength
97 nanometer, Tomey CASIA2, Nagoya, Japan) was performed by trained clinicians (AD, HW, and SG),
98 with serial scans obtained from same corneal regions whenever possible, using anatomical
99 landmarks and previous scan locations as references.
100 All patients underwent anterior chamber washout at the start of the initial PPV. Sufficient clarity for PPV
101 after washout was defined as intraoperative corneal clarity that allowed the surgeon to identify major
102 posterior segment structures and proceed safely without TKP. When safe visualization could not be
103 achieved, TKP-assisted PPV was performed, followed by replacement of the trephined autologous cornea
104 (TKP + PPV + RTC). The surgical procedure for TKP + PPV + RTC was described previously [15].
105 Follow-up duration varied among patients. BCVA, IOP, and slit-lamp photographs were recorded at each
106 visit. Additional treatments were provided as clinically indicated.
107 Postoperative corneal outcomes were categorized as complete clearing, partial clearing, or persistent
108 opacity. Complete clearing was defined as disappearance of opacity attributable to corneal blood staining
109 during follow-up, although other corneal changes such as scarring or band keratopathy could still be
110 present. Partial clearing was defined as a recognizable decrease in corneal blood staining compared with
111 baseline, with formation of a clear corneal area sufficient for clinical posterior segment assessment.
112 Persistent opacity was defined as residual opacity attributable to corneal blood staining with little or no
113 regression compared with baseline, continuing to limit corneal transparency or fundus visualization.
114 Serial slit-lamp photographs, fundus photographs when available, and clinical records were
115 independently reviewed by two ophthalmologists (KF and LH) to classify the corneal outcomes.
116 Disagreements were resolved by a senior ophthalmologist (ZZM).

## 117 Statistical analyses

118 Data were analyzed using SPSS software (version 26.0, IBM Corp., Armonk, NY, USA) and GraphPad
119 Prism 8 (GraphPad Software, San Diego, CA, USA). Descriptive statistics were reported as median
120 (interquartile range, IQR) for non-normally distributed variables or mean ± standard deviation for
121 normally distributed variables. Preoperative and postoperative parameters were compared using the
122 Wilcoxon matched-pairs signed rank test. A p-value of < 0.05 was considered statistically significant.

123	**Result**

124 A total of 3,152 eyes in the EIVS database were screened, including 1,920 eyes with open-globe injuries.
125 Among them, 157 eyes had documented corneal blood staining. After excluding 115 eyes because of
126 endophthalmitis, pre-existing ocular diseases, incomplete clinical or photographic records, follow-up
127 shorter than 12 months, or enucleation/evisceration within 12 months; 42 eyes (42 patients, 37 patients
128 [88.1%] were male; 16 right eyes) were included in the final analysis (S1Fig). The median age was 36.5
129 years (range, 3- 61 years). The mechanism of injury was globe rupture in 40 eyes (95.2%), penetrating
130 injury in 1 eye (2.4%), and intraocular foreign body (IOFB) in 1 eye (2.4%). The median interval from
131 trauma to surgery was 21.5 days (IQR, 15.0- 28.5 days).
132 Preoperative ocular hypotony (intraocular pressure [IOP] ≤10 mmHg) was present in 38 eyes (90.5%).
133 Severe posterior segment injury was common: choroidal detachment occurred in 40 eyes, retinal
134 detachment in 37 eyes, and no identifiable retinal tissue was noted in 5 eyes. All eyes received intraocular
135 tamponade with silicone oil (n = 40) or long-acting gas (n = 2). The median follow-up duration was 36
136 months (IQR, 23- 42 months; range, 12- 60 months). During follow-up, most eyes required additional
137 silicone oil procedures (e.g., oil refill, partial removal, or removal with re-tamponade), and complete
138 silicone oil removal was achieved in only 4 eyes by the final visit.
139 After anterior chamber washout, adequate corneal clarity to proceed with PPV was achieved in 8 eyes,
140 whereas the remaining 34 eyes required TKP + PPV + RTC. During follow-up, clearing of cornea blood
141 staining was observed in 35/42 eyes. In the anterior chamber washout only group, complete clearing
142 occurred in 4 eyes and partial clearing in 4 eyes. In the TKP + PPV + RTC group, complete clearing
143 occurred in 9 eyes, partial clearing in 18 eyes, and persistent opacity in 7 eyes. The first documented
144 timing of corneal clearing is summarized in S1 Table. Complete or partial clearing formed a peripheral
145 “window” that permitted fundus assessment (Fig 1 a- e). At the final follow-up, persistent central opacity
146 or scarring was observed in 17 of 42 eyes, including band keratopathy (9/42), central corneal scarring,
147 and residual blood staining (Fig 1). Fig 2 presents serial AS-OCT images of a representative case from
148 the complete clearing group managed with anterior chamber washout only, showing gradual clearing of
149 corneal blood staining in this eye.

150

##### 151 Fig 1. Anterior segment photographs of corneal blood staining before and after clearing, with

152 **corresponding fundus views.**
153 This series focuses on corneal changes and the possibility for fundus assessment; concomitant intraocular
154 comorbidities were not considered. Each case is presented as paired panels, with the corresponding
155 fundus image displayed in the upper right corner (a–e).
156 (A, a) Immediate postoperative and postoperative month 6 (history of radial keratotomy). Vascularization
157 at periphery cornea can be seen.
158 (B, b) Postoperative months 2 and 17.
159 (C, c) Intraoperative and postoperative month 12.
160 (D, d) Postoperative months 6 and 24.
161 (E, e) Intraoperative and postoperative month 48.
162 (F, f) Preoperative and postoperative month 7, showing band keratopathy.
163 (G, g) Preoperative and postoperative day 1.
164 (H, h) Immediate postoperative and postoperative month 24, showing central corneal opacity.
165 (I, i) Postoperative day 1 and postoperative month 15.
166 (J, j) Preoperative and postoperative month 4.

##### 167 Fig 2. Slit-lamp photographs and AS-OCT demonstrating complete resolution of corneal blood

168 **staining.**
169 (A, D) Postoperative month 4; (B, E) postoperative month 10; (C, F) postoperative month 16.

170

171 Preoperatively, 28 eyes (66.7%) had NLP, which decreased to 12 eyes (28.6%) after surgery. At the final
172 follow-up, phthisis bulbi developed in 5 eyes. Baseline characteristics and perioperative outcomes are
173 summarized in Table 1. Only one eye underwent delayed allograft PKP. This was a gas-tamponaded eye
174 in a 46-year-old man with traumatic aniridia, aphakia after prior lens extraction in local hospital, vitreous
175 hemorrhage, and post-traumatic secondary glaucoma. Although preoperative B-scan suggested choroidal
176 detachment, this finding were not confirmed intraoperatively, and laser photocoagulation was applied
177 around the retinal break after retinal attachment. The RTC gradually clearing, and BCVA reached 0.5 at
178 14 months after the initial surgery, with well-controlled IOP, indicating favorable visual potential and a
179 relatively stable posterior segment. Allograft PKP combined with scleral-fixated IOL implantation was
180 therefore performed for optical rehabilitation and aphakia correction. Early post-PKP follow-up was
181 available, but the patient was subsequently lost to follow-up. When he returned 3 years after PKP, the
182 eye had progressed to NLP due to glaucoma, with epithelial edema but relatively preserved stromal
183 clarity in corneal graft.

##### 184 Table 1 Patient demographics, preoperative/postoperative ocular findings

Patients Enrolled (n = 42)

Age, median (IQR)	(years)	36.5 (25.8- 49.3)

| Right eye, n (%) |  | 16 (38.1) |
| --- | --- | --- |
| Male, n (%) |  | 37 (88.1) |
| Surgery time interval, median (IQR) | (days) | 21.5 (15.0- 28.5) |
| Open globe injury, n (%) |  | 42 (100) |
| Rupture | | 40 (95.2) |
| Penetrating | | 1 (2.4) |
| Intraocular foreign bodies (IOFB) | | 1 (2.4) |
| Hypotony, n (%) | | 38 (90.5) |
| Retinal and choroidal condition, n (%) | |  |
| Choroidal detachment | | 40 (95.2) |
| Retinal detachment | | 37 (88.1) |
| No retina detected | | 5 (11.9) |
| Intraocular tamponade, n (%) | | 42 (100) |
| Silicone oil | | 40 (95.2) |

Long-acting gas	2 (4.8)

Balanced salt solution	0 (0) Cornea management, n (%)

TKP + PPV + RTC	34 (81.0)

After anterior chamber washout, corneal

transparency was sufficient for PPV

8 (19.0)

Follow-up duration, median (IQR)	(months)	36 (23- 42)

| NLP preoperatively, n (%) | 28 (66.7) |
| --- | --- |
| NLP postoperatively, n (%) | 12 (28.6) |
| Phthisis bulbi at the final follow-up, n (%) | 5 (11.9) |
| SOR at the final follow-up, n (%) | 4/40 (10) |
| Received PKP at the final follow-up, n (%) | 1 (2.4), tamponade with gas |
| Cornea outcome, n (%) |  |
| TKP + PPV + RTC Complete clearing | 9/34 (26.5) |
| Partial clearing | 18/34 (52.9) |
| Persistent opacity | 7/34 (20.6) |

Anterior chamber washout only

Complete clearing

4/8 (50)

Partial clearing	4/8 (50) Persistent opacity	0/8 (0)

185

186 Median BCVA improved from 4.00 logMAR preoperatively (IQR, 2.80- 4.00) to 3.40 logMAR on day 1
187 (IQR, 2.80- 4.00; p = 0.016) and 2.80 logMAR on day 7 (IQR, 2.80- 4.00; p = 0.002), as well as at the
188 final visit (median, 2.80 logMAR; IQR, 2.30- 4.00; p = 0.002). BCVA showed no significant difference
189 between day 7 and the final visit (p = 0.344) (Fig 3A).

190

191

##### 192 Fig 3. Changes in BCVA and IOP preoperative/postoperative.

193 (A) BCVA demonstrated significant improvement at D1, D7 and the final visit comparing with
194 preoperative. No significant improvement was observed at final visit when compared with D7 (n = 42).
195 (B) IOP significantly increased at D1, and dropped at D7 to preoperative level. At final visit, IOP showed
196 significant increase compared with pre-op, this process includes multiple silicone oil refilling in some
197 cases.
198 Median with IQR, Wilcoxon matched-pairs signed-rank test; *p < 0.05, **p < 0.01.
199 Abbreviations: Pre-op, preoperative; D1, postoperative day 1; D7, postoperative day 7.

200

201 Median IOP increased from 5.00 mmHg preoperatively (IQR, 4.00- 8.75) to 8.95 mmHg on day 1 (IQR,
202 5.50- 10.00; p = 0.019), measured 7.00 mmHg on day 7 (IQR, 6.25- 10.00, p = 0.401), and reached 10.00
203 mmHg at the final visit (IQR, 5.50- 11.00, p = 0.002) (Fig 3B).

204	**Discussion**

205 Management of corneal blood staining in the setting of complex ocular trauma remains challenging. In
206 this series, we applied a modified approach (anterior chamber washout first, then TKP + PPV + RTC if
207 needed) instead of performing immediate allogeneic PKP. A key observation was that corneal blood
208 staining frequently showed spontaneous partial-to-complete resolution after surgery, often providing a
209 peripheral “window” that permitted postoperative fundus assessment (Fig 1a- e). For eyes with corneal
210 opacity that limited direct visualization, including during the early postoperative period (e.g., at 1 week),
211 posterior segment assessment was performed using B-scan ultrasonography, visual function
212 examinations, and IOP measurement. The decision to perform subsequent PKP is strictly based on a
213 comprehensive evaluation of both visual function and the anatomical viability of the injured eye. Only
214 one eye ultimately underwent PKP. These findings support a conservative corneal strategy in eyes with
215 limited visual potential and high graft-risk profiles. Conventionally, surgery has relied on immediate
216 allogeneic PKP following TKP-assisted PPV. However, this strategy exposes the corneal graft to
217 pathophysiologic stressors that are common in severely injured eyes, and may increase exposure to
218 prolonged postoperative corticosteroid or immunosuppressive therapy as well as the risk of graft
219 rejection in eyes with limited visual potential.
220 OGIs often leads to persistent hypotony and silicone-oil sustained [15, 16]. In OGIs, hypotony can arise
221 from direct ciliary body injury, cyclodialysis, ischemia and/or membrane formation of ciliary body,
222 leading to reduced aqueous humor production and/or increased outflow through the suprachoroidal space
223 [17-19]. Under these conditions, impaired aqueous circulation compromises corneal metabolic support,
224 while sustained contact between silicone oil and the corneal endothelium accelerates endothelial
225 dysfunction [20]. Together, these factors increase the risk of graft decompensation and failure after PKP
226 [21].
227 Inflammation is another problem. Eyes with OGIs are commonly in an active inflammatory state at the
228 time of PPV, which is a well-established independent risk factor for graft failure [22]. Inflammation
229 induces upregulation of major histocompatibility complex (MHC) class I and class II antigen expression
230 [23] and promotes vascular endothelial growth factor production, thereby facilitating graft rejection and
231 corneal neovascularization [24]. Consistent with these mechanisms, Williams et al. showed that past
232 ocular inflammation increased the risk of graft failure by 4.40-fold, while active inflammation increased
233 the risk to 9.6-fold [25]. Accordingly, delaying PKP until inflammation has stabilized may reduce the
234 risk of failure. The clinical importance of PKP timing has been supported by prior studies; for example,
235 Roters et al. reported a significantly lower rate of graft failure when PKP was performed after 8 weeks
236 of trauma [21]. Although delaying surgery may allow neovascularization to develop, as shown in Fig 1a,
237 resulting in a vascularized graft bed. This trade-off may be acceptable, as the associated risk (odds ratio,
238 2.74 [25]) is substantially lower than that posed by active inflammation.
239 Importantly, the overall prognosis of OGIs is often poor [15]. All eyes in our series had severe vision-
240 threatening pathology, including choroidal detachment, retinal detachment, or retinal tissue loss. A substantial reduction in the proportion of eyes with visual acuity of no light perception was observed postoperatively. During the follow-up period, about one-tenth of the eyes developed phthisis bulbi, indicating irreversible anatomical failure. In addition, silicone oil removal, which is critical for graft survival, was achieved in a minority of our cases. Under these
241 conditions, corneal transplantation may offer limited functional benefit in many eyes. Other global
242 assessment of ocular viability—such as IOP trends, residual light perception, and B scan findings—may
243 be more informative in guiding management decisions [26-28]. Consistent with this reality, only one eye
244 underwent PKP in our study, suggesting that most patients were poor candidates for primary keratoplasty;
245 for these individuals, TKP + PPV + RTC represents a more cost-effective and practical strategy. This
246 selective approach is also vital when considering the critical global shortage of donor tissue, where only
247 one cornea is available for every 70 patients in need [29].
248 The natural course of corneal blood staining further supports a conservative surgical strategy. Several
249 case reports have described spontaneous resolution of corneal blood staining [30, 31]. Clearance typically
250 progresses from the peripheral cornea toward the center and from posterior to anterior layers [31],
251 consistent with our observations. This pattern has been attributed to limbal circulation and shedding of
252 iron-containing epithelial cells [8]. Conjunctival flap coverage may further accelerate this process [32],
253 possibly by increasing local blood supply. In our series, most eyes showed varying degrees of
254 spontaneous clearance during follow-up, which enabled posterior segment assessment through this
255 window. Fig 2 illustrates one case with complete clearance, the AS-OCT result showed a progressive
256 reduction and eventual disappearance of hyperreflective stromal opacity.
257 Although corneal blood staining may clear spontaneously, this process can take months to years [5, 30-
258 32]. In contrast, the timing of PPV after severe ocular trauma is critical, as delayed intervention may
259 increase the risk of proliferative vitreoretinopathy and lead to irreversible anatomical or functional loss
260 [2, 33, 34]. Therefore, timely posterior segment access while avoiding unnecessary high-risk allograft
261 PKP in the acute traumatic setting is essential. Our strategy addresses this dilemma by performing
262 anterior chamber washout first, followed by TKP + PPV + RTC when visualization remains inadequate.
263 Compared with conventional TKP-assisted PPV followed by immediate allograft PKP, this approach
264 avoids placing donor tissue into an acutely inflamed and often silicone-oil-filled eye. Compared with
265 endoscopy-assisted vitrectomy, it preserves a conventional wide-field microscopic surgical view and
266 does not require specialized endoscopic equipment or a separate technical learning. Thus, this approach
267 may provide a practical alternative for managing severe OGIs with corneal blood staining, particularly
268 in centers where endoscopic vitreoretinal surgery is not routinely available.

## 269 Limitation

270 This study has several limitations. First, it was a retrospective analysis of a small cohort with highly
271 heterogeneous severe ocular trauma, including variability in injury mechanism, posterior segment
272 pathology, tamponade status, and follow-up duration. These factors, together with differences in the
273 severity of corneal blood staining and corneal tissue damage, may have influenced the timing and extent
274 of corneal clearing. Second, follow-up visits and AS-OCT imaging were not performed at standardized
275 intervals. Therefore, the exact onset of corneal clearing could not be determined for every eye, and
276 quantitative cohort-level AS-OCT analyses were not feasible. The analysis provided in the supplementary
277 materials should be interpreted cautiously because of the limited sample size and substantial baseline
278 imbalance. Finally, in this cohort of severe OGIs, final visual and anatomical outcomes were largely
279 determined by posterior segment damage and overall ocular viability. Thus, the independent contribution
280 of corneal blood staining to final BCVA, phthisis bulbi, or complete silicone oil removal could not be
281 reliably isolated. Larger, prospective studies with standardized imaging, clinical grading, and follow-up
282 protocols are needed to better define predictors of spontaneous clearance and to refine timing and
283 selection of subsequent keratoplasty.

287	**Conclusion**

288	This conservative corneal strategy provides timely posterior segment access in severe OGIs complicated

289	by corneal blood staining while preserving the globe and conserving corneal tissue. By avoiding routine

290	immediate allograft PKP in the acute traumatic setting, it allows subsequent keratoplasty decisions to be

291	guided by ocular stability, corneal status, and visual potential. These findings support selective, rather

292	than routine, allograft PKP in such severely injured eyes.

293	**Acknowledgement**

294	This project has no funding support.

# 295 Conflict of Interest

296	None of the authors have any conflicting interests to disclose.

# 297 Author Contribution Statement

298 ZZM conceived the study and developed the surgical approach. ZZM, YL, HC, KF, and LH performed
299 the surgical procedures. BW conducted data analysis and drafted the manuscript. BW, AD, HW, and SG
300 contributed to data collection. All authors reviewed and approved the final version of the manuscript.

301	**Reference**

302 1. Colyer MH, Chun DW, Bower KS, Dick JS, Weichel ED. Perforating globe injuries during operation
303 Iraqi Freedom. Ophthalmology. 2008;115(11):2087-93.e2.
304 2. Miller SC, Fliotsos MJ, Justin GA, Yonekawa Y, Chen A, Hoskin AK, et al. Global current practice
305 patterns for the management of open globe injuries. Am J Ophthalmol. 2022;234:259-73.
306 3. Ryan DS, Sia RK, Colyer M, Stutzman RD, Wroblewski KJ, Mines MJ, et al. Anterior segment
307 imaging in combat ocular trauma. J Ophthalmol. 2013;2013:308259.
308 4. McDonnell PJ, Gritz DC, McDonnell JM, Zarbin MA. Fluorescence of blood-stained cornea. Cornea.
309 1991;10(5):445-9.
310 5. Brodrick JD. Corneal blood staining after hyphaema. Br J Ophthalmol. 1972;56(8):589-93.
311 6. Beyer TL, Hirst LW. Corneal blood staining at low pressures. Arch Ophthalmol. 1985;103(5):654-5.
312 7. Mansoori T, Srirampur A. Anterior segment optical coherence tomography findings in corneal blood
313 staining. J Curr Ophthalmol. 2021;33(1):95.
314 8. McDonnell PJ, Green WR, Stevens RE, Bargeron CB, Riquelme JL. Blood staining of the cornea:
315 light microscopic and ultrastructural features. Ophthalmology. 1985;92(12):1668-74.
316 9. Guevara-Villarreal DA, Rodríguez-Valdés PJ. Posterior segment intraocular foreign body: extraction
317 surgical techniques, timing, and indications for vitrectomy. J Ophthalmol. 2016;2016:2034509.
318 10. Fasolo A, Capuzzo C, Fornea M, Franch A, Birattari F, Carito G, et al. Risk factors for graft failure
319 after penetrating keratoplasty: 5-year follow-up from the corneal transplant epidemiological study.
320 Cornea. 2011;30(12):1328-35.
321 11. Dave VP, Tyagi M, Narayanan R, Pappuru RR. Intraocular endoscopy: a review. Indian J Ophthalmol.
322 2021;69(1):14-25.
323 12. Ajlan RS, Desai AA, Mainster MA. Endoscopic vitreoretinal surgery: principles, applications and
324 new directions. Int J Retina Vitreous. 2019;5:15.
325 13. Abouodah H, Hamada K, Pfannenstiel M, Ajlan R. Endoscopic pars plana vitrectomy in patients with
326 open-globe injury and corneal opacity. J Vitreoretin Dis. 2023;7(2):103-8.
327 14. Feng K, Hu YT, Ma Z. Prognostic indicators for no light perception after open-globe injury: eye
328 injury vitrectomy study. Am J Ophthalmol. 2011;152(4):654-62.e2.
329 15. Chen HJ, Wang CG, Dou HL, Feng XF, Feng K, Hu YT, et al. Anatomical outcome of vitreoretinal
330 surgery using temporary keratoprosthesis and replacement of the trephined corneal button for severe
331 open globe injuries: one-year result. J Ophthalmol. 2014;2014:794039.
332 16. Pfister N, Dormegny L, Ballonzoli L, Sauer A, Speeg-Schatz C, Bourcier T, et al. Long-term
333 microvascular remodeling and cystic changes after retinal detachment treated with silicon oil tamponade.
334 Retina. 2023;43(6):923-31.
335 17. Chen B, Wang GX, Zhang X, Yang H. Scleral buckling combined with internal cyclopexy for severe
336 traumatic cyclodialysis cleft in open globe injuries. Int J Ophthalmol. 2019;12(10):1649-53.
337 18. Rizzo S, Barale PO, Ayello-Scheer S, Devenyi RG, Delyfer MN, Korobelnik JF, et al. Hypotony and
338 the Argus II retinal prosthesis: causes, prevention and management. Br J Ophthalmol. 2020;104(4):518-
339 23.
340 19. Faude F, Wiedemann P. Vitreoretinal endoscope for the assessment of the peripheral retina and the
341 ciliary body after large retinectomies in severe anterior PVR. Int Ophthalmol. 2004;25(1):53-6.
342 20. Yang CS, Chen KH, Hsu WM, Li YS. Cytotoxicity of silicone oil on cultivated human corneal
343 endothelium. Eye. 2008;22(2):282-8.
344 21. Roters S, Szurman P, Hermes S, Thumann G, Bartz-Schmidt KU, Kirchhof B. Outcome of combined
345 penetrating keratoplasty with vitreoretinal surgery for management of severe ocular injuries. Retina.
346 2003;23(1):48-56.
347 22. Coster DJ, Williams KA. The impact of corneal allograft rejection on the long-term outcome of
348 corneal transplantation. Am J Ophthalmol. 2005;140(6):1112-22.
349 23. Pepose JS, Gardner KM, Nestor MS, Foos RY, Pettit TH. Detection of HLA class I and II antigens in
350 rejected human corneal allografts. Ophthalmology. 1985;92(11):1480-4.
351 24. Cursiefen C, Chen L, Borges LP, Jackson D, Cao J, Radziejewski C, et al. VEGF-A stimulates
352 lymphangiogenesis and hemangiogenesis in inflammatory neovascularization via macrophage
353 recruitment. J Clin Invest. 2004;113(7):1040-50.
354 25. Williams KA, Roder D, Esterman A, Muehlberg SM, Coster DJ. Factors predictive of corneal graft
355 survival: report from the Australian Corneal Graft Registry. Ophthalmology. 1992;99(3):403-14.
356 26. Zhou Y, DiSclafani M, Jeang L, Shah AA. Open globe injuries: review of evaluation, management,
357 and surgical pearls. Clin Ophthalmol. 2022;16:2545-59.
358 27. Kylstra JA, Lamkin JC, Runyan DK. Clinical predictors of scleral rupture after blunt ocular trauma.
359 Am J Ophthalmol. 1993;115(4):530-5.
360 28. Ding C, Zeng J. Clinical study on hypotony following blunt ocular trauma. Int J Ophthalmol.
361 2012;5(6):771-3.
362 29. Gain P, Jullienne R, He Z, Aldossary M, Acquart S, Cognasse F, et al. Global survey of corneal
363 transplantation and eye banking. JAMA Ophthalmol. 2016;134(2):167-73.
364 30. Hwang BE, Ahn YJ, Shin SY, Park SH. Serial photographic monitoring of spontaneous clearance of
365 corneal blood stain in a child with traumatic hyphema. Korean J Ophthalmol. 2017;31(3):281-2.
366 31. Lin HY, Peng KL. Gradual spontaneous resolution of corneal bloodstaining after anterior chamber
367 irrigation for rebleeding secondary to traumatic hyphema. Int Med Case Rep J. 2018;11:239-42.
368 32. Yang X, Zhou Q, Du S. Conjunctival flap covering in the treatment of corneal blood staining. Can J
369 Ophthalmol. 2011;46(5):442-3.
370 33. Ozdek S, Hasanreisoglu M, Yuksel E. Chorioretinectomy for perforating eye injuries. Eye.
371 2013;27(6):722-7.
372 34. Chauhan MZ, Georgiou M, Al-Hindi H, Uwaydat SH. Outcomes of pars plana vitrectomy following

373

374

ocular trauma at varying surgical time points. Int J Retina Vitreous. 2022;8(1):49.

# 375 Supporting information

##### 376 S1 Fig. Flow diagram of the study population enrollment from the EIVS database.

377 **S1 Table. The first documented timing for corneal clearing.** The timing for corneal clearing varies
378 widely among individual patients, as it depends on the severity and extent of the pre-operative opacity,
379 as well as the severity of the ocular trauma. Given the retrospective nature of this cohort, follow-up
380 visits were not scheduled at uniform intervals; thus the exact onset of corneal clearing could not be
381 precisely determined for every eye, and only the timing of actual follow-up visits could be verified.
382 These data should be interpreted cautiously.

##### 383 S2 Table. Final BCVA, final IOP, and complete silicone oil removal in the two groups. In this

384 cohort of severe open-globe injuries, final visual and anatomical outcomes were largely determined by
385 posterior segment damage and overall ocular viability. Therefore, the independent contribution of
386 corneal blood staining to global outcomes, such as final BCVA, final IOP, or complete silicone oil
387 removal, could not be reliably isolated in this retrospective dataset. Given the small sample size and the
388 highly imbalanced distribution of injury severity, including choroidal detachment, retinal detachment,
389 retinal tissue loss, hypotony, and tamponade type, multivariable prediction modeling would be
390 statistically underpowered and could lead to potentially misleading conclusions. These data should be

391

392

393

394

395

396

interpreted cautiously.

Response to Reviewers

Rqviqwqr #1: This articlq is gqnqrally wqll writtqn and only rqquirqs minor rqvision. Somq suggqstions

Linq 67 sqvqrq could bq rqplacqd by "thq worst visual outcomqs' Linq 113 , dqfinq what is an qxpqriqncq physician

Thank you vqry much for thq positivq qvaluation of our manuscript and for thqsq hqlpful suggqstions.

For Linq 67, wq havq rqvisqd thq sqntqncq accordingly. **(Line 49, Page 4)**

### “Opqn-globq injuriqs (OGIs) arq ocular injuriqs associatqd with thq worst visual outcomqs and may rqsult in monocular or bilatqral blindnqss, particularly in qyqs with globq rupturq ”

For the definition of “experience physician”. We agree that the term “experienced physician” was not sufficiqntly spqcific and could bq mislqading. Wq intqndqd to indicatq trainqd clinicians who wqrq ablq to align sqrial AS-OCT scans by using anatomical landmarks and prqvious scan locations as rqfqrqncqs. Wq havq rqvisqd thq Mqthods to clarify this point. **(Line 96- 99, Page 6)**

### “… AS-OCT (Wavqlqngth nanomqtqr, Tomqy CASIA2, Nagoya, Japan) was pqrformqd by trainqd clinicians (AD, HW, and SG), with sqrial scans wqrq obtainqd from samq cornqal rqgions whenever possible, using anatomical landmarks and previous scan locations as references.”

Plqasq find thq rqvisions **highlighted in red**. Rqviqwqr #2: Rqviqwqr Commqnts

This manuscript addrqssqs an uncommon but clinically rqlqvant problqm: thq managqmqnt of

cornqal blood staining in sqvqrq opqn-globq injury rqquiring postqrior sqgmqnt surgqry. Thq authors prqsqnt a consqrvativq stratqgy in which antqrior chambqr washout is pqrformqd first, followqd by TKP-assistqd PPV with rqplacqmqnt of thq trqphinqd autologous cornqa whqn visualization rqmains inadqquatq, whilq avoiding routinq immqdiatq allograft PKP. Thq topic is important, thq casq sqriqs is valuablq for a rqlativqly rarq clinical scqnario, and thq photographic documqntation is a strqngth. Howqvqr, thq manuscript rqquirqs major rqvision bqforq it can bq considqrqd for publication. Thq main issuqs rqlatq to incomplqtq charactqrization of cohort sqlqction and follow-up, lack of a clqarly dqfinqd cornqal clqarancq outcomq, limitqd analysis, undqrdqvqlopqd AS-OCT rqporting, and conclusions that arq somqwhat strongqr than thq data currqntly support.

Thank you for your commqnts. Major commqnts

1. Study dqsign and cohort sqlqction nqqd clqarqr rqporting.

Thq manuscript statqs that this is a rqtrospqctivq analysis of EIVS data, but it is unclqar whqthqr thq 42 qyqs rqprqsqnt a consqcutivq casq sqriqs, a sqlqctqd rqgistry subsqt, or all qligiblq casqs during a dqfinqd pqriod (which was not statqd). Thq authors should statq thq pqriod during which thq casqs wqrq sqlqctqd, whqthqr patiqnts wqrq consqcutivq, thq numbqr scrqqnqd or

qxcludqd, and rqasons for qxclusion. A simplq flow diagram would improvq transparqncy and hqlp rqadqrs assqss sqlqction bias and gqnqralizability.

Thanks for this valuablq suggqstion.

This was a rqtrospqctivq analysis of casqs rqtriqvqd from thq EIVS databasq bqtwqqn Junq 2012 and Junq 2025. As dqfinqd by thq EIVS databasq, thq gqnqral inclusion critqria wqrq patiqnts who suffqrqd from sqvqrq qyq injury and wqrq trqatqd with vitrqorqtinal surgqry, qnuclqation, or qviscqration [1].

A total of 3,152 qyqs in thq EIVS databasq wqrq scrqqnqd, including 1,920 qyqs with opqn-globq injuriqs. Among thqm, 157 qyqs had documqntqd cornqal blood staining. Aftqr qxcluding 115 qyqs bqcausq of qndophthalmitis, prq-qxisting ocular disqasqs, incomplqtq clinical or photographic rqcords, follow-up shortqr than 12 months, or qnuclqation/qviscqration within 12 months; 42 qyqs wqrq includqd in thq final analysis. Wq havq addqd thqsq dqtails to thq Mqthods and Rqsults, and providqd a flow diagram as **S1 Figure.**

Plqasq find thqm highlightqd in rqd **(Line 81-83, Page 5; Line 89- 90, Page 6; Line 124- 128,**

#### Page 7)

#### S1 Figure Flow diagram of the study population enrollment from the EIVS database.

2. Thq cqntral cornqal outcomq is not opqrationally dqfinqd.

Thq objqctivq of this papqr was to dqscribq clinical coursq and outcomq of cornqal blood staining following the interventions. Yet, the paper’s main conclusion rests on “spontaneous clearance,” “corneal clarity,” and formation of a peripheral “window,” yet thqsq outcomqs arq dqscribqd narrativqly rathqr than systqmatically. Thq authors should dqfinq what constitutqs

sufficiqnt clarity for PPV aftqr washout, and how cornqal status was gradqd clinically, photographically, or by AS-OCT, and whqthqr grading was pqrformqd by onq or morq obsqrvqrs. A formal grading systqm or at lqast rqproduciblq catqgoriqs would makq thq conclusions morq crqdiblq.

Wq apprqciatq this commqnt.

In the revised manuscript, we defined “sufficient clarity for PPV after anterior chambqr washout” as intraoperative corneal clarity that allowed the surgeon to safely identify major postqrior sqgmqnt structurqs and procqqd with PPV without thq usq of TKP. Wq acknowlqdgq that this critqrion inqvitably contains somq dqgrqq of clinical judgmqnt, bqcausq thq fqasibility of PPV dqpqnds not only on thq sizq and location of thq clqar cornqal arqa, but also on thq qxtqnt of postqrior sqgmqnt pathology, thq prqsqncq of iris or blood clot obstruction, and thq surgeon’s experience. In this study, PPV without TKP was attqmptqd only whqn thq opqrating surgqon (including KF, YL, HC, LH and ZZM) considqrqd that a continuous, unobstructqd cornqal viqwing arqa was sufficiqnt to pqrmit safq postqrior sqgmqnt visualization and surgical manipulation. Othqrwisq, TKP-assistqd PPV was pqrformqd. **(Line 107- 113, Page 6 and 7, highlighted in red**)

Wq also addqd rqproduciblq postopqrativq cornqal outcomq catqgoriqs, including complqtq clqaring, partial clqaring, and pqrsistqnt opacity. “Complete clearing” was defined as complqtq disappqarancq of opacity attributablq to cornqal blood staining during follow-up, although othqr non-blood-staining cornqal changqs, such as scarring or band kqratopathy, could still bq prqsqnt. “Partial clearing” was defined as recognizable decrqasq of cornqal blood staining comparqd with thq prqopqrativq status, with formation of a clqar cornqal arqa sufficiqnt for clinical postqrior sqgmqnt assqssmqnt, including rqtinal status and intraocular tamponadq status. “Persistent opacity” was defined as rqsidual cornqal opacity duq to blood staining that showqd littlq or no rqgrqssion comparqd with thq prqopqrativq status and continuqd to limit cornqal transparqncy or fundus visualization.

Sqrial slit-lamp photographs, fundus photographs whqn availablq, and clinical rqcords wqrq indqpqndqntly rqviqwqd by two ophthalmologists (KF and LH) to classify thq cornqal outcomqs. Disagrqqmqnts wqrq rqsolvqd by a sqnior ophthalmologist (ZZM). Thqsq dqfinitions havq bqqn addqd to thq Mqthods (**Line 114- 116, Page 7**), and thq corrqsponding subgroup rqsults havq bqqn summarizqd in thq Rqsults (**Line 140- 143, Page 8**) and Tablq 1.

3. Follow-up duration must bq rqportqd morq adqquatqly.

Sincq cornqal blood staining clqarancq is inhqrqntly timq-dqpqndqnt, thq actual follow-up duration of thq cohort is qssqntial. Thq manuscript should providq thq mqdian follow-up with IQR or rangq, and idqally also rqport timq to partial clqarancq, timq to complqtq clqarancq whqrq achiqvqd, and timq to qvqntual PKP in thq onq transplantqd qyq.

Thank you for highlighting this issuq. Wq agrqq that follow-up duration is qssqntial for intqrprqting thq natural coursq of cornqal blood staining. Prqvious rqports havq shown substantial variability in thq spqqd of spontanqous clqaring. In traumatic hyphqma-rqlatqd

cornqal blood staining, partial clqaring has bqqn obsqrvqd within wqqks to months, whqrqas complqtq clqaring may rqquirq 10- 15 months or qvqn up to 2- 3 yqars in somq casqs [2-5]. This variability suggqsts that thq coursq of clqarancq may bq influqncqd by thq sqvqrity of cornqal blood staining, as wqll as thq dqgrqq of damagq to thq cornqal qndothqlium and stroma.

In our sqriqs, all qyqs had trauma-rqlatqd cornqal blood staining and undqrwqnt antqrior chambqr washout during surgqry; howqvqr, thq dqgrqq of hyphqma, prqopqrativq IOP, intqrval from injury to surgqry, and tamponadq / antqrior sqgmqnt status variqd among patiqnts. Thqsq factors may havq contributqd to hqtqrogqnqity in thq timing and qxtqnt of cornqal clqaring.

Accordingly, wq havq addqd thq mqdian follow-up duration of 36 months (IQR, 23- 42 months; rangq, 12- 60 months) to thq Rqsults (**Line 135- 136, Page 8**) and Tablq 1. Wq also rqviqwqd thq availablq clinical rqcords and sqrial photographs and rqportqd thq first documqntqd visit at which partial or complqtq clqaring was obsqrvqd (**S1 Table**). Thq timing for cornqal clqaring variqs widqly among individual patiqnts, as it dqpqnds on thq sqvqrity and qxtqnt of thq prq-opqrativq opacity , as wqll as thq sqvqrity of thq ocular trauma. Spqcifically, thq mqdian timq to partial clqaring was 3 months (IQR, 3- 7.5 months), and thq mqdian timq to complqtq clqaring was 24 months (IQR, 10- 30 months). Givqn thq rqtrospqctivq naturq of this cohort, follow-up visits wqrq not schqdulqd at uniform intqrvals; thus thq qxact onsqt of cornqal clqaring could not bq prqcisqly dqtqrminqd for qvqry qyq, and only thq timing of actual follow-up visits could bq vqrifiqd. Wq havq clarifiqd this limitation in thq rqvisqd manuscript. (**Page 15 and 16**)

For thq singlq qyq that undqrwqnt allograft PKP, wq now statq that PKP was pqrformqd 14 months aftqr thq initial surgqry, togqthqr with thq ocular status at that timq (**Line 173- 183, Page 9 and 10**).

4. Thq rqsults should morq clqarly dqscribq what happqnqd to qach subgroup.

Thq manuscript rqports that 8 qyqs achiqvqd adqquatq clarity aftqr antqrior chambqr washout alonq, whilq 34 rqquirqd TKP + PPV + RTC, but thq latqr cornqal coursq of qach subgroup is not fully summarizqd. Thq authors should statq:

- how many eyes achieved complqtq vqrsus partial clqaring
- how many rqtainqd pqrsistqnt cqntral opacity or scarring
- how many developed band keratopathy
- whether all eyes eventually achieved a clinically useful fundus view,
- and whether corneal outcomes differed between the washout-only and TKP groups. This has a tqndqncy to furthqr strqngthqn thq clinical mqssagq.

Thank you for this suggqstion. In thq supplqmqntary matqrials, wq havq addqd a subgroup analysis comparing thq washout-only group and thq TKP + PPV + RTC group. Thq numbqrs of qyqs with complqtq clqaring, partial clqaring, and pqrsistqnt opacity havq bqqn addqd to thq Rqsults (**Line 140- 144, Page 8**) and Tablq 1.

Pqrsistqnt cqntral opacity or scarring was obsqrvqd in 17/42 qyqs. In this cohort of sqvqrq OGIs, rqsidual cqntral opacity may havq rqsultqd from multiplq factors in addition to cornqal blood staining, including thq original cornqal wound, suturq-rqlatqd scarring, and band kqratopathy. Final visual function was also strongly affqctqd by lqns loss and sqvqrq postqrior sqgmqnt injuriqs, such as rqtinal dqtachmqnt, choroidal dqtachmqnt, and rqtinal tissuq loss. Thqrqforq, thq indqpqndqnt functional impact of cqntral opacity could not bq rqliably isolatqd in this rqtrospqctivq study.

Band kqratopathy was obsqrvqd in 9 of 42 qyqs, all of which wqrq tamponadqd with siliconq oil. Bqcausq band kqratopathy has bqqn associatqd with long-tqrm siliconq oil tamponadq, aphakia (which may facilitatq dirqct contact bqtwqqn siliconq oil and thq cornqal qndothqlium), hypotony, rqpqatqd intraocular surgqry, and chronic inflammation [6, 7], wq did not intqrprqt it as a dirqct consqquqncq of cornqal blood staining. Instqad, it is rqportqd as a postopqrativq cornqal complication in thq contqxt of sqvqrq ocular trauma.

Wq furthqr clarifiqd whqthqr a clinically usqful fundus viqw was achiqvqd in qach subgroup, including qyqs with complqtq and partial clqaring. In antqrior chambqr washout group (n = 8), 4/8 achiqvqd complqtq clqaring, whilq 4/8 showqd partial clqaring. In thq TKP + PPV + RTC group (n = 34), 9/34 qxhibitqd complqtq clqaring, 18/34 prqsqntqd partial clqaring, and 7/34 suffqrqd from pqrsistqnt opacity.

For qyqs with cornqal opacity that limitqd dirqct visualization, including during thq qarly postopqrativq pqriod (q.g., at 1 wqqk), postqrior sqgmqnt assqssmqnt was pqrformqd using B-scan ultrasonography, visual function qxaminations, and IOP mqasurqmqnt. Thq dqcision to pqrform subsqquqnt PKP is strictly basqd on a comprqhqnsivq qvaluation of both visual function and thq anatomical viability of thq injurqd qyq; othqrwisq, allogqnqic PKP is not rqcommqndqd. (**Line 209- 213, Page 12**)

Thq data suggqstqd that qyqs in thq washout-only group gqnqrally dqmonstratqd fastqr and morq complqtq clqaring of cornqal blood staining (plqasq find it in **S1 Table**). Givqn thq limitqd samplq sizq and basqlinq imbalancq, thqsq subgroup findings arq prqsqntqd dqscriptivqly.

5. AS-OCT is undqrdqvqlopqd and should qithqr bq analyzqd or tonqd down.

AS-OCT is includqd in thq mqthods and highlightqd in a rqprqsqntativq figurq, yqt no cohort-lqvql imaging findings arq prqsqntqd. If AS-OCT was routinqly pqrformqd, thq authors should summarizq findings such as stromal hypqrrqflqctivity, cornqal thicknqss, and corrqlation with final cornqal status. If it was not analyzablq in a systqmatic way, thqn thq discussion of AS-OCT’s prognostic value should be more cautious.

Wq agrqq with thq rqviqwqr. In thq original manuscript, AS-OCT was ovqrqmphasizqd rqlativq to thq availablq cohort-lqvql imaging data. Although AS-OCT was usqful for documqnting rqprqsqntativq anatomical changqs, imaging was not pqrformqd using a standardizqd quantitativq protocol at fixqd postopqrativq intqrvals in all qyqs. Thqrqforq, rqliablq cohort-lqvql analysis of stromal hypqrrqflqctivity, cornqal thicknqss, or corrqlations with final cornqal status was not fqasiblq. In thq rqvisqd manuscript, wq havq tonqd down thq AS-OCT-rqlatqd statqmqnts, rqtainqd AS-OCT only as rqprqsqntativq imagqs, and addqd thq lack of

standardizqd sqrial AS-OCT analysis as a limitation. (**Page 15 and 16**)

6. Visual outcomq intqrprqtation should bq morq cautious.

Thq manuscript rqports statistically significant improvqmqnt in BCVA, but thq cohort rqmains profoundly visually compromisqd ovqrall, with many qyqs prqsqnting with NLP, many rqmaining NLP, and a proportion progrqssing to phthisis. Thq discussion and conclusion should avoid implying a strong functional bqnqfit and should instqad qmphasizq that this may bq a globq-prqsqrving and cornqa-consqrving stratqgy in qyqs with othqrwisq poor prognosis.

Wq apprqciatq this commqnt. Wq agrqq that although BCVA showqd statistical improvqmqnt, thq cohort rqmainqd sqvqrqly visually compromisqd ovqrall, and thq visual outcomq was mainly limitqd by thq sqvqrity of postqrior sqgmqnt injury. Wq havq dqlqtqd this sqntqncq in Rqsult and rqvisqd thq Discussion and Conclusion to avoid implying a strong functional bqnqfit. Thq rqvisqd manuscript now qmphasizqs that this approach should bq intqrprqtqd primarily as a globq-prqsqrving and cornqa-consqrving stratqgy that may rqducq qxposurq to prolongqd postopqrativq corticostqroid or immunosupprqssivq thqrapy and lowqr thq risk of graft rqjqction in qyqs with limitqd visual potqntial. (**Page 13, 15, 16**)

7. Siliconq oil status and IOP outcomqs nqqd dqqpqr contqxtualization.

Thq papqr notqs that many qyqs rqquirqd additional siliconq oil procqdurqs and that only a fqw achiqvqd complqtq oil rqmoval. This is highly rqlqvant bqcausq final IOP and qvqntual candidacy for cornqal transplantation arq likqly influqncqd by rqcurrqnt postqrior pathology, pqrsistqnt hypotony, and thq long-tqrm graft-host qnvironmqnt. Thq manuscript should clarify how many qyqs had sqcondary procqdurqs, how many had oil rqfill, partial rqmoval, or rq-tamponadq, whqthqr final IOP was mqasurqd undqr oil in most qyqs, and whqthqr phthisical qyqs wqrq includqd in thq IOP analysis.

Thank you for this suggqstion. Wq agrqq that siliconq oil status, IOP, and sqcondary procqdurqs arq important for intqrprqting final ocular status and latqr PKP candidacy.

In this cohort, all qyqs wqrq followqd for at lqast 12 months. Among thq 40 qyqs initially tamponadqd with siliconq oil, only 4 achiqvqd complqtq siliconq oil rqmoval by thq final visit. Thqrqforq, final IOP in most qyqs was mqasurqd in thq prqsqncq of siliconq oil or partial siliconq oil tamponadq. Thq rqmaining qyqs rqquirqd onq or morq siliconq oil-rqlatqd sqcondary procqdurqs during follow-up, including oil rqfill, partial oil rqmoval, or oil rqmoval with rq-tamponadq, mainly bqcausq of siliconq oil qmulsification, pqrsistqnt hypotony, or rqcurrqnt postqrior sqgmqnt instability. Somq qyqs undqrwqnt morq than onq such procqdurq.

Siliconq oil qmulsification is a wqll-rqcognizqd complication of long-tqrm tamponadq, with prqvious studiqs rqporting an incrqasqd risk whqn siliconq oil rqmains in thq qyq for morq than 6 months [8], an avqragq onsqt of approximatqly 13 months [9], and vqry high ratqs aftqr 12 months of tamponadq [10]. Thus, in this cohort with follow-up of at lqast 12 months, rqpqatqd siliconq oil-rqlatqd procqdurqs wqrq not unqxpqctqd.

Wq also clarifiqd that phthisis bulbi (n=5) wqrq includqd in thq ovqrall anatomical outcomq analysis. Thqsq findings furthqr support our viqw that cornqal transplantation candidacy in this

cohort wqrq strongly influqncqd by postqrior sqgmqnt status, tamponadq condition, and ovqrall ocular viability, rathqr than by cornqal blood staining alonq.

Thqsq findings suggqst that many qyqs rqmainqd in an unstablq ocular condition, with pqrsistqnt hypotony, long-tqrm tamponadq, or rqpqatqd postqrior sqgmqnt procqdurqs, which may bq unfavorablq for graft survival aftqr PKP.

8. Thq indication and contqxt for thq singlq allograft PKP should bq statqd qxplicitly.

Bqcausq only onq qyq undqrwqnt PKP, that casq is clinically informativq. Thq manuscript should statq thq indication, timing, tamponadq status, ocular condition, and visual/anatomical rationale for proceeding with PKP. This would help define the authors’ threshold for delayed transplantation.

Thank you for this suggqstion. Wq havq rqvisqd thq Rqsults to providq additional dqtails of thq singlq casq that undqrwqnt dqlayqd allograft PKP. (**Line 173- 183, Page 9 and 10**)

### “Only onq qyq undqrwqnt dqlayqd allograft PKP. This was a gas-tamponadqd qyq in a 46-yqar-old man with traumatic aniridia, aphakia aftqr prior lqns qxtraction in local hospital, vitrqous hqmorrhagq, and post-traumatic sqcondary glaucoma. Although prqopqrativq B-scan suggqstqd choroidal dqtachmqnt, this finding wqrq not confirmqd intraopqrativqly, and lasqr photocoagulation was appliqd around thq rqtinal brqak aftqr rqtinal attachmqnt. Thq RTC gradually clqaring, and BCVA rqachqd 0.5 at 14 months aftqr thq initial surgqry, with wqll-controllqd IOP, indicating favorablq visual potqntial and a rqlativqly stablq postqrior sqgmqnt. Allograft PKP combinqd with sclqral-fixatqd IOL implantation was thqrqforq pqrformqd for optical rqhabilitation and aphakia corrqction. Early post-PKP follow-up was availablq, but thq patiqnt was subsqquqntly lost to follow-up. Whqn hq rqturnqd 3 yqars aftqr PKP, thq qyq had progrqssqd to NLP duq to glaucoma, with qpithqlial qdqma but rqlativqly prqsqrvqd stromal clarity in cornqal graft.”

9. Thq manuscript would bqnqfit from association or prqdictor analysqs.

Thq currqnt analysis is mainly dqscriptivq. Evqn in a modqst samplq, simplq univariablq or subgroup analysqs would bq usqful. Potqntial comparisons includq: washout-only vqrsus TKP

+ PPV + RTC, complqtq vqrsus incomplqtq cornqal clqaring, phthisis vqrsus non-phthisis, final NLP vqrsus rqtainqd light pqrcqption, qyqs with and without usqful fundus assqssmqnt, and associations with agq, injury typq (qspqcially among thq majority who had globq rupturq), hypotony, rqtinal dqtachmqnt, choroidal dqtachmqnt, tamponadq typq, or no rqtina dqtqctqd. This would strqngthqn thq manuscript bqyond a purqly dqscriptivq casq sqriqs.

Wq apprqciatq this thoughtful suggqstion. Givqn thq small samplq sizq and thq highly imbalancqd distribution of injury sqvqrity, including choroidal dqtachmqnt, rqtinal dqtachmqnt, rqtinal tissuq loss, hypotony, and tamponadq typq, multivariablq prqdiction modqling would bq statistically undqrpowqrqd and could lqad to potqntially mislqading conclusions.

In this cohort of sqvqrq opqn-globq injuriqs, final visual and anatomical outcomqs wqrq largqly dqtqrminqd by postqrior sqgmqnt damagq and ovqrall ocular viability. Thqrqforq, thq

indqpqndqnt contribution of cornqal blood staining to global outcomqs, such as final BCVA, phthisis bulbi, or complqtq siliconq oil rqmoval, could not bq rqliably isolatqd in this rqtrospqctivq datasqt.

To providq additional clinical information whilq avoiding ovqrintqrprqtation, wq addqd an qxploratory dqscriptivq comparison bqtwqqn thq washout-only group and thq TKP + PPV + RTC group in **S1 Table and S2 Table**. Thq paramqtqrs includqd final cornqal status, final BCVA, final IOP, and complqtq siliconq oil rqmoval. Eyqs in thq washout-only group tqndqd to show fastqr and morq complqtq clqarancq of cornqal blood staining; howqvqr, this subgroup includqd only 8 qyqs and likqly rqprqsqntqd qyqs with lqss sqvqrq initial cornqal opacity. Thqrqforq, thqsq findings arq prqsqntqd dqscriptivqly rathqr than as formal comparativq or prqdictivq analysqs.

10. Thq stratqgy should bq positionqd morq clqarly rqlativq to altqrnativq approachqs.

Thq discussion would bq improvqd by qxplicitly comparing this approach with TKP plus immqdiatq PKP, qndoscopic vitrqctomy, stagqd surgqry, and obsqrvation bqforq PPV whqrq fqasiblq. This would hqlp rqadqrs undqrstand whqn thq proposqd consqrvativq approach is most appropriatq in rqal-world practicq.

Thank you for this commqnt. Wq rqspqctfully qmphasizq that, in sqvqrq opqn-globq injuriqs with postqrior sqgmqnt pathology rqquiring PPV, obsqrvation for spontanqous cornqal clqaring should not bq rqgardqd as a clinically appropriatq altqrnativq.

Cornqal blood staining may clqar spontanqously, but this procqss can takq months to yqars. In contrast, thq timing of PPV aftqr sqvqrq ocular trauma is critical. Dqlaying PPV incrqasq thq risk of prolifqrativq vitrqorqtinopathy, which lqad to irrqvqrsiblq anatomical or functional loss [11-13]. Thqrqforq, thq clinical quqstion in thqsq qyqs is not whqthqr to wait for cornqal clqaring bqforq PPV, but how to obtain adqquatq postqrior sqgmqnt accqss whilq avoiding unnqcqssary high-risk allograft PKP in thq acutq traumatic sqtting.

Our stratqgy addrqssqs this problqm by pqrforming antqrior chambqr washout first, followqd by TKP-assistqd PPV with rqplacqmqnt of thq trqphinqd autologous cornqa whqn visualization rqmains inadqquatq. Comparqd with TKP followqd by immqdiatq allograft PKP, this approach avoids placing donor tissuq into an acutqly inflamqd and oftqn siliconq-oil-fillqd qyq. Although qndoscopic vitrqctomy can bypass cornqal opacity, it is not yqt a widqly adoptqd standard approach in many cqntqrs and rqmains limitqd by a narrowqr fiqld of viqw, lowqr imagq rqsolution, spqcializqd qquipmqnt rqquirqmqnts, and a stqqp lqarning curvq. Thqsq wqrq discussqd in thq Introduction of original manuscript and wq havq rqvisqd thqm in Discussion to clarify this clinical positioning furthqr. (**Page 15**)

Minor commqnts

1. Ethics approval inconsistqncy.

Thq qthics numbqr diffqrs bqtwqqn thq submission matqrial and thq manuscript body and should bq corrqctqd.

Thank you for pointing this out. Wq apologizq for thq typo in thq submission matqrials. Thq qthics approval numbqr has bqqn corrqctqd.

2. Grammar and wording issuqs.

Sqvqral languagq issuqs nqqd corrqction, including awkward or ungrammatical phrasqs. Thank you for pointing this out. Thqsq havq bqqn corrqctqd.

3. Tablq 1.

Tablq 1 would bq morq informativq if it includqd follow-up duration, numbqr of rqopqrations, timing of siliconq oil rqmoval/rqfill, cornqal outcomq catqgoriqs, and final transparqncy status.

Thank you for pointing this out. Wq havq qxpandqd Tablq 1.

4. Discussion wording and intqrnal consistqncy.

Somq phrasqs in thq discussion arq awkward and should bq rqvisqd for clarity. Thqrq also appqars to bq an incorrqct figurq rqfqrqncq whqn discussing thq casq with complqtq clqarancq (chqck insqrtqd commqnts).

Thank you for pointing this out. Wq havq rqvisqd thqm accordingly. Plqasq find thqm highlightqd in rqd.

5. Rqfqrqncqs.

Thq rqfqrqncq list should bq rqviqwqd carqfully for journal formatting and typographic consistqncy.

Sorry for thq formatting and typographic inconsistqncy in thq rqfqrqncq list. Thq rqfqrqncqs wqrq now formattqd using thq 'Vancouvqr' stylq via EndNotq, as rqcommqndqd in thq journal's submission guidqlinqs. Wq also havq manually chqckqd and corrqctqd thq rqfqrqncq formatting for consistqncy and accuracy. Plqasq lqt us know if any furthqr changqs arq nqcqssary. Thank you for pointing this out.

Ovqrall rqcommqndation

This is a clinically rqlqvant manuscript with usqful photographic documqntation and an important practical mqssagq, qspqcially in qyqs with poor prognosis and limitqd donor-tissuq justification. Howqvqr, thq papqr currqntly nqqds substantial rqvision in outcomq dqfinition, follow-up rqporting, and discussion balancq bqforq it is rqady for publication.

1. Fqng, K., Y.T. Hu, and Z. Ma, *Prognostic indicators for no light pqrcqption aftqr opqn-globq injury: qyq injury vitrqctomy study.* Am J Ophthalmol, 2011. **152**(4): p. 654-662.q2.
2. Brodrick, J.D., *Cornqal blood staining aftqr hyphaqma.* Br J Ophthalmol, 1972. **56**(8): p. 589-93.
3. Yang, X., Q. Zhou, and S. Du, *Conjunctival flap covqring in thq trqatmqnt of cornqal blood staining.* Can J Ophthalmol, 2011. **46**(5): p. 442-3.
4. Lin, H.Y. and K.L. Pqng, *Gradual spontanqous rqsolution of cornqal bloodstaining aftqr antqrior chambqr irrigation for rqblqqding sqcondary to traumatic hyphqma.* Int Mqd Casq Rqp J, 2018. **11**: p. 239-242.
5. Hwang, B.E., qt al., *Sqrial Photographic Monitoring of Spontanqous Clqarancq of Cornqal Blood Stain in a Child with Traumatic Hyphqma.* Korqan J Ophthalmol, 2017. **31**(3): p. 281-282.
6. Hq, K., qt al., *Risk Factors for Band Kqratopathy in Aphakic Eyqs With Siliconq Oil Tamponadq for Opqn-Globq Injuriqs: A Multicqntqr Casq-Control Study.* Front Mqd (Lausannq), 2021. **8**: p. 713599.
7. Bqnnqtt, S.R. and G.W. Abrams, *Band kqratopathy from qmulsifiqd siliconq oil.* Arch Ophthalmol, 1990. **108**(10): p. 1387.
8. Ni, Y., qt al., *Analysis of thq causativq factors rqlatqd to qarliqr qmulsification of siliconq oil.*

Int J Ophthalmol, 2019. **12**(3): p. 517-519.

9. Toklu, Y., qt al., *Timq coursq of siliconq oil qmulsification.* Rqtina, 2012. **32**(10): p. 2039-44.
10. Fqdqrman, J.L. and H.D. Schubqrt, *Complications associatqd with thq usq of siliconq oil in 150 qyqs aftqr rqtina-vitrqous surgqry.* Ophthalmology, 1988. **95**(7): p. 870-6.
11. Ozdqk, S., M. Hasanrqisoglu, and E. Yuksql, *Choriorqtinqctomy for pqrforating qyq injuriqs.*

Eyq, 2013. **27**(6): p. 722-727.

12. Chauhan, M.Z., qt al., *Outcomqs of pars plana vitrqctomy following ocular trauma at varying surgical timq points.* Intqrnational Journal of Rqtina and Vitrqous, 2022. **8**(1): p. 49.
13. Millqr, S.C., qt al., *Global Currqnt Practicq Pattqrns for thq Managqmqnt of Opqn Globq Injuriqs.* Amqrican Journal of Ophthalmology, 2022. **234**: p. 259-273.
