# Supplementary material for: Clinical course and outcomes of corneal blood staining in open-globe injury: Eye Injury Vitrectomy Study
Source: PLoS One. 2026 Aug 4;21(8):e0354967. doi: 10.1371/journal.pone.0354967 (PMC13436741; doi:10.1371/journal.pone.0354967)
Supplement: Peer review history

# Peer review: Decision Letter 0

**DOI:** 10.1371/journal.pone.0354967.r001

**Authors:** Ugochukwu Anthony Eze

22 Apr 2026

PONE-D-26-00677

Clinical Course and Outcomes of Corneal Blood Staining in Open-Globe Injury: Eye Injury Vitrectomy Study

PLOS One

Dear Dr. Ma,

Thank you for submitting your manuscript to PLOS ONE. After careful consideration, we feel that it has merit but does not fully meet PLOS ONE’s publication criteria as it currently stands. Therefore, we invite you to submit a revised version of the manuscript that addresses the points raised during the review process.

Please submit your revised manuscript by Jun 06 2026 11:59PM. If you will need more time than this to complete your revisions, please reply to this message or contact the journal office at plosone@plos.org. When you're ready to submit your revision, log on to https://www.editorialmanager.com/pone/ and select the 'Submissions Needing Revision' folder to locate your manuscript file.

Please include the following items when submitting your revised manuscript:

- A letter that responds to each point raised by the academic editor and reviewer(s). You should upload this letter as a separate file labeled 'Response to Reviewers'.
- A marked-up copy of your manuscript that highlights changes made to the original version. You should upload this as a separate file labeled 'Revised Manuscript with Track Changes'.
- An unmarked version of your revised paper without tracked changes. You should upload this as a separate file labeled 'Manuscript'.

If you would like to make changes to your financial disclosure, please include your updated statement in your cover letter. Guidelines for resubmitting your figure files are available below the reviewer comments at the end of this letter.

If applicable, we recommend that you deposit your laboratory protocols in protocols.io to enhance the reproducibility of your results. Protocols.io assigns your protocol its own identifier (DOI) so that it can be cited independently in the future. For instructions see: https://journals.plos.org/plosone/s/submission-guidelines#loc-laboratory-protocols. Additionally, PLOS ONE offers an option for publishing peer-reviewed Lab Protocol articles, which describe protocols hosted on protocols.io. Read more information on sharing protocols at https://plos.org/protocols?utm_medium=editorial-email&utm_source=authorletters&utm_campaign=protocols.

As the corresponding author, your ORCID iD is verified in the submission system and will appear in the published article. PLOS supports the use of ORCID, and we encourage all coauthors to register for an ORCID iD and use it as well. Please encourage your coauthors to verify their ORCID iD within the submission system before final acceptance, as unverified ORCID iDs will not appear in the published article. *Only*  the individual author can complete the verification step; PLOS staff *cannot*  verify ORCID iDs on behalf of authors.

We look forward to receiving your revised manuscript.

Kind regards,

Ugochukwu Anthony Eze

Academic Editor

PLOS One

**Journal Requirements:**

When submitting your revision, we need you to address these additional requirements.

1. Please ensure that your manuscript meets PLOS ONE's style requirements, including those for file naming. The PLOS ONE style templates can be found at

https://journals.plos.org/plosone/s/file?id=wjVg/PLOSOne_formatting_sample_main_body.pdf and

https://journals.plos.org/plosone/s/file?id=ba62/PLOSOne_formatting_sample_title_authors_affiliations.pdf

2. In the online submission form you indicate that your data is not available for proprietary reasons and have provided a contact point for accessing this data. Please note that your current contact point is a co-author on this manuscript. According to our Data Policy, the contact point must not be an author on the manuscript and must be an institutional contact, ideally not an individual. Please revise your data statement to a non-author institutional point of contact, such as a data access or ethics committee, and send this to us via return email. Please also include contact information for the third party organization, and please include the full citation of where the data can be found.

3. PLOS requires an ORCID iD for the corresponding author in Editorial Manager on papers submitted after December 6th, 2016. Please ensure that you have an ORCID iD and that it is validated in Editorial Manager. To do this, go to ‘Update my Information’ (in the upper left-hand corner of the main menu), and click on the Fetch/Validate link next to the ORCID field. This will take you to the ORCID site and allow you to create a new iD or authenticate a pre-existing iD in Editorial Manager.

4. We note that Figures 1 and 2 in your submission contain copyrighted images. All PLOS content is published under the Creative Commons Attribution License (CC BY 4.0), which means that the manuscript, images, and Supporting Information files will be freely available online, and any third party is permitted to access, download, copy, distribute, and use these materials in any way, even commercially, with proper attribution. For more information, see our copyright guidelines: http://journals.plos.org/plosone/s/licenses-and-copyright.

We require you to either (1) present written permission from the copyright holder to publish these figures specifically under the CC BY 4.0 license, or (2) remove the figures from your submission:

1. You may seek permission from the original copyright holder of Figures 1 and 2 to publish the content specifically under the CC BY 4.0 license.

We recommend that you contact the original copyright holder with the Content Permission Form (http://journals.plos.org/plosone/s/file?id=7c09/content-permission-form.pdf) and the following text:

“I request permission for the open-access journal PLOS ONE to publish XXX under the Creative Commons Attribution License (CCAL) CC BY 4.0 (http://creativecommons.org/licenses/by/4.0/). Please be aware that this license allows unrestricted use and distribution, even commercially, by third parties. Please reply and provide explicit written permission to publish XXX under a CC BY license and complete the attached form.”

Please upload the completed Content Permission Form or other proof of granted permissions as an "Other" file with your submission.

In the figure caption of the copyrighted figure, please include the following text: “Reprinted from [ref] under a CC BY license, with permission from [name of publisher], original copyright [original copyright year].”

2. If you are unable to obtain permission from the original copyright holder to publish these figures under the CC BY 4.0 license or if the copyright holder’s requirements are incompatible with the CC BY 4.0 license, please either i) remove the figure or ii) supply a replacement figure that complies with the CC BY 4.0 license. Please check copyright information on all replacement figures and update the figure caption with source information. If applicable, please specify in the figure caption text when a figure is similar but not identical to the original image and is therefore for illustrative purposes only.

5. Please remove your figures from within your manuscript file, leaving only the individual TIFF/EPS image files, uploaded separately. These will be automatically included in the reviewers’ PDF.

6. If the reviewer comments include a recommendation to cite specific previously published works, please review and evaluate these publications to determine whether they are relevant and should be cited. There is no requirement to cite these works unless the editor has indicated otherwise.

**Additional Editor Comments:**

Kindly address the issues raised by the reviewers. Thank you

[Note: HTML markup is below. Please do not edit.]

Reviewers' comments:

Reviewer's Responses to Questions

**Comments to the Author**

1. Is the manuscript technically sound, and do the data support the conclusions?

The manuscript must describe a technically sound piece of scientific research with data that supports the conclusions. Experiments must have been conducted rigorously, with appropriate controls, replication, and sample sizes. The conclusions must be drawn appropriately based on the data presented.

Reviewer #1: Yes

Reviewer #2: Partly

**********

2. Has the statistical analysis been performed appropriately and rigorously?

Reviewer #1: Yes

Reviewer #2: No

**********

3. Have the authors made all data underlying the findings in their manuscript fully available?

The PLOS Data policy requires authors to make all data underlying the findings described in their manuscript fully available without restriction, with rare exception (please refer to the Data Availability Statement in the manuscript PDF file). The data should be provided as part of the manuscript or its supporting information, or deposited to a public repository. For example, in addition to summary statistics, the data points behind means, medians and variance measures should be available. If there are restrictions on publicly sharing data—e.g. participant privacy or use of data from a third party—those must be specified.

Reviewer #1: Yes

Reviewer #2: No

**********

4. Is the manuscript presented in an intelligible fashion and written in standard English?

PLOS ONE does not copyedit accepted manuscripts, so the language in submitted articles must be clear, correct, and unambiguous. Any typographical or grammatical errors should be corrected at revision, so please note any specific errors here.

Reviewer #1: Yes

Reviewer #2: Yes

**********

5. Review Comments to the Author

Please use the space provided to explain your answers to the questions above. You may also include additional comments for the author, including concerns about dual publication, research ethics, or publication ethics. (Please upload your review as an attachment if it exceeds 20,000 characters)

Reviewer #1: This article is generally well written and only requires minor revision. Some suggestions

Line 67 severe could be replaced by "the worst visual outcomes'

Line 113 , define what is an experience physician

Reviewer #2: Reviewer Comments

This manuscript addresses an uncommon but clinically relevant problem: the management of corneal blood staining in severe open-globe injury requiring posterior segment surgery. The authors present a conservative strategy in which anterior chamber washout is performed first, followed by TKP-assisted PPV with replacement of the trephined autologous cornea when visualization remains inadequate, while avoiding routine immediate allograft PKP. The topic is important, the case series is valuable for a relatively rare clinical scenario, and the photographic documentation is a strength. However, the manuscript requires major revision before it can be considered for publication. The main issues relate to incomplete characterization of cohort selection and follow-up, lack of a clearly defined corneal clearance outcome, limited analysis, underdeveloped AS-OCT reporting, and conclusions that are somewhat stronger than the data currently support.

Major comments

1. Study design and cohort selection need clearer reporting.

The manuscript states that this is a retrospective analysis of EIVS data, but it is unclear whether the 42 eyes represent a consecutive case series, a selected registry subset, or all eligible cases during a defined period (which was not stated). The authors should state the period during which the cases were selected, whether patients were consecutive, the number screened or excluded, and reasons for exclusion. A simple flow diagram would improve transparency and help readers assess selection bias and generalizability.

2. The central corneal outcome is not operationally defined.

The objective of this paper was to describe clinical course and outcome of corneal blood staining following the interventions. Yet, the paper’s main conclusion rests on “spontaneous clearance,” “corneal clarity,” and formation of a peripheral “window,” yet these outcomes are described narratively rather than systematically. The authors should define what constitutes sufficient clarity for PPV after washout, and how corneal status was graded clinically, photographically, or by AS-OCT, and whether grading was performed by one or more observers.

A formal grading system or at least reproducible categories would make the conclusions more credible.

3. Follow-up duration must be reported more adequately.

Since corneal blood staining clearance is inherently time-dependent, the actual follow-up duration of the cohort is essential. The manuscript should provide the median follow-up with IQR or range, and ideally also report time to partial clearance, time to complete clearance where achieved, and time to eventual PKP in the one transplanted eye.

4. The results should more clearly describe what happened to each subgroup.

The manuscript reports that 8 eyes achieved adequate clarity after anterior chamber washout alone, while 34 required TKP + PPV + RTC, but the later corneal course of each subgroup is not fully summarized. The authors should state:

•how many eyes achieved complete versus partial clearing,

•how many retained persistent central opacity or scarring,

•how many developed band keratopathy,

•whether all eyes eventually achieved a clinically useful fundus view,

•and whether corneal outcomes differed between the washout-only and TKP groups.

This has a tendency to further strengthen the clinical message.

5. AS-OCT is underdeveloped and should either be analyzed or toned down.

AS-OCT is included in the methods and highlighted in a representative figure, yet no cohort-level imaging findings are presented. If AS-OCT was routinely performed, the authors should summarize findings such as stromal hyperreflectivity, corneal thickness, and correlation with final corneal status. If it was not analyzable in a systematic way, then the discussion of AS-OCT’s prognostic value should be more cautious.

6. Visual outcome interpretation should be more cautious.

The manuscript reports statistically significant improvement in BCVA, but the cohort remains profoundly visually compromised overall, with many eyes presenting with NLP, many remaining NLP, and a proportion progressing to phthisis. The discussion and conclusion should avoid implying a strong functional benefit and should instead emphasize that this may be a globe-preserving and cornea-conserving strategy in eyes with otherwise poor prognosis.

7. Silicone oil status and IOP outcomes need deeper contextualization.

The paper notes that many eyes required additional silicone oil procedures and that only a few achieved complete oil removal. This is highly relevant because final IOP and eventual candidacy for corneal transplantation are likely influenced by recurrent posterior pathology, persistent hypotony, and the long-term graft-host environment. The manuscript should clarify how many eyes had secondary procedures, how many had oil refill, partial removal, or re-tamponade, whether final IOP was measured under oil in most eyes, and whether phthisical eyes were included in the IOP analysis

8. The indication and context for the single allograft PKP should be stated explicitly.

Because only one eye underwent PKP, that case is clinically informative. The manuscript should state the indication, timing, tamponade status, ocular condition, and visual/anatomical rationale for proceeding with PKP. This would help define the authors’ threshold for delayed transplantation.

9. The manuscript would benefit from association or predictor analyses.

The current analysis is mainly descriptive. Even in a modest sample, simple univariable or subgroup analyses would be useful. Potential comparisons include: washout-only versus TKP + PPV + RTC, complete versus incomplete corneal clearing, phthisis versus non-phthisis, final NLP versus retained light perception, eyes with and without useful fundus assessment, and associations with age, injury type (especially among the majority who had globe rupture), hypotony, retinal detachment, choroidal detachment, tamponade type, or no retina detected. This would strengthen the manuscript beyond a purely descriptive case series.

10. The strategy should be positioned more clearly relative to alternative approaches.

The discussion would be improved by explicitly comparing this approach with TKP plus immediate PKP, endoscopic vitrectomy, staged surgery, and observation before PPV where feasible. This would help readers understand when the proposed conservative approach is most appropriate in real-world practice.

Minor comments

1. Ethics approval inconsistency.

The ethics number differs between the submission material and the manuscript body and should be corrected.

2. Grammar and wording issues.

Several language issues need correction, including awkward or ungrammatical phrases.

3. Table 1.

Table 1 would be more informative if it included follow-up duration, number of reoperations, timing of silicone oil removal/refill, corneal outcome categories, and final transparency status.

4. Discussion wording and internal consistency.

Some phrases in the discussion are awkward and should be revised for clarity. There also appears to be an incorrect figure reference when discussing the case with complete clearance (check inserted comments).

5. References.

The reference list should be reviewed carefully for journal formatting and typographic consistency.

Overall recommendation

This is a clinically relevant manuscript with useful photographic documentation and an important practical message, especially in eyes with poor prognosis and limited donor-tissue justification. However, the paper currently needs substantial revision in outcome definition, follow-up reporting, and discussion balance before it is ready for publication.

**********

6. PLOS authors have the option to publish the peer review history of their article (what does this mean?). If published, this will include your full peer review and any attached files.

If you choose “no”, your identity will remain anonymous but your review may still be made public.

**Do you want your identity to be public for this peer review?**  For information about this choice, including consent withdrawal, please see our Privacy Policy.

Reviewer #1: **Yes:**  Annette K Hoskin

Reviewer #2: No

**********

[NOTE: If reviewer comments were submitted as an attachment file, they will be attached to this email and accessible via the submission site. Please log into your account, locate the manuscript record, and check for the action link "View Attachments". If this link does not appear, there are no attachment files.]

To ensure your figures meet our technical requirements, please review our figure guidelines: https://journals.plos.org/plosone/s/figures

You may also use PLOS’s free figure tool, NAAS, to help you prepare publication quality figures: https://journals.plos.org/plosone/s/figures#loc-tools-for-figure-preparation.

NAAS will assess whether your figures meet our technical requirements by comparing each figure against our figure specifications.

Attachment Submitted filename: PONE-D-26-00677_reviewed.docx

# Author comment: Author response to Decision Letter 1

**DOI:** 10.1371/journal.pone.0354967.r002

31 May 2026

Reviewer #1: This article is generally well written and only requires minor revision. Some suggestions

Line 67 severe could be replaced by "the worst visual outcomes'

Line 113 , define what is an experience physician

Thank you very much for the positive evaluation of our manuscript and for these helpful suggestions.

For Line 67, we have revised the sentence accordingly. (Line 49, Page 4)

“Open-globe injuries (OGIs) are ocular injuries associated with the worst visual outcomes and may result in monocular or bilateral blindness, particularly in eyes with globe rupture ”

For the definition of “experience physician”. We agree that the term “experienced physician” was not sufficiently specific and could be misleading. We intended to indicate trained clinicians who were able to align serial AS-OCT scans by using anatomical landmarks and previous scan locations as references. We have revised the Methods to clarify this point. (Line 96- 99, Page 6)

“…AS-OCT (Wavelength nanometer, Tomey CASIA2, Nagoya, Japan) was performed by trained clinicians (AD, HW, and SG), with serial scans were obtained from same corneal regions whenever possible, using anatomical landmarks and previous scan locations as references.”

Please find the revisions highlighted in red.

Reviewer #2: Reviewer Comments

This manuscript addresses an uncommon but clinically relevant problem: the management of corneal blood staining in severe open-globe injury requiring posterior segment surgery. The authors present a conservative strategy in which anterior chamber washout is performed first, followed by TKP-assisted PPV with replacement of the trephined autologous cornea when visualization remains inadequate, while avoiding routine immediate allograft PKP. The topic is important, the case series is valuable for a relatively rare clinical scenario, and the photographic documentation is a strength. However, the manuscript requires major revision before it can be considered for publication. The main issues relate to incomplete characterization of cohort selection and follow-up, lack of a clearly defined corneal clearance outcome, limited analysis, underdeveloped AS-OCT reporting, and conclusions that are somewhat stronger than the data currently support.

Thank you for your comments.

Major comments

1. Study design and cohort selection need clearer reporting.

The manuscript states that this is a retrospective analysis of EIVS data, but it is unclear whether the 42 eyes represent a consecutive case series, a selected registry subset, or all eligible cases during a defined period (which was not stated). The authors should state the period during which the cases were selected, whether patients were consecutive, the number screened or excluded, and reasons for exclusion. A simple flow diagram would improve transparency and help readers assess selection bias and generalizability.

Thanks for this valuable suggestion.

This was a retrospective analysis of cases retrieved from the EIVS database between June 2012 and June 2025. As defined by the EIVS database, the general inclusion criteria were patients who suffered from severe eye injury and were treated with vitreoretinal surgery, enucleation, or evisceration [1].

A total of 3,152 eyes in the EIVS database were screened, including 1,920 eyes with open-globe injuries. Among them, 157 eyes had documented corneal blood staining. After excluding 115 eyes because of endophthalmitis, pre-existing ocular diseases, incomplete clinical or photographic records, follow-up shorter than 12 months, or enucleation/evisceration within 12 months; 42 eyes were included in the final analysis. We have added these details to the Methods and Results, and provided a flow diagram as S1 Figure.

Please find them highlighted in red (Line 81-83, Page 5; Line 89- 90, Page 6; Line 124- 128, Page 7)

2. The central corneal outcome is not operationally defined.

The objective of this paper was to describe clinical course and outcome of corneal blood staining following the interventions. Yet, the paper’s main conclusion rests on “spontaneous clearance,” “corneal clarity,” and formation of a peripheral “window,” yet these outcomes are described narratively rather than systematically. The authors should define what constitutes sufficient clarity for PPV after washout, and how corneal status was graded clinically, photographically, or by AS-OCT, and whether grading was performed by one or more observers. A formal grading system or at least reproducible categories would make the conclusions more credible.

We appreciate this comment.

In the revised manuscript, we defined “sufficient clarity for PPV after anterior chamber washout” as intraoperative corneal clarity that allowed the surgeon to safely identify major posterior segment structures and proceed with PPV without the use of TKP. We acknowledge that this criterion inevitably contains some degree of clinical judgment, because the feasibility of PPV depends not only on the size and location of the clear corneal area, but also on the extent of posterior segment pathology, the presence of iris or blood clot obstruction, and the surgeon’s experience. In this study, PPV without TKP was attempted only when the operating surgeon (including KF, YL, HC, LH and ZZM) considered that a continuous, unobstructed corneal viewing area was sufficient to permit safe posterior segment visualization and surgical manipulation. Otherwise, TKP-assisted PPV was performed. (Line 107- 113, Page 6 and 7, highlighted in red)

We also added reproducible postoperative corneal outcome categories, including complete clearing, partial clearing, and persistent opacity. “Complete clearing” was defined as complete disappearance of opacity attributable to corneal blood staining during follow-up, although other non-blood-staining corneal changes, such as scarring or band keratopathy, could still be present. “Partial clearing” was defined as recognizable decrease of corneal blood staining compared with the preoperative status, with formation of a clear corneal area sufficient for clinical posterior segment assessment, including retinal status and intraocular tamponade status. “Persistent opacity” was defined as residual corneal opacity due to blood staining that showed little or no regression compared with the preoperative status and continued to limit corneal transparency or fundus visualization.

Serial slit-lamp photographs, fundus photographs when available, and clinical records were independently reviewed by two ophthalmologists (KF and LH) to classify the corneal outcomes. Disagreements were resolved by a senior ophthalmologist (ZZM). These definitions have been added to the Methods (Line 114- 116, Page 7), and the corresponding subgroup results have been summarized in the Results (Line 140- 143, Page 8) and Table 1.

3. Follow-up duration must be reported more adequately.

Since corneal blood staining clearance is inherently time-dependent, the actual follow-up duration of the cohort is essential. The manuscript should provide the median follow-up with IQR or range, and ideally also report time to partial clearance, time to complete clearance where achieved, and time to eventual PKP in the one transplanted eye.

Thank you for highlighting this issue. We agree that follow-up duration is essential for interpreting the natural course of corneal blood staining. Previous reports have shown substantial variability in the speed of spontaneous clearing. In traumatic hyphema-related corneal blood staining, partial clearing has been observed within weeks to months, whereas complete clearing may require 10- 15 months or even up to 2- 3 years in some cases [2-5]. This variability suggests that the course of clearance may be influenced by the severity of corneal blood staining, as well as the degree of damage to the corneal endothelium and stroma.

In our series, all eyes had trauma-related corneal blood staining and underwent anterior chamber washout during surgery; however, the degree of hyphema, preoperative IOP, interval from injury to surgery, and tamponade / anterior segment status varied among patients. These factors may have contributed to heterogeneity in the timing and extent of corneal clearing.

Accordingly, we have added the median follow-up duration of 36 months (IQR, 23- 42 months; range, 12- 60 months) to the Results (Line 135- 136, Page 8) and Table 1. We also reviewed the available clinical records and serial photographs and reported the first documented visit at which partial or complete clearing was observed (S1 Table). The timing for corneal clearing varies widely among individual patients, as it depends on the severity and extent of the pre-operative opacity , as well as the severity of the ocular trauma. Specifically, the median time to partial clearing was 3 months (IQR, 3- 7.5 months), and the median time to complete clearing was 24 months (IQR, 10- 30 months). Given the retrospective nature of this cohort, follow-up visits were not scheduled at uniform intervals; thus the exact onset of corneal clearing could not be precisely determined for every eye, and only the timing of actual follow-up visits could be verified. We have clarified this limitation in the revised manuscript. (Page 15 and 16)

For the single eye that underwent allograft PKP, we now state that PKP was performed 14 months after the initial surgery, together with the ocular status at that time (Line 173- 183, Page 9 and 10).

4. The results should more clearly describe what happened to each subgroup.

The manuscript reports that 8 eyes achieved adequate clarity after anterior chamber washout alone, while 34 required TKP + PPV + RTC, but the later corneal course of each subgroup is not fully summarized. The authors should state:

•how many eyes achieved complete versus partial clearing

•how many retained persistent central opacity or scarring

•how many developed band keratopathy

•whether all eyes eventually achieved a clinically useful fundus view,

•and whether corneal outcomes differed between the washout-only and TKP groups.

This has a tendency to further strengthen the clinical message.

Thank you for this suggestion. In the supplementary materials, we have added a subgroup analysis comparing the washout-only group and the TKP + PPV + RTC group. The numbers of eyes with complete clearing, partial clearing, and persistent opacity have been added to the Results (Line 140- 144, Page 8) and Table 1.

Persistent central opacity or scarring was observed in 17/42 eyes. In this cohort of severe OGIs, residual central opacity may have resulted from multiple factors in addition to corneal blood staining, including the original corneal wound, suture-related scarring, and band keratopathy. Final visual function was also strongly affected by lens loss and severe posterior segment injuries, such as retinal detachment, choroidal detachment, and retinal tissue loss. Therefore, the independent functional impact of central opacity could not be reliably isolated in this retrospective study.

Band keratopathy was observed in 9 of 42 eyes, all of which were tamponaded with silicone oil. Because band keratopathy has been associated with long-term silicone oil tamponade, aphakia (which may facilitate direct contact between silicone oil and the corneal endothelium), hypotony, repeated intraocular surgery, and chronic inflammation [6, 7], we did not interpret it as a direct consequence of corneal blood staining. Instead, it is reported as a postoperative corneal complication in the context of severe ocular trauma.

We further clarified whether a clinically useful fundus view was achieved in each subgroup, including eyes with complete and partial clearing. In anterior chamber washout group (n = 8), 4/8 achieved complete clearing, while 4/8 showed partial clearing. In the TKP + PPV + RTC group (n = 34), 9/34 exhibited complete clearing, 18/34 presented partial clearing, and 7/34 suffered from persistent opacity.

For eyes with corneal opacity that limited direct visualization, including during the early postoperative period (e.g., at 1 week), posterior segment assessment was performed using B-scan ultrasonography, visual function examinations, and IOP measurement. The decision to perform subsequent PKP is strictly based on a comprehensive evaluation of both visual function and the anatomical viability of the injured eye; otherwise, allogeneic PKP is not recommended. (Line 209- 213, Page 12)

The data suggested that eyes in the washout-only group generally demonstrated faster and more complete clearing of corneal blood staining (please find it in S1 Table). Given the limited sample size and baseline imbalance, these subgroup findings are presented descriptively.

5. AS-OCT is underdeveloped and should either be analyzed or toned down.

AS-OCT is included in the methods and highlighted in a representative figure, yet no cohort-level imaging findings are presented. If AS-OCT was routinely performed, the authors should summarize findings such as stromal hyperreflectivity, corneal thickness, and correlation with final corneal status. If it was not analyzable in a systematic way, then the discussion of AS-OCT’s prognostic value should be more cautious.

We agree with the reviewer. In the original manuscript, AS-OCT was overemphasized relative to the available cohort-level imaging data. Although AS-OCT was useful for documenting representative anatomical changes, imaging was not performed using a standardized quantitative protocol at fixed postoperative intervals in all eyes. Therefore, reliable cohort-level analysis of stromal hyperreflectivity, corneal thickness, or correlations with final corneal status was not feasible. In the revised manuscript, we have toned down the AS-OCT-related statements, retained AS-OCT only as representative images, and added the lack of standardized serial AS-OCT analysis as a limitation. (Page 15 and 16)

6. Visual outcome interpretation should be more cautious.

The manuscript reports statistically significant improvement in BCVA, but the cohort remains profoundly visually compromised overall, with many eyes presenting with NLP, many remaining NLP, and a proportion progressing to phthisis. The discussion and conclusion should avoid implying a strong functional benefit and should instead emphasize that this may be a globe-preserving and cornea-conserving strategy in eyes with otherwise poor prognosis.

We appreciate this comment. We agree that although BCVA showed statistical improvement, the cohort remained severely visually compromised overall, and the visual outcome was mainly limited by the severity of posterior segment injury. We have deleted this sentence in Result and revised the Discussion and Conclusion to avoid implying a strong functional benefit. The revised manuscript now emphasizes that this approach should be interpreted primarily as a globe-preserving and cornea-conserving strategy that may reduce exposure to prolonged postoperative corticosteroid or immunosuppressive therapy and lower the risk of graft rejection in eyes with limited visual potential. (Page 13, 15, 16)

7. Silicone oil status and IOP outcomes need deeper contextualization.

The paper notes that many eyes required additional silicone oil procedures and that only a few achieved complete oil removal. This is highly relevant because final IOP and eventual candidacy for corneal transplantation are likely influenced by recurrent posterior pathology, persistent hypotony, and the long-term graft-host environment. The manuscript should clarify how many eyes had secondary procedures, how many had oil refill, partial removal, or re-tamponade, whether final IOP was measured under oil in most eyes, and whether phthisical eyes were included in the IOP analysis.

Thank you for this suggestion. We agree that silicone oil status, IOP, and secondary procedures are important for interpreting final ocular status and later PKP candidacy.

In this cohort, all eyes were followed for at least 12 months. Among the 40 eyes initially tamponaded with silicone oil, only 4 achieved complete silicone oil removal by the final visit. Therefore, final IOP in most eyes was measured in the presence of silicone oil or partial silicone oil tamponade. The remaining eyes required one or more silico

Attachment Submitted filename: Response to Reviewers CBS po v2.docx

# Peer review: Decision Letter 1

**DOI:** 10.1371/journal.pone.0354967.r003

**Authors:** Ugochukwu Anthony Eze

16 Jul 2026

Clinical course and outcomes of corneal blood staining in open-globe injury: Eye Injury Vitrectomy Study

PONE-D-26-00677R1

Dear Dr. Ma,

We’re pleased to inform you that your manuscript has been judged scientifically suitable for publication and will be formally accepted for publication once it meets all outstanding technical requirements.

Within one week, you’ll receive an e-mail detailing the required amendments. When these have been addressed, you’ll receive a formal acceptance letter and your manuscript will be scheduled for publication.

An invoice will be generated when your article is formally accepted. Please note, if your institution has a publishing partnership with PLOS and your article meets the relevant criteria, all or part of your publication costs will be covered. Please make sure your user information is up-to-date by logging into Editorial Manager at Editorial Manager® and clicking the ‘Update My Information' link at the top of the page. For questions related to billing, please contact billing support.

If your institution or institutions have a press office, please notify them about your upcoming paper to help maximize its impact. If they’ll be preparing press materials, please inform our press team as soon as possible -- no later than 48 hours after receiving the formal acceptance. Your manuscript will remain under strict press embargo until 2 pm Eastern Time on the date of publication. For more information, please contact onepress@plos.org.

Kind regards,

Ugochukwu Anthony Eze

Academic Editor

PLOS One

Additional Editor Comments (optional):

Reviewers' comments:

Reviewer's Responses to Questions

**Comments to the Author**

1. If the authors have adequately addressed your comments raised in a previous round of review and you feel that this manuscript is now acceptable for publication, you may indicate that here to bypass the “Comments to the Author” section, enter your conflict of interest statement in the “Confidential to Editor” section, and submit your "Accept" recommendation.

Reviewer #2: All comments have been addressed

**********

2. Is the manuscript technically sound, and do the data support the conclusions?

The manuscript must describe a technically sound piece of scientific research with data that supports the conclusions. Experiments must have been conducted rigorously, with appropriate controls, replication, and sample sizes. The conclusions must be drawn appropriately based on the data presented.

Reviewer #2: Yes

**********

3. Has the statistical analysis been performed appropriately and rigorously?

Reviewer #2: Yes

**********

4. Have the authors made all data underlying the findings in their manuscript fully available?

The PLOS Data policy requires authors to make all data underlying the findings described in their manuscript fully available without restriction, with rare exception (please refer to the Data Availability Statement in the manuscript PDF file). The data should be provided as part of the manuscript or its supporting information, or deposited to a public repository. For example, in addition to summary statistics, the data points behind means, medians and variance measures should be available. If there are restrictions on publicly sharing data—e.g. participant privacy or use of data from a third party—those must be specified.

Reviewer #2: Yes

**********

5. Is the manuscript presented in an intelligible fashion and written in standard English?

PLOS ONE does not copyedit accepted manuscripts, so the language in submitted articles must be clear, correct, and unambiguous. Any typographical or grammatical errors should be corrected at revision, so please note any specific errors here.

Reviewer #2: Yes

**********

6. Review Comments to the Author

Please use the space provided to explain your answers to the questions above. You may also include additional comments for the author, including concerns about dual publication, research ethics, or publication ethics. (Please upload your review as an attachment if it exceeds 20,000 characters)

Reviewer #2: The revision is satisfactory overall and the previously raised concerns have been adequately addressed. The remaining issue is mainly minor language polishing, as a few grammatical inconsistencies persist (See tracked changes). These should be corrected during final editing.

**********

7. PLOS authors have the option to publish the peer review history of their article (what does this mean?). If published, this will include your full peer review and any attached files.

If you choose “no”, your identity will remain anonymous but your review may still be made public.

**Do you want your identity to be public for this peer review?**  For information about this choice, including consent withdrawal, please see our Privacy Policy.

Reviewer #2: **Yes:**  Idris Oyekunle

**********

Attachment Submitted filename: Reviewed_tracked_PONE-D-26-00677_R1.docx

# Editor report: Acceptance letter

**DOI:** 10.1371/journal.pone.0354967.r004

**Authors:** Ugochukwu Anthony Eze

PONE-D-26-00677R1

PLOS One

Dear Dr. Ma,

I'm pleased to inform you that your manuscript has been deemed suitable for publication in PLOS One. Congratulations! Your manuscript is now being handed over to our production team.

At this stage, our production department will prepare your paper for publication. This includes ensuring the following:

* All references, tables, and figures are properly cited

* All relevant supporting information is included in the manuscript submission,

* There are no issues that prevent the paper from being properly typeset

You will receive further instructions from the production team, including instructions on how to review your proof when it is ready. Please keep in mind that we are working through a large volume of accepted articles, so please give us a few days to review your paper and let you know the next and final steps.

Lastly, if your institution or institutions have a press office, please let them know about your upcoming paper now to help maximize its impact. If they'll be preparing press materials, please inform our press team within the next 48 hours. Your manuscript will remain under strict press embargo until 2 pm Eastern Time on the date of publication. For more information, please contact onepress@plos.org.

You will receive an invoice from PLOS for your publication fee after your manuscript has reached the completed accept phase. If you receive an email requesting payment before acceptance or for any other service, this may be a phishing scheme. Learn how to identify phishing emails and protect your accounts at https://explore.plos.org/phishing.

If we can help with anything else, please email us at customercare@plos.org.

Thank you for submitting your work to PLOS ONE and supporting open access.

Kind regards,

PLOS ONE Editorial Office Staff

on behalf of

Dr. Ugochukwu Anthony Eze

Academic Editor

PLOS One
